# Circulating immune landscape and immune signatures in spontaneous HIV controllers

**DOI:** 10.3389/fimmu.2025.1642482

**Published:** 2025-10-03

**Authors:** Adriana Navas, Jéssica C. dos Santos, Bram van Cranenbroek, Nadira Vadaq, Albert L. Groenendijk, Wilhelm A. J. W. Vos, Marc J. T. Blaauw, Louise van Eekeren, Casper Rokx, Janneke E. Stalenhoef, Marvin A. H. Berrevoets, Mihai G. Netea, Leo A. B. Joosten, Andre J. A. M. van der Ven, Hans J. P. M. Koenen

**Affiliations:** ^1^ Department of Internal Medicine and Radboud Center of Infectious Diseases, Radboudumc, Nijmegen, Netherlands; ^2^ Department of Laboratory Medicine, Laboratory for Medical Immunology, Radboudumc, Nijmegen, Netherlands; ^3^ Department of Internal Medicine and Department of Medical Microbiology and Infectious Diseases, ErasmusMC, Erasmus University, Rotterdam, Netherlands; ^4^ Department of Internal Medicine and Infectious Diseases, OLVG, Amsterdam, Netherlands; ^5^ Department of Internal Medicine and Infectious Diseases, Elisabeth-TweeSteden ziekenhuis, Tilburg, Netherlands; ^6^ Department of Immunology and Metabolism, Life and Medical Sciences Institute, University of Bonn, Bonn, Germany; ^7^ Department of Medical Genetics, Iuliu Hatieganu University of Medicine and Pharmacy, Cluj-Napoca, Romania

**Keywords:** HIV control, immunophenotyping, PWH, immune signature, flow cytometry

## Abstract

A subset of people with HIV, termed HIV controllers (HIC), maintain low viral loads without antiretroviral therapy. To identify the immune cell architecture of HIV control, we profiled peripheral blood from 54 HIC (including 21 elite controllers, EC) and 1,044 non-controllers (non-HIC) in the 2000HIV study (NCT03994835) using high-dimensional cytometry and confounder-adjusted regression analysis. Both HIC and EC exhibited distinct innate immune profiles compared to non-HIC, marked by reduced frequencies of CCR5^+^ NKT and TCRγδ1^+^ cells. EC further showed increased neutrophils and TCRγδ2^+^ cells, and reduced eosinophils. Unsupervised clustering revealed elevated CD11c and CD1c expression on TCRγδ2^+^ cells in EC, correlating with IFNγ production, suggesting a proinflammatory γδ T cell program unique to EC. Adaptive immune profiling showed shared features between HIC and EC: increased CD4^+^ naïve and Th1/17 cells, reduced Th17 and Tfh cells, and higher CD8^+^ TEMRA and Tc1/17 cells with reduced memory subsets. Both groups showed increased naïve and immature B cells and decreased switched memory and plasma cells. EC uniquely exhibited increased IgA^+^ memory B cells —a feature consistent with enhanced mucosal immunity— and decreased IgG^+^ memory B cells and CD307d expression, suggestive of mucosal imprinting and reduced exhaustion. Comparison of HIC and EC revealed divergent CCR5 and CXCR4 expression: EC had higher frequencies of CCR5^+^ and CXCR4^+^ CD4^+^ and CD8^+^ T cells. These elevations correlated with circulating chemokines, notably MIF for CXCR4, implying protective ligand occupancy. HIC instead showed overall lower co-receptor expression and ligand correlations. In conclusion, while HIC and EC share a core immune phenotype linked to viral control, EC-specific features— γδ T cell activation, IgA^+^ memory enrichment, and chemokine receptor regulation—may underlie more robust or distinct immune control mechanisms. This profiling resource offers new avenues for HIV cure-focused strategies.

## 1 Introduction

Most people with HIV (PWH) develop progressive immune deficiency unless antiretroviral treatment (ART) is initiated. However, a small percentage of PWH, known as HIV controllers (HIC), are able to spontaneously control HIV infection without the use of ART (1). Based on their plasma viral load and CD4^+^T cell counts, HIC are classified as elite controllers (EC) or viremic controllers (2). HIV viral control has been linked to host genetic traits, particularly those related to the HLA region and CCR5 expression (3, 4), as well as to the integration of proviruses into transcriptional silenced regions of the genome, leading to smaller HIV reservoir (5). Moreover, spontaneous control is attributed to the activity of CD8+ T cells known to target HIV-infected cells through cytolytic and non-cytolytic mechanisms (6–8). Recent studies have also highlighted the role of innate immune cells in effective immune responses among HIC, as reviewed by Thobakgale et al. (9). Notably, certain phenotypes of natural killer (NK) exhibiting enhanced effector and cytotoxic functions are more abundant in EC (10). Additionally, plasmacytoid and myeloid dendritic cells (DC) of EC demonstrate heightened type I interferon responses and increased expression of co-stimulatory molecules, facilitating robust interactions with HIV-1-specific CD8+ T-cells (11, 12). Although these studies began to identify and describe the characteristics of different immune cells associated to HIV control, a comprehensive study profiling of both innate and adaptive immune cells is lacking.

The present study aimed to characterize the circulating immune cell landscape and the functional properties of immune cells associated with spontaneous HIV control using high-dimensional flow cytometry in participants from the 2000HIV cohort, including 54 HIV controllers (HIC) and 1044 non-controllers (non-HIC). We first performed a broad assessment of immunological features in all HIC—regardless of current ART status—to capture immune signatures linked to a history of spontaneous control. Thereafter, we focused specifically on ART-naïve elite controllers (EC), representing the most stringently defined controller phenotype, to better elucidate the unconfounded immune characteristics underlying durable HIV control. EC are widely regarded as a model for a functional HIV cure, underscoring the importance of identifying the host immune factors that enable viral control in the absence of ART. To this end, we accounted for all covariates influencing immune cell population variance to identify cellular signatures linked to spontaneous control of HIV.

Overall, we observed that HIC and EC display distinct patterns of immune cell activation, exhaustion, and chemokine receptor profiles compared to non-HIC, highlighting unique immune subsets and underlying mechanisms of spontaneous HIV control. The immune profiles hereby reported contribute to the elucidation of the signatures behind this natural control that are crucial for strategies aimed at achieving a functional HIV control, and to providing a high dimensional phenotyping data resource to explore immune cell subsets and their association with various clinical phenotypes in a large cohort of PWH.

## 2 Materials and methods

### 2.1 Study design and participants

Participants were enrolled between 2019–2022 in a multicentric cross-sectional study of 1895 PWH on long-term ART, named the 2000HIV study, which includes both a discovery and a validation cohort (13). Participants of the discovery cohort were enrolled at three Dutch HIV treatment centers, (Radboudumc Nijmegen, Erasmus MC Rotterdam, and OLVG Amsterdam) and participants of the validation cohort at (Elisabeth-TweeSteden Ziekenhuis Tilburg). The inclusion criteria for the main cohort, referred to as non-HIV controllers (non-HIC) or normal progressors in our analyses, were: confirmed HIV-1 infection, age ≥18 years, receiving ART for at least six months, and a most recent plasma HIV-1 RNA level <200 copies/mL. In addition to non-HIV controllers, we included a subgroup of HIV controllers (HIC), defined as participants with a historical or current capacity to spontaneously control HIV-1, characterized by a viral load <10,000 copies/mL and stable CD4^+^ T cell counts in the absence of ART. This is an inherently heterogeneous group comprising two phenotypes:

- Elite Controllers (EC): Defined as participants with sustained HIV-1 RNA <75 copies/mL for at least 12 months without ART and stable CD4^+^ T cell counts (>500 cells/mm³), based on previously established criteria (Lambotte et al., Clin Infect Dis, 2005) (14).
- Viremic Controllers (VC): Defined as participants maintaining HIV-1 RNA <10,000 copies/mL and CD4^+^ T cell counts >500 cells/mm³ for at least five years in the absence of ART.

At the time of study sampling, 21 EC and five VC remained ART-naïve, while 25 VC and three EC were on ART, typically due to evolving treatment guidelines (post-2015 recommendations for universal ART), prevention concerns, or personal preference. For these analyses, all participants regardless of ART status were classified as HIC in the comparison of HIC vs non-HIC, whereas only ART-naïve EC were included in the EC vs non-HIC comparison.

**Figure 1:**
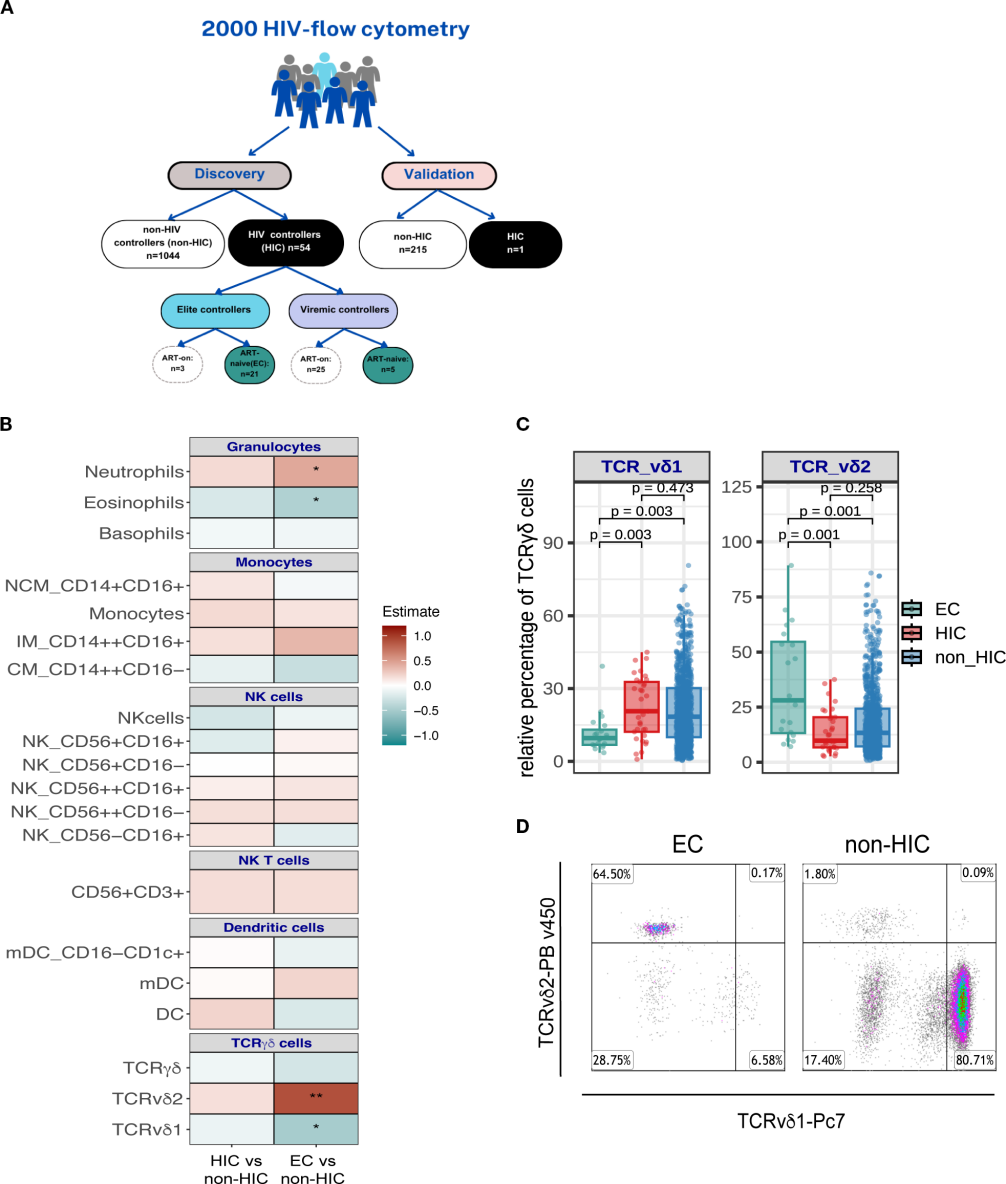
**(A)** Overview of the flow cytometry analysis in the 2000HIV cohort. The circulating immune cell phenotype was evaluated in 1,314 participants from the 2000HIV cohort to investigate the immunophenotype associated with HIV controllers (HIC), and elite controllers (EC). **(B)** Association analyses between the frequencies of innate cells and HIC (HIC), and ART naïve EC group (EC). Comparisons were performed between HIC vs. non-HIC and EC vs. non-HIC groups using a linear regression model adjusted for age, sex-birth, time to lab, seasonality, and COVID-19 vaccination status. Heatmaps display estimates from the linear regression model, with significant associations indicated (*p < 0.05, **p < 0.0001). **(C)** Comparison of the frequencies of TCRγδ1 and TCRγδ2 cells between EC (n=21), HIC (excluding ART-naïve EC, n=33) and non- HIC (n=1044). Comparisons were made using the Wilcoxon test and adjusted p values are displaying in the boxplots. **(D)** Representative flow cytometry dot plots from one EC, and one non-HIC participant illustrating the percentages of TCRγδ1 and TCRγδ2 cells relative to the parent gate (γδ T cells).

Exclusion criteria were the absence of informed consent, current viral hepatitis B or C, current pregnancy, presence of acute infection or severe communication problems. All participants provided written informed consent, and the experimental protocols were conducted following the principles of the Declaration of Helsinki. The 2000HIV study was approved by the Medical Ethical Review Committee Oost Nederland, Nijmegen, the Netherlands NL68056.091.81 and published at clinicaltrials.gov (2000HIV study - NTC03994835). For the immune cellular profiling, high dimensional flow cytometry analyses were available from 1314 out of 1895 participants (**Figure 1A**).

### 2.2 Flow cytometry procedures

Blood samples were collected by venipuncture in sterile 10 mL EDTA BD Vacutainers. Due to the multicentric nature of the study, samples were shipped overnight and processed at a central site (Radboudumc) for flow cytometry analysis. Optimization and standardization of the flow cytometry panels, as well as the staining and manual gating strategy were detailed previously (15). Briefly, an extensive immunophenotyping was performed using DURA Innovations (LUCID product line) dry reagent technology (Beckman Coulter, USA) to produce three custom-designed flow cytometry panels, containing 17–20 markers each. Samples were processed using a 21 color, six-laser CytoFLEX-LX (Beckman Coulter). Daily quality control and standardization were performed using CytoFLEX Daily QC Fluorospheres (Beckman Coulter, Catalog # B53230), CytoFLEX Daily IR QC Fluorospheres beads (Beckman Coulter, Catalog # C06147) and SPHEROtm Rainbow calibration particles 6-peak (Spherotech Inc, Catalog # RCP-30-5A-6). For data acquisition, CytExpert software 2.3 (Beckman Coulter) was used. To identify the major innate cell, T cell and B cell subsets, specific antibodies were selected (**Supplementary Tables 1**-**3**). To identify perturbations in activation, exhaustion, maturation status on immune cells, functional markers such as HLA-DR, CD38, PD1, PDL-1, CD40, CD307d and CD81 were evaluated. For data analysis, a conventional gating strategy with Kaluza V 2.1.2 software was used.

### 2.3 Unsupervised flow cytometry analysis

We performed unsupervised analysis using two algorithms: a dimensionality reduction tSNE-CUDA and clustering algorithm FlowSom (16) using the Cytobank Platform. Prior to running the analyses, anomalous events were cleaned up using the PeacoQC algorithm (17) with the recommended settings. For the tSNE-CUDA reduction algorithm the following settings were applied: equal sampling (50.000 cleaned CD45+ events/per sample) for a total of 2.1 x 10^6^ events, iteration= 3.000, perplexity=50; learning rate= 175.000 and early exaggeration = 12. We included 17 channels out of the 19 available in FCM panel 1 (**Supplementary Table 1**), CD45KrO 525 and Viakrome 808 IR885 were excluded. To integrate tSNE-cuda and FlowSOM clustering the following settings were used: clustering with the following settings: event sampling= equal, clustering method= hierarchical consensus, number of metaclusters= 20; number of clusters= 100 iterations: 10 with a random seed. The average of events per metacluster is shown in **Supplementary Table 4**.

### 2.4 Ex-vivo cytokine production measurement upon stimulation of peripheral blood mononuclear cells

PBMCs were seeded in round-bottom 96-wells plates at 0·5 x 10^6^ cells/well in 0·2 mL of RPMI Dutch modified (Life Technologies) supplemented with Gentamycin 5 mg/mL, (Centrafarm) Pyruvate 1 mM, GlutaMAX 2mM (Life Technologies) and 10% human pool serum. Cells were stimulated for 7 days at 37°C and 5% CO2 with bacterial (Streptococcus pneumoniae, Escherichia coli, Mycobacterium tuberculosis, Staphylococcus aureus), fungal (Candida albicans conidia and hyphae) and PHA. Supernatants were collected and stored at -80˚C until used for ELISA measurements. Concentrations of IFNg, IL-5, IL-10, IL-17 and IL-22, were assessed in the supernatants of the 7-day PBMC cultures, using commercial ELISA kits (Duoset ELISA, R&D Systems). The concentrations of stimuli and catalogue numbers used in the stimulation experiments are presented in **Supplementary Table 5**.

### 2.5 Plasma measurement of chemokine ligands

Plasma levels of the chemokine ligands CCL3, CCL4, CCL5, CXCL12, and MIF were quantified using a proximity extension assay (PEA) coupled with next-generation sequencing (NGS) as the detection method. Measurements were performed by Olink^®^ Proteomics AB (Uppsala, Sweden) using the Olink^®^ Explore 3072 platform, which enables high-throughput analysis of plasma proteins (18). Protein abundance is reported as Normalized Protein eXpression (NPX) values, a relative quantification unit provided by Olink that is presented on a log2 scale. These analytes were selected for their known role as ligands of CCR5 (CCL3, CCL4, CCL5) and CXCR4 (CXCL12, MIF). Correlation analyses were performed between plasma chemokine levels and the frequencies of CCR5^+^ or CXCR4^+^ CD4^+^ and CD8^+^ T cells using Spearman rank correlation.

### 2.6 Statistical data analysis

#### 2.6.1 Baseline characteristics

Comparisons of baseline characteristics were performed using Wilcoxon test for continuous variables and Pearson chi-square for categorical variables. Statistics tests and summary tables were generated using the R compareGroups package.

#### 2.6.2 Linear regression analyses

In this study a total of 355 manually annotated immune cell populations were included in the analyses (**Supplementary Figures 1**-**3**). The frequencies (relative to parent gates) from these immune cells were normalized using an inverse rank transformation (**Supplementary Figure 4**). Next, using a linear regression, we assessed the relationship between potential confounders and the first 5 principal components derived from PCA analysis of the flow cytometry data in the discovery cohort. We selected those confounders that showed an adjusted R^2^ > 0.05: age, sex-birth, time to lab (time elapsed between patient’s phlebotomy and blood sample processing in the laboratory), seasonality, and COVID-19 vaccination status (**Supplementary Figure 5**).

#### 2.6.3 HIV control phenotype and EC phenotype analyses

A linear regression model was used to compare frequencies of immune cells populations of non-HIC (n=1044), HIC (n=54) and EC (n=21). These analyses were conducted exclusively in the discovery cohort, as the validation cohort included only one HIC participant (**Figure 1A**).

Summary statistics from the linear regression models are provided in **Supplementary Table 6** for the HIC versus non-HIC comparison and in **Supplementary Table 7** for the EC versus non-HIC comparison. An association with an immune cell subset was considered significant if the nominal p-value < 0.05. All linear regression analyses included the following confounders: age, sex (female), seasonality coefficients, COVID vaccination status, and time to lab (defined as the time elapsed between the patient’s phlebotomy and blood sample processing in the laboratory).

#### 2.6.4 MFI analysis

We assessed differences in the mean intensity of fluorescence (MFI) for phenotypic, activation and exhaustion markers between HIC vs non-HIC and EC vs non-HIC using Wilcoxon test (adjusted p-value <0.05).

All statistical analyses were conducted using R version 4.3.1 (2023-06-16).

## 3 Results

### 3.1 Study population baseline characteristics

We set to assess the immune cell composition associated with HIV control status in 54 HIC, including 21 ART-naïve EC in comparison to 1044 non-HIC from the 2000HIV study, all part of the discovery cohort (**Figure 1A**). As shown in **Tables 1**, **2**, HIC and EC were slightly younger, had a higher CD4 nadir, and a larger proportion of females, compared to non-HIC (p<0.05). CMV antibody titers were also significantly lower in both HIV controllers’ groups.

**Table 1:** Clinical characteristics of HIC participants with flow cytometry data available from the discovery cohort.

	HIV controllers (HIC)	HIV non-controllers (non-hic)	*P*	N
	N=54	N=1044		
AGE (years)	48.5 [38.0;56.8]	52.0 [43.0;59.0]	0.049	1098
COHORT: DISCOVERY	54 (100%)	1044 (100%)	.	1098
SEX BIRTH:			0.019	1098
male	38 (70.4%)	873 (83.6%)		
female	16 (29.6%)	171 (16.4%)		
ETHNICITY:			0.326	1098
Asian	2 (3.70%)	55 (5.27%)		
Black	9 (16.7%)	123 (11.8%)		
ispanic	3 (5.56%)	37 (3.54%)		
Mixed	7 (13.0%)	86 (8.24%)		
White	33 (61.1%)	743 (71.2%)		
Current smoking:			1.000	1098
Non-smoker	37 (68.5%)	719 (68.9%)		
Smoker	17 (31.5%)	325 (31.1%)		
COVID VACCINATION			0.001	1098
no	24 (44.4%)	711 (68.1%)		
yes	30 (55.6%)	333 (31.9%)		
HIV DURATION (years)	12.9 [10.1;17.3]	13.1 [7.96;20.0]	0.994	1098
CART DURATION (years)	5.76 [3.12;8.12]	11.2 [6.88;17.7]	<0.001	1071*
CD4 NADIR (10^9 cells/L)	0.55 [0.45;0.68]	0.23 [0.12;0.35]	<0.001	1077*
HIC control group:				1098
EC on ART	3 (5.56%)	—		
EC	21 (38.9%)	—		
VC on ART	25 (46.3%)	—		
VC	5 (9.26%)	—		
CMV IgG Serology:			0.772	1095*
Negative	4 (7.41%)	66 (6.34%)		
Positive	50 (92.6%)	975 (93.7%)		
CMV IgG (IU/mL)	548 [288;818]	605 [337;870]	0.235	1095*
VL ZENITH (copies/mL)	9452 (27289)	607451 (3130630)	<0.001	991*

Data was analyzed using Wilcoxon test and Chi-Square (_X_2) test where applicable. (CMV) cytomegalovirus infection (IU/mL).

*Sample size for: CART DURATION (years): HIC (n=28), non-HIC (n=1043); CD4 NADIR (10^9 cells/L): HIC(n-54), non-HIC(n=1023); CMV IgG serology and titres: HIC(n=54), non-HIC(1041); VL ZENITH (copies/mL): HIC(n=37), non-HIC(n=954).

**Table 2:** Clinical characteristics of EC study participants with flow cytometry data available from the discovery cohort.

	Elite controllers (EC)	HIV non-controllers (non-hic)	*P*	N
	N=21	N=1044		
AGE	51.0 [38.0;65.0]	52.0 [43.0;59.0]	0.952	1065
COHORT: DISCOVERY	21 (100%)	1044 (100%)	.	1065
SEX_BIRTH:			0.067	1065
male	14 (66.7%)	873 (83.6%)		
female	7 (33.3%)	171 (16.4%)		
ETHNICITY:			0.937	1065
Asian	1 (4.76%)	55 (5.27%)		
Black	2 (9.52%)	123 (11.8%)		
Hispanic	1 (4.76%)	37 (3.54%)		
Mixed	2 (9.52%)	86 (8.24%)		
White	15 (71.4%)	743 (71.2%)		
Current_smoking:			0.990	1065
Non-smoker	15 (71.4%)	719 (68.9%)		
Smoker	6 (28.6%)	325 (31.1%)		
COVID VACCINATION			0.027	1065
no	9 (42.9%)	711 (68.1%)		
yes	12 (57.1%)	333 (31.9%)		
HIV DURATION (years)	12.1 [8.00;16.8]	13.1 [7.96;20.0]	0.757	1065
CART DURATION (years)	—	11.2 [6.88;17.7]	.	1043*
CD4 NADIR (10^9 cells/L)	0.65 [0.47;0.90]	0.23 [0.12;0.35]	<0.001	1044*
CMV_IgG_Serology:			0.044	1062*
Negative	4 (19.0%)	66 (6.34%)		
Positive	17 (81.0%)	975 (93.7%)		
CMV IgG (IU/mL)	407 [222;622]	605 [337;870]	0.031	1062*
VL ZENITH (copies/mL)	50.4 (76.7)	607451 (3130630)	<0.001	963 *

Data was analyzed using Wilcoxon test and Chi-Square (_X_2) test where applicable. (CMV) cytomegalovirus infection (IU/mL).

*Sample size for: CART DURATION (years): EC (n=0), non-HIC (n=1043); CD4 NADIR (10^9 cells/L): EC(n-21), non-HIC(n=1023); CMV IgG serology and titres: EC(n=21), non-HIC(1041); VL ZENITH (copies/mL): EC(n=9), non-HIC(n=954).

### 3.2 Innate compartment

#### 3.2.1 Distinct granulocyte and TCRγδ T cell profiles in HIC and EC compared to non-HIC

An overall comparison of the frequencies of the main innate immune subsets between HIC and non-HIC are reported in **Figure 1B** (left side of the heat map). A more in-depth analysis of chemokine and activation markers expressed in the immune cell subsets, revealed significantly lower frequencies of CCR5+ NK-T (CD3+CD56+) cells and CCR5+ TCRγδ1 cells in HIC compared to non-HIC (p<0.05, **Supplementary Figure 6**).

The overall innate immune signature of EC, compared to non-HIC, showed several similarities to the signature observed when HIC were compared to non-HIC, although in EC were identified more significant changes. EC displayed significantly higher frequencies of neutrophils and TCRγδ2 cells, along with lower frequencies of TCRγδ1 cells and eosinophils (**Figure 1B**, right side of the heat map). The significant alterations in the frequencies of TCRγδ1 and TCRγδ2 cells observed in EC versus non-HIC are shown in **Figures 1C, D**. EC also exhibited a significantly higher frequency of CXCR4^+^ CD56^bright^ NK cells (CD56^++^CD16^+^) and lower frequencies of HLA-DR^+^ NK cells and TCRγδ2 cells compared to non-HIC (p <0.05, **Supplementary Figure 6**).

#### 3.2.2 In depth unsupervised analysis of TCRγδ1 and TCRgdvd2 cells in elite controllers

**Figure 2:**
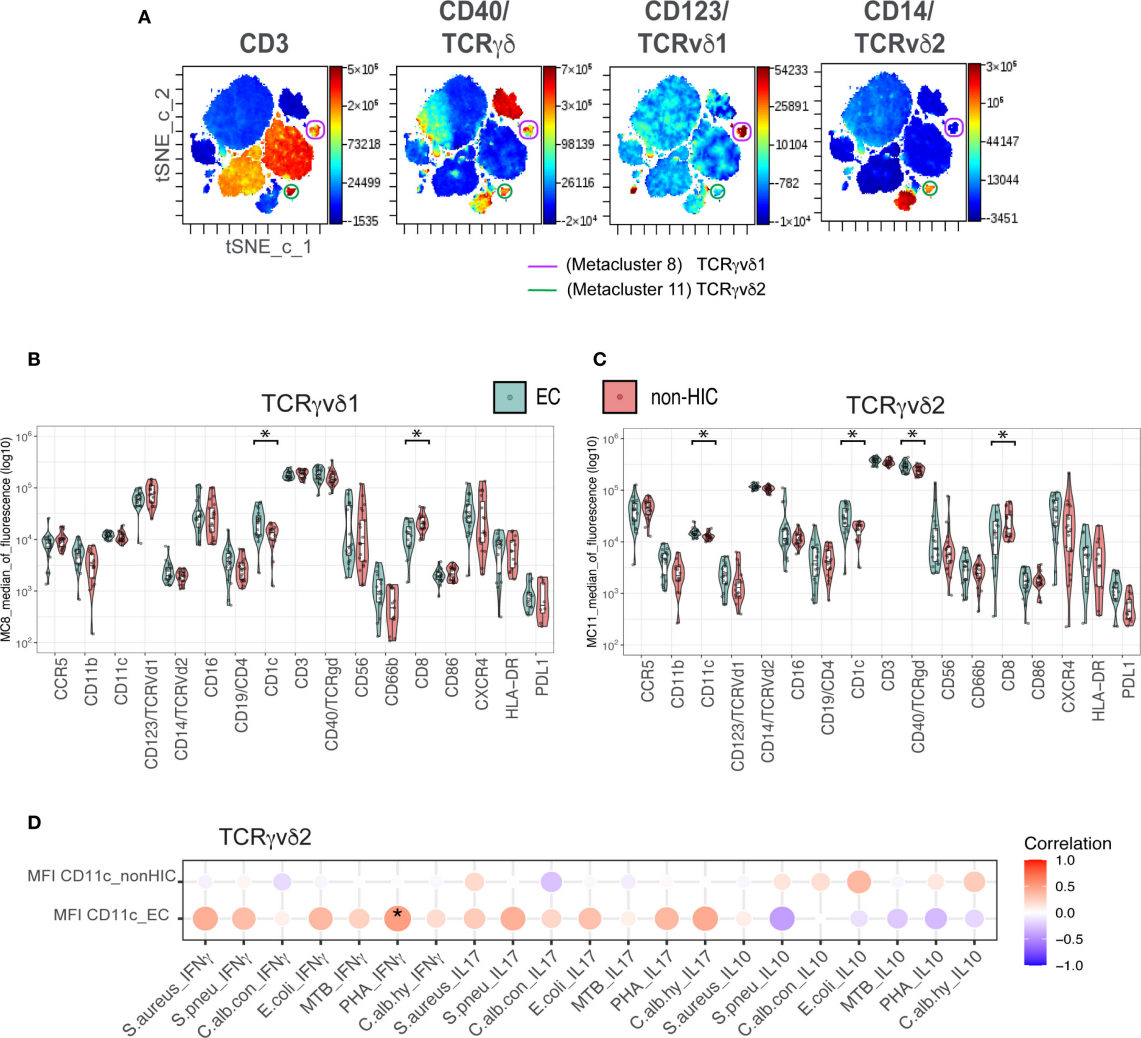
Unsupervised clustering analysis of CD45+ whole blood cells for in-depth characterization of TCRγδ cells in Elite Controllers **(A)** tSNE maps of concatenated samples from 21 elite controllers (n = 21). The same tSNE map is shown four times, each highlighting the expression intensity of different markers used to characterize TCRγδ cells (as indicated above each map) on a rainbow heat scale. Cells were clustered in a single FlowSOM run, and metaclusters 8 (purple) and 11 (green) were identified as TCRγδv1 and TCRγδv2, respectively. **(B)** Boxplots comparing the expression levels of 17 markers used for clustering, between elite controllers (EC) and non-HICs in metacluster 8 (TCRγδv1). **(C)** Boxplots comparing marker expression in metacluster 11 (TCRγδv2) between ECs and non-HICs. Comparisons were made using the Wilcoxon test (adjusted p-value < 0.05). **(D)** Balloon plot showing the correlation between CD11c expression in metacluster 11 (TCRγδv2) and ex vivo secretion of IFNγ, IL-17, and IL-10 following PBMC stimulation with various stimuli. Spearman’s rank correlation was performed, with p-values < 0.05 indicated by (*).

Given the significant alterations in the frequencies of TCRγδ1 and TCRγδ2 cells observed in (EC), we performed an in-depth phenotypic characterization of TCRγδ subsets using unsupervised FlowSOM clustering analysis. This analysis was based on the 17 markers included in our flow cytometry panel and was conducted on 21 EC and 21 non-HIC, matched for the covariates used in the regression analysis. Among the 20 identified metaclusters (MC), MC8 and MC11 correspond to TCRγδ1 and TCRγδ2 cells, respectively (**Figure 2A**, **Supplementary Figure 7A**). In accordance with our manual analysis, TCRγδ1 (MC8) abundance was lower, whereas TCRγδ2 (MC11) abundance was higher in EC compared to non-HIC (p-value < 0.05, **Supplementary Figure 7B**).

Next, we compared the mean fluorescence intensity (MFI) levels of the markers included in the panel between non-HIC and EC in both TCRγδ metaclusters. As shown in **Figures 2B, C**, significantly lower CD8 expression levels and higher CD1c expression, a molecule involved in antigen presentation, were observed in both metaclusters from EC (adjusted p-value < 0.05). Additionally, in MC11, the TCRγδ2 metacluster from EC, higher levels of CD11c and TCRγδ receptor expression were detected (adjusted p-value < 0.05, **Figure 2C**). Unconventional TCRγδ T cells are known to upregulate CD11c in response to TCRγδ activation, which is associated with cytokines production (19). To investigate this, we analyzed the correlation between CD11c expression on TCRγδ2 and ex-vivo secretion of IFNγ, IL-17, and IL-10 by PBMCs upon stimulation with various stimuli. Although a significant positive correlation for IFNγ secretion upon PHA stimulation (p < 0.05) was found only in EC, the general correlation pattern emerging is that CD11c expression in EC shows a positive correlation with the secretion of proinflammatory cytokines IFNγ and IL-17, while a negative correlation was observed for the anti-inflammatory cytokine IL-10 (**Figure 2D**).

### 3.3 Adaptive compartment

#### 3.3.1 HIC and EC exhibit distinct adaptive immune profiles compared to non-HIC

**Figure 3:**
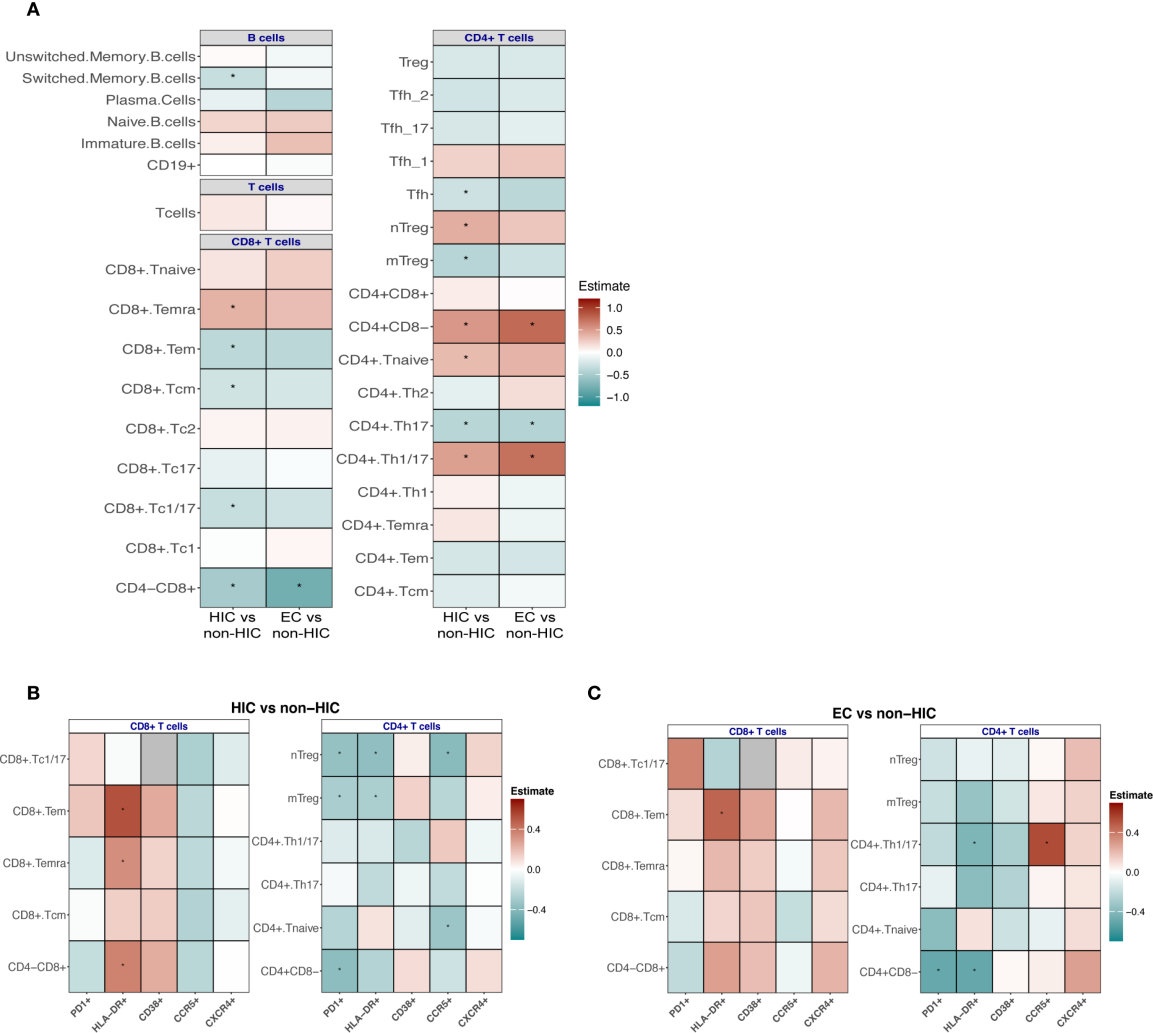
**(A)** Association analyses to compare the frequencies of adaptive immune cells between HIV controllers (HIC) vs non-HIC and EC vs non-HIC. **(B)** Comparison of the frequency of CD4+ and CD8+ Tc positive for PD1, HLA-DR, CD38, CCR5, CXCR4 markers between HIC vs non-HIC and **(C)** EC vs non-HIC. Comparisons between HIC vs non-HIC and EC vs non-HIC were tested using a linear regression model adjusted for age, sex-birth, time to lab, seasonality, and COVID-19 vaccination status. In **(A, B)** the heatmaps display the estimates from the linear regression model, with significant associations indicated (*p < 0.05, **p < 0.0001).

As with the innate compartment, the frequencies of adaptive immune cells were compared between HIC and non-HIC, as well as between EC and non-HIC (**Figure 3**). Significant differences were observed in the frequencies of various CD4+ and CD8+ T cell subsets in HIC compared to non-HIC (**Figure 3A**, left side of the heat map). Specifically, HIC exhibited higher frequencies of CD4^+^ (CD4+CD8-) T cells, CD4+ naïve T cells, CD4+ Th1/17 T cells, CD4^+^ naïve Treg, and CD8^+^TEMRA cells. Conversely, lower frequencies were observed for CD4+ Th17 T cells, CD4+ Tfh (T follicular helper) T cells, CD4+ mTreg, CD8+ T cells (CD4-CD8^+^), CD8^+^Tc1/17, CD8^+^ central memory T cells (Tcm) and CD8^+^ effector memory T cells (Tem) (**Figure 3A**, left side of the heat map).

The adaptive immune cell frequencies in ECs were similar to the landscape observed in the comparison between HIC and non-HIC (**Figure 3A**, right side of the heat map), however, fewer immune cell subsets reached statistical significance. EC demonstrated significantly higher frequencies of CD4+ (CD4+CD8-) T cells and CD4+ Th1/17 T cells, alongside significantly lower frequencies of CD8+ (CD4-CD8+) T cells and CD4+ Th17 T cells (**Figure 3A**).

The B cell signature also displayed notable changes when the groups were compared. A trend was observed toward higher frequencies of naïve and immature B cells, accompanied by reduced frequencies of switched memory B cells (significantly lower in HIC compared to non-HIC) and plasma cells (**Figure 3A**). In HIC and EC lower frequencies of IgG+ switched memory B cells were observed (significantly lower in EC compared to non-HIC) (**Supplementary Figure 6**), while in EC a trend towards higher frequencies of IgA+ switched memory B cell were found (**Supplementary Figure 6**). Frequencies of B cells expressing the exhaustion marker CD307d were significantly lower in EC (**Supplementary Figure 6**).

#### 3.3.2 Divergent activation, exhaustion and chemokine receptor profiles within and between HIC and EC

To deepen our understanding of the T cell immune profiles, we analyzed the T cell activation markers HLA-DR, CD38, and PD-1, alongside the HIV co-receptors CCR5 and CXCR4, within the previously described CD4+ and CD8+ T cell subsets in HIC and EC compared to non-HIC. These markers provide insights into immune activation, exhaustion, susceptibility to HIV entry, and migratory potential, offering further perspectives on mechanisms underpinning spontaneous viral control.

Within the CD4 T cell subsets, in both HIC and EC, lower frequencies of PD-1- and HLA-DR-expressing CD4 T cell subsets were observed compared to non-HIC (**Figures 3B, C**). Specifically, in HIC, the frequency of CD38-expressing Tregs was increased, while other CD38-expressing CD4 T cell subsets showed reduced frequencies (**Figure 3B**). In contrast, in EC, all CD38-expressing CD4^+^ T cell subsets, including Tregs, were reduced (**Figures 3B, C**). For CCR5 expressing CD4^+^ T cell subsets, reduced frequencies of CCR5-expressing CD4+ T cell subsets were observed in HIC, except for CD4 Th1/17 cells, which did not show a reduction (**Figures 3B, C**). Conversely, in EC, most CCR5-expressing CD4+ T cell subsets (excluding naïve CD4+ T cells) tended to show increased frequencies (**Figures 3B, C**). Regarding CXCR4 expressing CD4+ T cell subsets, HIC exhibited increased frequencies of CXCR4-expressing Tregs, while other CXCR4-expressing CD4+ T cell subsets were reduced, a similar pattern observed for CD38 (**Figures 3B, C**). Notably, in EC, all CXCR4-expressing CD4+ T cell subsets demonstrated increased frequencies (**Figures 3B, C**).

Within CD8 T cell subsets, in both HIC and EC, higher frequencies of HLA-DR- and CD38-expressing CD8 T cell subsets were observed compared to non-HIC (**Figures 3B, C**). Additionally, increased frequencies of PD-1-expressing CD8 Tc1/17 and CD8 Tem cells were detected (**Figures 3B, C**). Reduced frequencies of CCR5-expressing CD8 T cell subsets were observed in both HIC and EC, apart from slightly increased CCR5-expressing Tc1/17 cell frequencies in EC (**Figures 3B, C**). Regarding CXCR4 expressing CD8+ T cell subsets, HIC exhibited reduced frequencies of CXCR4-expressing CD8 T cell subsets, while EC displayed increased frequencies (**Figures 3B, C**). As shown in **Supplementary Figure 8**, the direct comparison of CCR5 and CXCR4 expressing cells between EC and HIC excluding ART-naïve EC participants, reveals a trend toward increased abundance of these cells specific in EC consistent with the observations in **Figures 3B, C**.

In addition to percentages, mean fluorescence intensity (MFI) was also assessed, revealing largely consistent findings. In HIC, significantly lower CCR5 expression levels were observed across various CD8 T cell subsets, including CD8 TEMRA, CD8 TEM, and CD8 Tc1/17 cells (adjusted p-value < 0.05, **Supplementary Table 8**). Conversely, HLA-DR expression levels were significantly increased in CD8 TEMRA and CD8 TEM subsets, while CD38 expression was elevated in CD8 Tcm cells. For CD4 T cells, significantly lower HLA-DR expression levels were noted in CD4 naïve and CD4 Th17 subsets. In contrast, EC showed no significant changes in the expression levels of the analyzed markers (**Supplementary Table 9**).

#### 3.3.3 Higher abundance CXCR4 and CCR5 expressing T cells in EC correlates to higher serum concentrations of chemokines ligands

**Figure 4:**
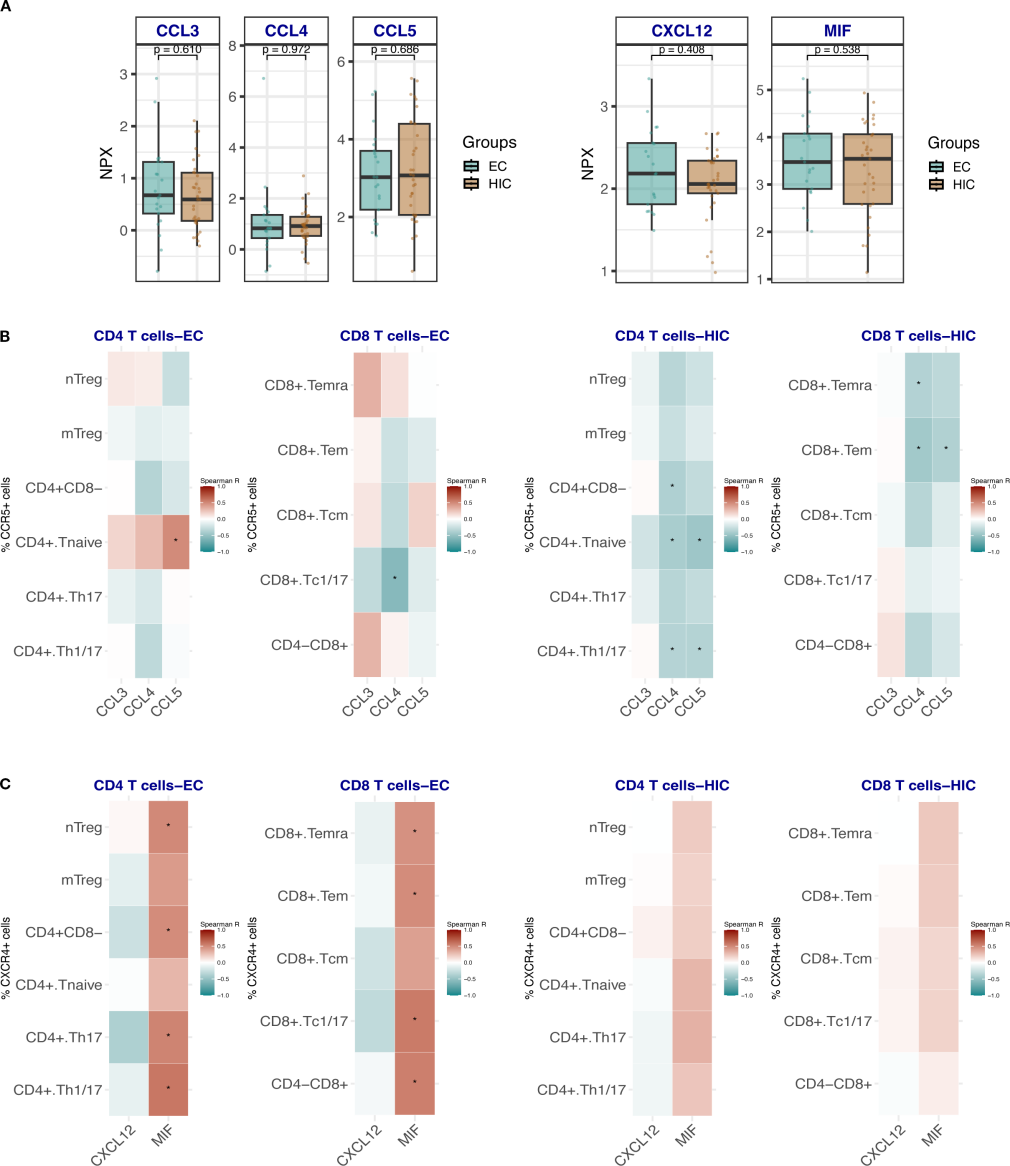
**(A)** Box plots showing the comparison of the expression levels of CCR5 and CXCR4 ligands CCL3, CCL4, CCL5 and CXCL12 and MIF respectively in HIC (excluding ART-naïve EC, n=33) and EC, n=21. Comparisons were made using the Wilcoxon test. **(B)** Heat map depicting the correlation analysis between the frequencies of CCR5-expressing CD4 Tc and CD8 Tc with the ligands CCL3, CCL4 and CCL5 in EC (left panel) and HIC (excluding ART-naïve EC) (right panel). **(C)** Heat map depicting the correlation analysis between the frequencies of CXCR4-expressing CD4 Tc and CD8 Tc with the ligands CXCL12 and MIF in EC (left panel) and HIC (excluding ART-naïve EC) (right panel). Analyses in B and C were performed using Spearman rank correlation. Red and blue colors indicate positive and negative correlations, respectively. An asterisk (*) indicates statistical significance at nominal p < 0.05.

We were particularly intrigued by the elevated frequencies of CCR5- and CXCR4-expressing T cells in EC, given that these chemokine receptors are critical co-receptors for HIV entry. We hypothesized that the persistence of these receptor-expressing T cells in EC could be due to protective mechanisms, such as elevated levels of natural chemokine ligands (e.g., CCL3, CCL4 or CCL5 in case of CCR5 and CXCL12 or MIF in case of CXCR4) that competitively inhibit viral binding. Therefore, we compared the serum levels of these ligands between EC and HIC participants (excluding ART-naïve EC). No statistically significant differences in CCL3, CCL4, CCL5, CXCL12, or MIF levels were observed between the two groups (**Figure 4A**). However, correlation analyses between CCR5-expressing T cell subsets and their ligands revealed distinct patterns. In EC (**Figure 4B**, left panel), CD4 naïve T cells showed a significant positive correlation with CCL5. In contrast, in HIC (**Figure 4B**, right panel), negative correlations were observed between CD4 naïve, Th1/Th17, CD8 Tem, and Temra cells with CCL4 and CCL5. Notably, in the EC group, significant correlations were found between CXCR4-expressing T cell populations and MIF, specifically in naïve Treg, Th17, Th1/Th17, CD8 Temra, Tem, and Tc1/17 cells (**Figure 4C**, left panel). No significant correlations were detected in HIC (**Figure 4C**, right panel).

## 4 Discussion

The primary aim of this study was to characterize the circulating immune cell landscape in HIV controllers compared to ART-exposed normal progressors. After identifying sex at birth and other variables as confounders in the association between immune cell composition and HIV control phenotype; we adjusted for these covariates and observed that both innate and adaptive immune compartments in HIV controllers display features that may contribute to spontaneous viral control.

In the innate immune compartment, HIC exhibited reduced frequencies of CCR5^+^ NKT (CD3^+^CD56^+^) cells and CCR5^+^ TCRγδ1 cells compared to non-HIC, suggesting a less activated innate profile. EC, however, demonstrated a distinct innate signature, with elevated frequencies of neutrophils, TCRγδ2 cells, and CXCR4^+^ CD56+bright NK cells, along with reduced eosinophils, TCRγδ1 cells, and HLA-DR^+^ NK cells.

Of particular interest, EC showed enrichment of TCRγδ2 and a reduction of TCRγδ1 cells, consistent with previous reports comparing elite controllers to untreated participants and ART-treated non-controllers (20, 21). TCRγδ2 cells are known to be depleted during HIV infection through HIV envelope-mediated CCR5-dependent cell death, and their recovery remains incomplete even after ART (22). In contrast, EC maintain these cells at levels comparable to uninfected participants, implicating them in natural HIV control. Our unsupervised analysis revealed that TCRγδ2 cells express higher levels of CCR5 than TCRγδ1 cells, consistent with previous findings (23). However, EC had reduced frequencies of CCR5- expressing TCRγδ2 cells, supporting the hypothesis that early protection of this subset aids their long-term preservation. Furthermore, TCRγδ2 cells from EC showed signs of activation, evidenced by elevated γδTCR and CD11c expression. CD11c is not typically expressed on T cells, but may be induced under certain conditions. A previous study in chlamydia infection demonstrated that circulating CD11c^+^ γδT cells were highly activated, with enhanced IFNγ secretion capabilities compared to CD11c- γδ T cells (19). In our study, CD11c expression levels (MFI) on TCRγδ2 cells correlated positively with IFNγ production upon PBMC stimulation in EC, suggesting enhanced antiviral functionality.

Additionally, we observed elevated CD1c expression on both TCRγδ1 and TCRγδ2 cells. Although CD1c is an Antigen-presenting cell (APC) marker involved in lipid antigen presentation, it is not normally expressed on γδT cells. This CD1c expression may be explained by trogocytosis—a process in which lymphocytes acquire membrane fragments, including surface molecules, from APCs—a phenomenon previously reported in γδT cells (24). CD1c expression in EC may endow γδT cells with APC-like properties, further amplifying their effector functions (25). Altogether, these phenotypic signatures highlight the need for deeper functional studies on γδT cells from ECs, including assessments of proliferative capacity, cytokine production (e.g., IFN-γ, TNF-α, IL-17), cytotoxic granule release (e.g., granzyme B, perforin), and the expression of maturation, activation and exhaustion markers such as CD45RA, CCR7, CD38 and PD-1. Understanding which functional phenotypes of γδ T cells are most conducive to effective viral control could provide essential mechanistic insights and support the development of γδ T cell-based immunotherapeutic strategies (26).

In the adaptive compartment, HIC exhibit elevated CD4+ naïve and Th1/17 cells, naïve Tregs, and CD8+ TEMRA cells, with reduced CD8+ memory subsets. This profile suggests preserved immune homeostasis, improved responsiveness to new antigens, and robust effector responses. EC share trends but with fewer significant changes, including higher CD4+ naïve and Th1/17 cells and reduced Th17 cells.

CD4^+^ T cells from both HIC and EC exhibited reduced signs of exhaustion and dysregulated activation, as indicated by lower frequencies of PD-1^+^ and HLA-DR^+^ cells. As reported by Nojan et al., reduced exhaustion in CD4^+^ T cells contributes to sustained viral control by preserving their proliferative capacity, cytokine production, and ability to support both CD8^+^ T cell and B cell responses (27). Moreover, PD-1^+^ memory CD4^+^ T cells have been shown to preferentially harbor latent, replication-competent HIV, suggesting that elevated PD-1 expression may promote reservoir persistence (28, 29). The observed reduction in PD-1 expression among elite controllers may therefore play a role in limiting the size of the viral reservoir, although the precise mechanisms remain incompletely understood.

HIC display unique features such as increased CD38+ and CXCR4+ Tregs and reduced CCR5+ subsets, reflecting controlled immune activation. The metabolites produced by CD38 activity contribute to adenosine generation, which can suppress effector T cell activity and inflammation. CD38+ Tregs could play a role in controlling excessive inflammation while potentially limiting viral replication through immune suppression (30).

In contrast, EC exhibit an uniform reduction in CD38^+^ CD4^+^ T cell subsets and increased frequencies of CCR5^+^ and CXCR4^+^ CD4^+^ T cells, highlighting distinct mechanisms of HIV control. Insights from cancer immunology suggest that CCR5 expression on CD4^+^ T cells enhance their capacity to support anti-tumor immunity by promoting antigen-presenting cell (APC) maturation and effective CD8^+^ T cell priming, ultimately leading to stronger cytotoxic responses (31). Translating this to the context of HIV elite control, it is plausible that CCR5^+^ CD4^+^ T cells traffic to lymph nodes, where CCR5-mediated signaling enhances their secretion of agonists and CD40L. This, in turn, facilitates full APC maturation with optimal expression of MHC-II, CD80, and CD86, thereby improving the cross-priming of CCR5^+^ CD8^+^ T cells and boosting virus-specific cytotoxic T lymphocyte (CTL) responses to maintain viral suppression, as previously proposed (31).

Similar to CCR5, the enrichment of CXCR4^+^ CD4^+^ and CD8^+^ T cells observed in EC, along with their positive correlation with plasma Macrophage migration inhibitory factor (MIF) levels, may reflect an environment that favors T cell trafficking and functional competence. MIF can bind CXCR4, forming a heteromeric complex with CD74 that promotes T cell recruitment and migration (32, 33). CXCR4 expression is also associated with a less differentiated, naïve or central memory phenotype, and inversely correlated with terminal activation or effector maturation (34). Thus, the increased frequency of CXCR4^+^ T cells in EC may reflect a less exhausted, more plastic T cell compartment capable of efficient antigen sensing and trafficking.

HIC showed an increased frequency of naïve B cells, less switched memory B cells and lower proportions of B cells expressing CD307d/Fc receptor-like 4 (FCRL4), suggesting preservation of B cell responsiveness. Availability of naïve B cells in circulation might provide to HIC an advantage to response to new antigens. In contrast, FCRL4 is a marker related to B cell disfunction and exhaustion specifically in chronic infections such as HIV (35), since it is not present on B cells from healthy subjects (36). In EC it has been proposed that reduced B cell exhaustion is a consequence of the CTL antiviral response, as rapid control of viral load favor the development of efficient HIV-specific memory B-cell responses with cross-neutralization capacity (37, 38). EC exhibit a unique B cell profile characterized by higher abundances of IgA^+^ switched memory B cells. This aligns with previous observations suggesting that HIV-specific IgA responses and affinity maturation of anti-gp41 IgA antibodies occur to a greater extent in EC than in participants on ART (39). These enhanced IgA responses may reflect a more functional and finely tuned B cell compartment in EC, potentially contributing to improved mucosal immunity and viral control. Moreover, IgA is known to bind Fcα receptor I (FcαRI/CD89), which is expressed on myeloid cells such as monocytes, neutrophils, macrophages, and certain dendritic cell subsets (40). Through this interaction, IgA can trigger a range of pro-inflammatory responses, including cytokine and chemokine release, phagocytosis, degranulation, and the formation of neutrophil extracellular traps (NETs) (41, 42). In our study, elite controllers exhibited higher frequencies of circulating neutrophils. While we did not assess CD89 expression on neutrophils directly, this finding may point to a potential link between elevated IgA responses and enhanced innate immune activation contributing to viral control. However, further *in vitro* studies are needed to experimentally validate this interaction in EC.

In conclusion, HIC and EC display distinct immune cell activation, exhaustion, and chemokine receptor profiles compared to non-HIC, highlighting unique immune subsets and underlying mechanisms of spontaneous HIV control. These distinct immune profiles underscore tailored strategies in spontaneous HIV suppression.

Finally, our study presents several key strengths. First, we performed high-dimensional flow cytometry profiling of 355 immune cell populations, offering a comprehensive overview of the circulating immune landscape in PWH. This dataset represents a valuable resource for the HIV research community to further explore immune signatures associated with viral control, as well as other clinical phenotypes. Second, we accounted for important confounding variables—including age, sex, COVID-19 vaccination status, and seasonality—thereby increasing the specificity and robustness of the identified immune associations. Third, we employed unsupervised analysis to characterize TCRγδ cells in greater depth, uncovering an activated phenotype linked to long-term viral control.

However, our study also has limitations. Notably, the observational design prevents us from establishing causality, as we report associations without functional validation of the immune profiles. Additionally, the validation cohort included only one HIC participant, which limits the strength of validation for HIC-specific findings and warrants caution in interpreting these results. Of note, since some HIC participants were receiving ART, we cannot exclude a possible influence of treatment on the observed HIC profile. While some trends did not reach statistical significance after adjustment, consistent patterns across both elite controllers (EC) and HIV controllers (HIC) reinforce the potential biological relevance of our findings.

Overall, the extensive immunophenotyping in this study provides a base for hypothesis generation and future mechanistic studies. Importantly, immunophenotyping remains a powerful tool for uncovering immune patterns associated with viral control, which may be translated or further investigated in the context of HIV cure strategies.

## Data Availability

The datasets presented in this study can be found in online repositories. The names of the repository/repositories and accession number(s) can be found below: https://doi.org/10.34973/qk29-f305, 10.34973 https://doi.org/10.34973/p96d-kz55, 10.34973 https://doi.org/10.34973/k4ka-xn94, 10.34973.
