# Supplementary material for: Circulating immune landscape and immune signatures in spontaneous HIV controllers
Source: Front Immunol. 2025 Oct 3;16:1642482. doi: 10.3389/fimmu.2025.1642482 (PMC12532132; doi:10.3389/fimmu.2025.1642482)
Supplement: Supplementary file 1 [file Table1.docx]

**Supplementary TABLE 1. Description of the antibodies in panel 1 (general panel)**

| Specificity | Fluorochrome | Clone | Cat. Number | Company | µl  per donor | Purpose |
| --- | --- | --- | --- | --- | --- | --- |
| CD3 | BUV395 | UCTH1 | 563546 | BD Biosciences | 5 | T-cell lineage |
| HLA-DR | BUV661 | G46-6 | 612980 | BD Biosciences | 5 | Activation marker |
| CD86* | BUV737 | FUN-1 | 612784 | BD Biosciences | 2 | Costimulatory in APCs |
| CD14/  TCRVd2 | PB | RM052/  IMMU389 | ---- | ---- | ---- | CD14: Monocyte marker/ TCRVd2: T cell receptor expression for variant d2 |
| CD45 | KrO | J33 | ---- | ---- | ---- | White blood cells lineage |
| CD11c* | BV605 | 3.9 | 301636 | BioLegend | 5 | Classical dendritic cells |
| CD11b | BV650 | ICRF44 | 301336 | BioLegend | 10 | Integrin marker, expressed mainly in innate cells |
| CD274  (PDL1) | BV786 | 29E.2A3 | 329736 | BioLegend | 5 | Constitutive, low levels expressed on resting lymphocytes and APCs. immunoregulatory PD-1/PD-L1 pathway is operative during a persistent viral infection in humans, and define a reversible defect in HIV-specific T-cell function. |
| CD16 | FITC | 3G8 | ---- | ---- | ---- | NK subsets distinction, neutrophils and monocytes subsets |
| CD8 | PerCP | RPA-T8 | 301030 | BioLegend | 1 | T-cell lineage |
| CD184  (CXCR4) | PE | 12G5 | ---- | ---- | ---- | Th marker |
| CD56 | ECD | N901NKH-1 | ---- | ---- | ---- | NK cell lineage/NKT-like cell |
| CD1c | PC5 | L161 | ---- | ---- | ---- | Myeloid dendritic cells. CD1c+ DCs are susceptible to HIV infection, enables viral antigen production but impairs their immune functions and survival. |
| CD40/  TCR PAN gd | PC5.5 | MAB89/IMMU510 | ---- | ---- | ---- | CD40: B cells, iDCs and follicular DCs/ CD40 signaling may regulate MBC subset development.  TCR PAN gd: gd T-cell lineage |
| CD123/  TCRVd1 | PC7 | SSDCLY107D2/R9.12 | ---- | ---- | ---- | Plasmocytoid DCs, Basophils/  T cell receptor expression for variant d1: important in intestinal barrier function, increase in frequency in HIV infected individuals, including HIV controllers. |
| CD195  (CCR5) | APC | J418F1 | 359122 | BioLegend | 2 | HIV co-receptor (T cells, NK cells and Monocytes) |
| CD19/  CD4 | AA700 | J3.119/13B8.2 | ---- | ---- | ---- | CD19: B-cell lineage /CD4: T-cell lineage |
| CD66b | AA750 | 80H3 | ---- | ---- | ---- | Neutrophils |
| ViaKrome | IR885/40 | ---- | C36628 | Beckman  Coulter | --- | Selection of viable cells |
| PBA  (1% BSA in PBS) | ---- | ---- | ---- | ---- | 42 | Washing cells |
| Brillian stain buffer | ---- | ---- | 563794 | BD Biosciences | 50 | Required for staining with polymer dyes (BV abs) |

**Supplementary TABLE 2. Description of the antibodies in panel 2 (T cell panel)**

| Specificity | Fluorochrome | Clone | Cat. Number | Company | µl  per donor | Purpose |
| --- | --- | --- | --- | --- | --- | --- |
| CD197  (CCR7) | BUV395 | 2-L1-A | 749655 | BD Biosciences | 20 | Naïve cell marker |
| HLA-DR | BUV661 | G46-6 | 612980 | BD Biosciences | 5 | Activation marker |
| CD56 | BUV737 | NCAM16.2 | 612766 | BD Biosciences | 1 | NK cell linage/NKT-like cell |
| CD8 | PacBlue | B9.11 | ---- | ---- | ---- | CD8 T cell linage (co-expressed with CD3) |
| CD45 | KrO | J33 | ---- | ---- | ---- | White blood cell linage |
| CD185  (CXCR5) | BV605 | J252D4 | 356930 | BioLegend | 5 | Tfh marker |
| CD154  (CD40L) | BV650 | 24-31 | 310842 | BioLegend | 1 | Activation marker. Expressed on T cells under inflammatory conditions |
| CD38 | BV786 | HB-7 | 356620 | BioLegend | 2 | Activation marker. Co-expression with HLADR in Tcm cells is associated HIV persistence |
| CD183  (CXCR3) | AF488 | G025H7 | ---- | ---- | ---- | Tfh/Th marker |
| CD25 | ECD | B1.49.9 | ---- | ---- | ---- | Regulatory T cell linage |
| CD3 | PerCP | UCHT1 | 300428 | BioLegend | 10 | T cell linage |
| CD184  (CXCR4) | PE | 12G5 | ---- | ---- | ---- | HIV co-receptor |
| CD4 | AF594 | RPA-T4 | 300544 | BioLegend | 5 | CD4 T cell linage (co-expressed with CD3) |
| CD194  (CCR4) | PC5 | L291H4 | ---- | ---- | ---- | Th marker |
| CD279  (PD1) | PC5.5 | PD1.3 | ---- | ---- | ---- | Exhaustion marker. Up-regulated upon HIV infection, related to T cells immunosenescence. |
| CD196  (CCR6) | PC7 | B-R35 | ---- | ---- | ---- | Tfh/Th marker |
| CD195  (CCR5) | APC | J418F1 | 359122 | BioLegend | 2 | HIV co-receptor (T cells, NK cells and Monocytes) |
| CD127 | APC-A700 | R34.34 | ---- | ---- | ---- | Memory and effector T cells |
| CD45RA | APC-A750 | 2H4LDH11LDB9 | ---- | ---- | ---- | Naïve cell marker |
| ViaKrome | IR885/40 | ---- | C36628 | Beckman  Coulter | --- | Selection of viable cells |
| PBA  (1% BSA in PBS) | ---- | ---- | ---- | ---- | 19 | Washing cells |
| Brillian stain buffer | ---- | ---- | 563794 | BD Biosciences | 50 | Required for staining with polymer dyes (BV abs) |

**Supplementary TABLE 3. Description of the antibodies in panel 3 (B cell panel)**

| Specificity | Fluorochrome | Clone | Cat. Number | Company | µl  per donor | Purpose |
| --- | --- | --- | --- | --- | --- | --- |
| IgG | BUV395 | G18-145 | 564229 | BD Biosciences | 10 | --- |
| CD19 | BUV737 | SJ25C1 | 612756 | BD Biosciences | 2 | B cell lineage |
| IgM | PacBlue | SA-DA4 | ---- | ---- | ---- | --- |
| CD45 | KrO | J33 | ---- | ---- | ---- | White blood cells lineage |
| CD27 | BV605 | O323 | 302830 | BioLegend | 2 | Activation marker |
| CD307d | BV650 | A1 | 747557 | BD Biosciences | 10 | Expression in memory B cells is associated with exhaustion in HIV-infected viremic individuals |
| CD20 | BV785 | 2H7 | 302356 | BioLegend | 5 | B cell lineage |
| CD81 | FITC | JS64 | ---- | ---- | ---- | Co-stimulatory molecule, regulates CD19 expression in B lymphocytes. |
| IgD | PEdazzle | 581/IA6-2 | 348240 | BioLegend | 2 | ---- |
| CD21 | PE | BL13 | ---- | ---- | ---- | Subset of memory B cells |
| CD34 | ECD | 581 | ---- | ---- | ---- | Immature cell marker |
| CD24 | PC5 | ALB9 | ---- | ---- | ---- | Maturation marker |
| CD138 | PC5.5 | B-A38 | ---- | ---- | ---- | Plasma cell marker |
| IgE | PC7 | E124.2.8 | ---- | ---- | ---- | ---- |
| IgA | APC | REA1014 | 130-113-998 | Miltenyi Biotech | 5 | ---- |
| CD10 | APC-A700 | ALB1 | ---- | ---- | ---- | Differentiation of B cells |
| CD38 | APC-A750 | LS198-4-3 | ---- | ---- | ---- | Activation marker |
| ViaKrome | IR885/40 | ----- | C36628 | Beckman  Coulter | ---- | Selection of viable cells |
| PBA  (1% BSA in PBS) | ---- | ---- | ---- | ---- | 34 | Washing cells |
| Brillian stain buffer | ---- | ---- | 563794 | BD Biosciences | 50 | Required for staining with polymer dyes (BV abs) |

**Supplementary TABLE 4. Event counts per metacluster obtained with FlowSom dimentional reduction algorithm in Elite Controllers and HIV non controllers.**

| Metacluster ID | EC, n=21  (event count-  mean) | non-HIC,  n=21 (event count-mean) | EC  (event count-SD) | non-HIC  (event count-SD) |
| --- | --- | --- | --- | --- |
| 1 | 12780 | 10320 | 5073 | 3310 |
| 2 | 159.5 | 99.71 | 188.2 | 105.4 |
| 3 | 19570 | 18930 | 8318 | 6384 |
| 4 | 304 | 526.5 | 294.1 | 462.4 |
| 5 | 83.71 | 57.76 | 71.28 | 41.55 |
| 6 | 145.8 | 139.3 | 81.22 | 54.37 |
| 7 | 123 | 148.6 | 61.51 | 67.63 |
| 8 | 252.2 | 651.4 | 262.3 | 915.9 |
| 9 | 685 | 101 | 2546 | 54.73 |
| 10 | 3008 | 3009 | 1615 | 1426 |
| 11 | 410.1 | 110.2 | 730.6 | 160 |
| 12 | 2401 | 3339 | 1566 | 2819 |
| 13 | 208.4 | 249.8 | 141.3 | 164.6 |
| 14 | 7439 | 9603 | 2545 | 4443 |
| 15 | 356.1 | 430.8 | 208.8 | 340.3 |
| 16 | 209.2 | 116 | 253.5 | 84.91 |
| 17 | 111.1 | 98.52 | 61.61 | 51.08 |
| 18 | 1461 | 1701 | 780.7 | 692.6 |
| 19 | 79.62 | 108.5 | 54.45 | 41.83 |
| 20 | 219 | 258.9 | 138.4 | 121.7 |

**Supplementary Table 5. Stimulation scheme for evaluation of 7 days ex-vivo** **cytokine production**

| PBMC 7-day stimulation experiments | | | |
| --- | --- | --- | --- |
| Stimulus | Final Concentration | Manufacturer | Cat. no |
| 1. RPMI | NA | Gibco | 22409031 |
| 1. E. coli | 1x10^6^/mL | In-house production | Strain: ATCC35218 |
| 1. S· aureus | 1x10^6^/mL | In-house production | Strain: ATCC29213 |
| 1. S. Pneumoniae | 5x10^6^/mL | In-house production | Strain: ATCC49619 |
| 1. M. tuberculosis | 5 µg/mL | BEI Resources - H37Rv | NR-14822 |
| 1. C. albicans (conidia) | 1x10^6^/mL | In-house production | Strain: UC820 |
| 1. PHA | 10 µg/mL | Sigma-Aldrich | L9017-5MG |
| 1. C. albicans (hyphae) | 1x10^6^/mL | In-house production | Strain: UC820 |

**Supplementary TABLE 6. Summary statistics from linear regression model comparing HIC vs non-HIC**

| Cell subset | Comparison | Estimate | P-value | Stars |
| --- | --- | --- | --- | --- |
| **Panel2_2018_CD4+CD8-** | **HIC_vs_non-HIC** | **0.526** | **<0.001** | ***** |
| **Panel2_2349_CD4+CD8-_CCR4+** | **HIC_vs_non-HIC** | **-0.518** | **<0.001** | ***** |
| **Panel2_2336_CD8+.Tem_HLA-DR+** | **HIC_vs_non-HIC** | **0.518** | **<0.001** | ***** |
| **Panel2_2017_CD4-CD8+** | **HIC_vs_non-HIC** | **-0.502** | **<0.001** | ***** |
| **Panel2_2530_Treg_CCR7+** | **HIC_vs_non-HIC** | **0.485** | **<0.001** | ***** |
| **Panel2_2337_CD8+.Tc1_HLA-DR+** | **HIC_vs_non-HIC** | **0.499** | **<0.001** | ***** |
| **Panel2_2356_Treg_CCR4+** | **HIC_vs_non-HIC** | **-0.448** | **<0.001** | ***** |
| **Panel2_2055_CD4+.Th1/17** | **HIC_vs_non-HIC** | **0.482** | **<0.001** | ***** |
| **Panel2_2358_nTreg_CCR4+** | **HIC_vs_non-HIC** | **-0.415** | **0.001** | ***** |
| **Panel2_2024_mTreg** | **HIC_vs_non-HIC** | **-0.409** | **0.001** | ***** |
| **Panel2_2025_nTreg** | **HIC_vs_non-HIC** | **0.409** | **0.001** | ***** |
| **Panel2_2416_nTreg_CCR5+** | **HIC_vs_non-HIC** | **-0.390** | **0.002** | ***** |
| **Panel2_2092_CD8+HLA-DR+CD38+** | **HIC_vs_non-HIC** | **0.417** | **0.003** | ***** |
| **Panel2_2056_CD4+.Th17** | **HIC_vs_non-HIC** | **-0.397** | **0.003** | ***** |
| **Panel2_2298_Treg_HLA-DR+** | **HIC_vs_non-HIC** | **-0.387** | **0.003** | ***** |
| **Panel2_2532_nTreg_CCR7+** | **HIC_vs_non-HIC** | **0.400** | **0.003** | ***** |
| **Panel2_2124_Treg_PD1+** | **HIC_vs_non-HIC** | **-0.362** | **0.003** | ***** |
| **Panel2_2060_CD8+.Tem** | **HIC_vs_non-HIC** | **-0.379** | **0.004** | ***** |
| **Panel2_2078_CD4+PD1+** | **HIC_vs_non-HIC** | **-0.374** | **0.005** | ***** |
| **Panel2_2300_nTreg_HLA-DR+** | **HIC_vs_non-HIC** | **-0.363** | **0.005** | ***** |
| **Panel2_2061_CD8+.Temra** | **HIC_vs_non-HIC** | **0.385** | **0.005** | ***** |
| **Panel2_2467_CD4+CD8+_CCR6+** | **HIC_vs_non-HIC** | **0.372** | **0.006** | ***** |
| **Panel2_2126_nTreg_PD1+** | **HIC_vs_non-HIC** | **-0.351** | **0.006** | ***** |
| **Panel2_2374_CD4+.Tem_CCR4+** | **HIC_vs_non-HIC** | **-0.381** | **0.006** | ***** |
| **Panel1_1340_TCRvd1_CCR5+** | **HIC_vs_non-HIC** | **-0.367** | **0.007** | ***** |
| **Panel2_2371_CD4+.Tcm_CCR4+** | **HIC_vs_non-HIC** | **-0.373** | **0.008** | ***** |
| **Panel2_2292_CD4-CD8+_HLA-DR+** | **HIC_vs_non-HIC** | **0.371** | **0.008** | ***** |
| **Panel2_2046_CD4+.Tnaive** | **HIC_vs_non-HIC** | **0.344** | **0.009** | ***** |
| **Panel2_2531_mTreg_CCR7+** | **HIC_vs_non-HIC** | **0.353** | **0.011** | ***** |
| **Panel2_2414_Treg_CCR5+** | **HIC_vs_non-HIC** | **-0.314** | **0.012** | ***** |
| **Panel2_2357_mTreg_CCR4+** | **HIC_vs_non-HIC** | **-0.332** | **0.014** | ***** |
| **Panel2_2335_CD8+.Temra_HLA-DR+** | **HIC_vs_non-HIC** | **0.336** | **0.017** | ***** |
| **Panel2_2430_CD4+.Tnaive_CCR5+** | **HIC_vs_non-HIC** | **-0.328** | **0.017** | ***** |
| **Panel2_2474_nTreg_CCR6+** | **HIC_vs_non-HIC** | **-0.304** | **0.017** | ***** |
| **Panel2_2554_CD4+.Th2_CCR7+** | **HIC_vs_non-HIC** | **0.329** | **0.020** | ***** |
| **Panel2_2603_CD4+.Tcm_CXCR3+** | **HIC_vs_non-HIC** | **-0.321** | **0.020** | ***** |
| **Panel2_2141_CD4+.Temra_PD1+** | **HIC_vs_non-HIC** | **-0.313** | **0.021** | ***** |
| **Panel2_2125_mTreg_PD1+** | **HIC_vs_non-HIC** | **-0.282** | **0.022** | ***** |
| **Panel2_2073_CD8+.Tc1/17** | **HIC_vs_non-HIC** | **-0.318** | **0.022** | ***** |
| **Panel2_2590_nTreg_CXCR3+** | **HIC_vs_non-HIC** | **-0.274** | **0.022** | ***** |
| **Panel2_2472_Treg_CCR6+** | **HIC_vs_non-HIC** | **-0.290** | **0.023** | ***** |
| **Panel3_3029_Switched.Memory.B.cells** | **HIC_vs_non-HIC** | **-0.316** | **0.023** | ***** |
| **Panel2_2279_CD8+.Tc1_CD38+** | **HIC_vs_non-HIC** | **0.282** | **0.030** | ***** |
| **Panel2_2406_CD56+CD3+_CCR5+** | **HIC_vs_non-HIC** | **-0.293** | **0.036** | ***** |
| **Panel2_2299_mTreg_HLA-DR+** | **HIC_vs_non-HIC** | **-0.280** | **0.038** | ***** |
| **Panel2_2030_Tfh** | **HIC_vs_non-HIC** | **-0.286** | **0.039** | ***** |
| **Panel2_2059_CD8+.Tcm** | **HIC_vs_non-HIC** | **-0.274** | **0.040** | ***** |
| **Panel2_2746_CD8+.Tc1/17_CXCR5+** | **HIC_vs_non-HIC** | **-0.276** | **0.043** | ***** |
| **Panel2_2142_CD4+.Tem_PD1+** | **HIC_vs_non-HIC** | **-0.268** | **0.046** | ***** |
| **Panel3_3097_Immature.B.cells_CD81+** | **HIC_vs_non-HIC** | **0.229** | **0.050** | **ns** |
| **Panel2_2456_CD8+.Tc1/17_CCR5+** | **HIC_vs_non-HIC** | **-0.274** | **0.051** | **ns** |
| **Panel2_2036_Tfh_2** | **HIC_vs_non-HIC** | **-0.262** | **0.052** | **ns** |
| **Panel2_2449_CD8+.Tcm_CCR5+** | **HIC_vs_non-HIC** | **-0.254** | **0.055** | **ns** |
| **Panel2_2088_CD4+HLA-DR+CD38+** | **HIC_vs_non-HIC** | **-0.257** | **0.055** | **ns** |
| **Panel2_2581_CD4+CD8-_CXCR3+** | **HIC_vs_non-HIC** | **-0.255** | **0.057** | **ns** |
| **Panel2_2234_CD4-CD8+_CD38+** | **HIC_vs_non-HIC** | **0.245** | **0.057** | **ns** |
| **Panel2_2278_CD8+.Tem_CD38+** | **HIC_vs_non-HIC** | **0.252** | **0.058** | **ns** |
| **Panel1_1147_CM_CD14++CD16-_CD86+** | **HIC_vs_non-HIC** | **-0.230** | **0.058** | **ns** |
| **Panel2_2266_CD4+.Th1/17_CD38+** | **HIC_vs_non-HIC** | **-0.232** | **0.059** | **ns** |
| **Panel2_2087_CD4+HLA-DR+CD38-** | **HIC_vs_non-HIC** | **-0.238** | **0.061** | **ns** |
| **Panel2_2739_CD8+.Tcm_CXCR5+** | **HIC_vs_non-HIC** | **-0.257** | **0.062** | **ns** |
| **Panel2_2523_CD4+CD8-_CCR7+** | **HIC_vs_non-HIC** | **0.254** | **0.064** | **ns** |
| **Panel2_2415_mTreg_CCR5+** | **HIC_vs_non-HIC** | **-0.232** | **0.064** | **ns** |
| **Panel2_2091_CD8+HLA-DR+CD38-** | **HIC_vs_non-HIC** | **0.246** | **0.065** | **ns** |
| **Panel2_2453_CD8+.Tc1_CCR5+** | **HIC_vs_non-HIC** | **-0.244** | **0.067** | **ns** |
| **Panel2_2039_Tfh_1/17** | **HIC_vs_non-HIC** | **0.244** | **0.069** | **ns** |
| **Panel2_2140_CD4+.Tnaive_PD1+** | **HIC_vs_non-HIC** | **-0.246** | **0.069** | **ns** |
| **Panel2_2438_CD4+.Th2_CCR5+** | **HIC_vs_non-HIC** | **-0.222** | **0.071** | **ns** |
| **Panel2_2014_NKcells** | **HIC_vs_non-HIC** | **-0.251** | **0.071** | **ns** |
| **Panel2_2606_CD4+.Tem_CXCR3+** | **HIC_vs_non-HIC** | **0.245** | **0.073** | **ns** |
| **Panel2_2100_CD8+CXCR4+CCR5+** | **HIC_vs_non-HIC** | **-0.206** | **0.073** | **ns** |
| **Panel2_2291_CD4+CD8-_HLA-DR+** | **HIC_vs_non-HIC** | **-0.247** | **0.074** | **ns** |
| **Panel3_3068_NBC_CD21+** | **HIC_vs_non-HIC** | **-0.250** | **0.076** | **ns** |
| **Panel1_1227_Tc_CD4+CD8-_PDL1+** | **HIC_vs_non-HIC** | **-0.211** | **0.077** | **ns** |
| **Panel2_2408_CD4-CD8+_CCR5+** | **HIC_vs_non-HIC** | **-0.228** | **0.078** | **ns** |
| **Panel2_2139_CD4+.Tcm_PD1+** | **HIC_vs_non-HIC** | **-0.233** | **0.081** | **ns** |
| **Panel2_2040_Tfh_17** | **HIC_vs_non-HIC** | **-0.229** | **0.081** | **ns** |
| **Panel2_2044_CD4+.Tem** | **HIC_vs_non-HIC** | **-0.237** | **0.084** | **ns** |
| **Panel3_3070_IM.(CD27-CD21+)** | **HIC_vs_non-HIC** | **-0.238** | **0.087** | **ns** |
| **Panel1_1148_IM_CD14++CD16+_CD86+** | **HIC_vs_non-HIC** | **-0.203** | **0.089** | **ns** |
| **Panel1_1193_Neutrophils_PDL1+** | **HIC_vs_non-HIC** | **-0.196** | **0.090** | **ns** |
| **Panel3_3109_Switched.Memory.B.cells_CD307d+** | **HIC_vs_non-HIC** | **-0.207** | **0.094** | **ns** |
| **Panel1_1173_Tc_CD4+CD8-_CD86+** | **HIC_vs_non-HIC** | **-0.225** | **0.094** | **ns** |
| **Panel2_2452_CD8+.Tem_CCR5+** | **HIC_vs_non-HIC** | **-0.226** | **0.095** | **ns** |
| **Panel2_2439_CD4+.Th17_CCR5+** | **HIC_vs_non-HIC** | **-0.205** | **0.096** | **ns** |
| **Panel2_2373_CD4+.Temra_CCR4+** | **HIC_vs_non-HIC** | **-0.229** | **0.098** | **ns** |
| **Panel2_2507_CD8+.Tcm_CCR6+** | **HIC_vs_non-HIC** | **-0.226** | **0.098** | **ns** |
| **Panel2_2451_CD8+.Temra_CCR5+** | **HIC_vs_non-HIC** | **-0.217** | **0.098** | **ns** |
| **Panel2_2035_Tfh_1** | **HIC_vs_non-HIC** | **0.224** | **0.099** | **ns** |
| **Panel2_2740_CD8+.Tnaive_CXCR5+** | **HIC_vs_non-HIC** | **-0.225** | **0.101** | **ns** |
| **Panel2_2556_CD4+.Th1/17_CCR7+** | **HIC_vs_non-HIC** | **-0.227** | **0.102** | **ns** |
| **Panel1_1341_TCRvd2_CCR5+** | **HIC_vs_non-HIC** | **-0.224** | **0.103** | **ns** |
| **Panel2_2021_Treg** | **HIC_vs_non-HIC** | **-0.223** | **0.104** | **ns** |
| **Panel2_2450_CD8+.Tnaive_CCR5+** | **HIC_vs_non-HIC** | **-0.208** | **0.104** | **ns** |
| **Panel3_3098_Naive.B.cells_CD81+** | **HIC_vs_non-HIC** | **0.223** | **0.107** | **ns** |
| **Panel2_2094_CD4+CXCR4-CCR5+** | **HIC_vs_non-HIC** | **-0.210** | **0.108** | **ns** |
| **Panel2_2098_CD8+CXCR4-CCR5+** | **HIC_vs_non-HIC** | **-0.201** | **0.113** | **ns** |
| **Panel1_1012_Eosinophils** | **HIC_vs_non-HIC** | **-0.223** | **0.113** | **ns** |
| **Panel1_1226_Tc_PDL1+** | **HIC_vs_non-HIC** | **-0.178** | **0.121** | **ns** |
| **Panel3_3040_Naive.B.cells** | **HIC_vs_non-HIC** | **0.212** | **0.122** | **ns** |
| **Panel2_2116_CD56+CD3+_PD1+** | **HIC_vs_non-HIC** | **-0.216** | **0.124** | **ns** |
| **Panel2_2338_CD8+.Tc2_HLA-DR+** | **HIC_vs_non-HIC** | **0.212** | **0.125** | **ns** |
| **Panel1_1027_DC** | **HIC_vs_non-HIC** | **0.213** | **0.125** | **ns** |
| **Panel2_2323_CD4+.Th17_HLA-DR+** | **HIC_vs_non-HIC** | **-0.209** | **0.131** | **ns** |
| **Panel1_1322_NK_CD56+CD16+_CCR5+** | **HIC_vs_non-HIC** | **-0.209** | **0.134** | **ns** |
| **Panel1_1186_mDC_CD86+** | **HIC_vs_non-HIC** | **-0.187** | **0.137** | **ns** |
| **Panel1_1123_TCRyd_HLA-DR+** | **HIC_vs_non-HIC** | **0.210** | **0.137** | **ns** |
| **Panel2_2322_CD4+.Th2_HLA-DR+** | **HIC_vs_non-HIC** | **-0.209** | **0.139** | **ns** |
| **Panel2_2348_CD56+CD3+_CCR4+** | **HIC_vs_non-HIC** | **0.186** | **0.140** | **ns** |
| **Panel1_1339_TCRyd_CCR5+** | **HIC_vs_non-HIC** | **-0.203** | **0.144** | **ns** |
| **Panel2_2082_CD8+PD1+** | **HIC_vs_non-HIC** | **-0.195** | **0.146** | **ns** |
| **Panel2_2258_CD4+.Tem_CD38+** | **HIC_vs_non-HIC** | **-0.178** | **0.153** | **ns** |
| **Panel2_2522_CD56+CD3+_CCR7+** | **HIC_vs_non-HIC** | **-0.194** | **0.153** | **ns** |
| **Panel2_2119_CD4+CD8+_PD1+** | **HIC_vs_non-HIC** | **-0.196** | **0.155** | **ns** |
| **Panel2_2719_CD4+.Tcm_CXCR5+** | **HIC_vs_non-HIC** | **-0.194** | **0.160** | **ns** |
| **Panel2_2043_CD4+.Tcm** | **HIC_vs_non-HIC** | **-0.189** | **0.165** | **ns** |
| **Panel3_3112_RM.(CD27+CD21+)_CD307d+** | **HIC_vs_non-HIC** | **-0.170** | **0.168** | **ns** |
| **Panel2_2646_Treg_CXCR4+** | **HIC_vs_non-HIC** | **0.156** | **0.169** | **ns** |
| **Panel2_2394_CD8+.Tem_CCR4+** | **HIC_vs_non-HIC** | **0.182** | **0.171** | **ns** |
| **Panel2_2589_mTreg_CXCR3+** | **HIC_vs_non-HIC** | **0.183** | **0.172** | **ns** |
| **Panel2_2264_CD4+.Th2_CD38+** | **HIC_vs_non-HIC** | **-0.172** | **0.173** | **ns** |
| **Panel2_2315_CD4+.Temra_HLA-DR+** | **HIC_vs_non-HIC** | **-0.185** | **0.174** | **ns** |
| **Panel2_2729_CD4+.Th17_CXCR5+** | **HIC_vs_non-HIC** | **-0.181** | **0.183** | **ns** |
| **Panel1_1035_NK_CD56+CD16+** | **HIC_vs_non-HIC** | **-0.187** | **0.183** | **ns** |
| **Panel3_3075_SMBC_IgG+IgA-** | **HIC_vs_non-HIC** | **-0.181** | **0.186** | **ns** |
| **Panel1_1015_Monocytes** | **HIC_vs_non-HIC** | **0.186** | **0.188** | **ns** |
| **Panel1_1013_Neutrophils** | **HIC_vs_non-HIC** | **0.182** | **0.188** | **ns** |
| **Panel2_2549_Tfh_1_CCR7+** | **HIC_vs_non-HIC** | **0.175** | **0.189** | **ns** |
| **Panel2_2293_CD4+CD8+_HLA-DR+** | **HIC_vs_non-HIC** | **0.182** | **0.195** | **ns** |
| **Panel3_3111_IM.(CD27-CD21+)_CD307d+** | **HIC_vs_non-HIC** | **-0.157** | **0.199** | **ns** |
| **Panel2_2431_CD4+.Temra_CCR5+** | **HIC_vs_non-HIC** | **-0.172** | **0.199** | **ns** |
| **Panel2_2259_Tfh_1_CD38+** | **HIC_vs_non-HIC** | **0.160** | **0.201** | **ns** |
| **Panel2_2463_NKcells_CCR6+** | **HIC_vs_non-HIC** | **0.152** | **0.208** | **ns** |
| **Panel2_2744_CD8+.Tc2_CXCR5+** | **HIC_vs_non-HIC** | **-0.175** | **0.208** | **ns** |
| **Panel2_2240_Treg_CD38+** | **HIC_vs_non-HIC** | **0.156** | **0.210** | **ns** |
| **Panel2_2555_CD4+.Th17_CCR7+** | **HIC_vs_non-HIC** | **0.176** | **0.211** | **ns** |
| **Panel1_1178_TCRvd1_CD86+** | **HIC_vs_non-HIC** | **-0.171** | **0.216** | **ns** |
| **Panel2_2728_CD4+.Th2_CXCR5+** | **HIC_vs_non-HIC** | **-0.167** | **0.217** | **ns** |
| **Panel2_2407_CD4+CD8-_CCR5+** | **HIC_vs_non-HIC** | **-0.165** | **0.221** | **ns** |
| **Panel2_2604_CD4+.Tnaive_CXCR3+** | **HIC_vs_non-HIC** | **-0.153** | **0.229** | **ns** |
| **Panel2_2162_CD8+.Tem_PD1+** | **HIC_vs_non-HIC** | **0.167** | **0.232** | **ns** |
| **Panel2_2013_CD56+CD3+** | **HIC_vs_non-HIC** | **0.167** | **0.232** | **ns** |
| **Panel2_2489_CD4+.Temra_CCR6+** | **HIC_vs_non-HIC** | **-0.164** | **0.238** | **ns** |
| **Panel1_1032_NK_CD56++CD16-** | **HIC_vs_non-HIC** | **0.160** | **0.240** | **ns** |
| **Panel1_1279_Bc_CXCR4+** | **HIC_vs_non-HIC** | **-0.148** | **0.240** | **ns** |
| **Panel2_2583_CD4+CD8+_CXCR3+** | **HIC_vs_non-HIC** | **0.163** | **0.246** | **ns** |
| **Panel2_2275_CD8+.Tcm_CD38+** | **HIC_vs_non-HIC** | **0.144** | **0.250** | **ns** |
| **Panel2_2429_CD4+.Tcm_CCR5+** | **HIC_vs_non-HIC** | **-0.151** | **0.253** | **ns** |
| **Panel2_2697_CD4+CD8-_CXCR5+** | **HIC_vs_non-HIC** | **-0.156** | **0.255** | **ns** |
| **Panel2_2160_CD8+.Tnaive_PD1+** | **HIC_vs_non-HIC** | **-0.151** | **0.259** | **ns** |
| **Panel1_1018_IM_CD14++CD16+** | **HIC_vs_non-HIC** | **0.155** | **0.262** | **ns** |
| **Panel2_2440_CD4+.Th1/17_CCR5+** | **HIC_vs_non-HIC** | **0.158** | **0.262** | **ns** |
| **Panel1_1049_TCRvd2** | **HIC_vs_non-HIC** | **0.157** | **0.263** | **ns** |
| **Panel2_2052_CD4+.Th2** | **HIC_vs_non-HIC** | **-0.152** | **0.264** | **ns** |
| **Panel2_2062_CD8+.Tnaive** | **HIC_vs_non-HIC** | **0.133** | **0.265** | **ns** |
| **Panel3_3096_Unswitched.Memory.B.cells_CD81+** | **HIC_vs_non-HIC** | **0.150** | **0.273** | **ns** |
| **Panel2_2696_CD56+CD3+_CXCR5+** | **HIC_vs_non-HIC** | **-0.148** | **0.280** | **ns** |
| **Panel3_3071_RM.(CD27+CD21+)** | **HIC_vs_non-HIC** | **0.149** | **0.281** | **ns** |
| **Panel2_2257_CD4+.Temra_CD38+** | **HIC_vs_non-HIC** | **-0.137** | **0.282** | **ns** |
| **Panel2_2241_mTreg_CD38+** | **HIC_vs_non-HIC** | **0.133** | **0.284** | **ns** |
| **Panel2_2095_CD4+CXCR4+CCR5-** | **HIC_vs_non-HIC** | **0.129** | **0.285** | **ns** |
| **Panel2_2730_CD4+.Th1/17_CXCR5+** | **HIC_vs_non-HIC** | **-0.144** | **0.286** | **ns** |
| **Panel2_2510_CD8+.Tem_CCR6+** | **HIC_vs_non-HIC** | **-0.143** | **0.289** | **ns** |
| **Panel1_1187_mDC_CD16-CD1c+_CD86+** | **HIC_vs_non-HIC** | **-0.124** | **0.289** | **ns** |
| **Panel2_2638_CD56+CD3+_CXCR4+** | **HIC_vs_non-HIC** | **-0.129** | **0.290** | **ns** |
| **Panel2_2686_CD8+.Tc2_CXCR4+** | **HIC_vs_non-HIC** | **-0.119** | **0.291** | **ns** |
| **Panel2_2582_CD4-CD8+_CXCR3+** | **HIC_vs_non-HIC** | **-0.143** | **0.296** | **ns** |
| **Panel2_2648_nTreg_CXCR4+** | **HIC_vs_non-HIC** | **0.126** | **0.300** | **ns** |
| **Panel1_1210_NK-T.like_PDL1+** | **HIC_vs_non-HIC** | **-0.113** | **0.302** | **ns** |
| **Panel2_2086_CD4+HLA-DR-CD38+** | **HIC_vs_non-HIC** | **0.123** | **0.304** | **ns** |
| **Panel2_2074_CD8+.Tc17** | **HIC_vs_non-HIC** | **-0.138** | **0.306** | **ns** |
| **Panel2_2688_CD8+.Tc1/17_CXCR4+** | **HIC_vs_non-HIC** | **-0.108** | **0.310** | **ns** |
| **Panel2_2473_mTreg_CCR6+** | **HIC_vs_non-HIC** | **-0.135** | **0.312** | **ns** |
| **Panel2_2487_CD4+.Tcm_CCR6+** | **HIC_vs_non-HIC** | **-0.140** | **0.316** | **ns** |
| **Panel2_2333_CD8+.Tcm_HLA-DR+** | **HIC_vs_non-HIC** | **0.140** | **0.317** | **ns** |
| **Panel2_2552_Tfh_1/17_CCR7+** | **HIC_vs_non-HIC** | **0.132** | **0.321** | **ns** |
| **Panel3_3017_Plasma.Cells** | **HIC_vs_non-HIC** | **-0.137** | **0.330** | **ns** |
| **Panel1_1202_IM_CD14++CD16+_PDL1+** | **HIC_vs_non-HIC** | **0.125** | **0.332** | **ns** |
| **Panel1_1016_CM_CD14++CD16-** | **HIC_vs_non-HIC** | **-0.134** | **0.333** | **ns** |
| **Panel1_1349_mDC_CD16-CD1c+_CCR5+** | **HIC_vs_non-HIC** | **-0.114** | **0.334** | **ns** |
| **Panel3_3078_SMBC_IgE-IgG+** | **HIC_vs_non-HIC** | **-0.133** | **0.335** | **ns** |
| **Panel1_1184_DC_CD86+** | **HIC_vs_non-HIC** | **-0.126** | **0.336** | **ns** |
| **Panel2_2277_CD8+.Temra_CD38+** | **HIC_vs_non-HIC** | **0.128** | **0.337** | **ns** |
| **Panel2_2262_Tfh_1/17_CD38+** | **HIC_vs_non-HIC** | **0.122** | **0.341** | **ns** |
| **Panel1_1031_NK_CD56-CD16+** | **HIC_vs_non-HIC** | **0.135** | **0.342** | **ns** |
| **Panel1_1228_Tc_CD4-CD8+_PDL1+** | **HIC_vs_non-HIC** | **-0.101** | **0.347** | **ns** |
| **Panel1_1019_NCM_CD14+CD16+** | **HIC_vs_non-HIC** | **0.127** | **0.352** | **ns** |
| **Panel1_1149_NCM_CD14+CD16+_CD86+** | **HIC_vs_non-HIC** | **0.127** | **0.356** | **ns** |
| **Panel2_2163_CD8+.Tc1_PD1+** | **HIC_vs_non-HIC** | **0.129** | **0.356** | **ns** |
| **Panel2_2324_CD4+.Th1/17_HLA-DR+** | **HIC_vs_non-HIC** | **-0.127** | **0.357** | **ns** |
| **Panel2_2722_CD4+.Tem_CXCR5+** | **HIC_vs_non-HIC** | **-0.125** | **0.357** | **ns** |
| **Panel2_2045_CD4+.Temra** | **HIC_vs_non-HIC** | **0.123** | **0.374** | **ns** |
| **Panel3_3114_Unswitched.Memory.B.cells_CD307d+** | **HIC_vs_non-HIC** | **-0.109** | **0.375** | **ns** |
| **Panel2_2261_Tfh_17_CD38+** | **HIC_vs_non-HIC** | **0.112** | **0.376** | **ns** |
| **Panel2_2166_CD8+.Tc1/17_PD1+** | **HIC_vs_non-HIC** | **0.120** | **0.382** | **ns** |
| **Panel2_2015_Tcells** | **HIC_vs_non-HIC** | **0.118** | **0.384** | **ns** |
| **Panel2_2551_Tfh_17_CCR7+** | **HIC_vs_non-HIC** | **0.113** | **0.391** | **ns** |
| **Panel2_2405_NKcells_CCR5+** | **HIC_vs_non-HIC** | **-0.114** | **0.395** | **ns** |
| **Panel2_2161_CD8+.Temra_PD1+** | **HIC_vs_non-HIC** | **-0.119** | **0.395** | **ns** |
| **Panel2_2115_NKcells_PD1+** | **HIC_vs_non-HIC** | **-0.117** | **0.399** | **ns** |
| **Panel2_2233_CD4+CD8-_CD38+** | **HIC_vs_non-HIC** | **0.100** | **0.402** | **ns** |
| **Panel2_2525_CD4+CD8+_CCR7+** | **HIC_vs_non-HIC** | **-0.115** | **0.404** | **ns** |
| **Panel2_2639_CD4+CD8-_CXCR4+** | **HIC_vs_non-HIC** | **0.097** | **0.404** | **ns** |
| **Panel2_2313_CD4+.Tcm_HLA-DR+** | **HIC_vs_non-HIC** | **-0.114** | **0.410** | **ns** |
| **Panel2_2681_CD8+.Tcm_CXCR4+** | **HIC_vs_non-HIC** | **-0.089** | **0.412** | **ns** |
| **Panel2_2637_NKcells_CXCR4+** | **HIC_vs_non-HIC** | **-0.101** | **0.412** | **ns** |
| **Panel1_1172_Tc_CD86+** | **HIC_vs_non-HIC** | **-0.111** | **0.415** | **ns** |
| **Panel3_3076_SMBC_IgG+IgA+** | **HIC_vs_non-HIC** | **-0.101** | **0.415** | **ns** |
| **Panel2_2150_CD4+.Th1/17_PD1+** | **HIC_vs_non-HIC** | **-0.110** | **0.423** | **ns** |
| **Panel1_1268_NK_CD56+CD16+_CXCR4+** | **HIC_vs_non-HIC** | **-0.102** | **0.424** | **ns** |
| **Panel2_2550_Tfh_2_CCR7+** | **HIC_vs_non-HIC** | **0.104** | **0.424** | **ns** |
| **Panel1_1047_TCRvd1** | **HIC_vs_non-HIC** | **-0.112** | **0.427** | **ns** |
| **Panel2_2490_CD4+.Tem_CCR6+** | **HIC_vs_non-HIC** | **0.108** | **0.429** | **ns** |
| **Panel2_2256_CD4+.Tnaive_CD38+** | **HIC_vs_non-HIC** | **-0.097** | **0.430** | **ns** |
| **Panel1_1294_mDC_CXCR4+** | **HIC_vs_non-HIC** | **0.098** | **0.435** | **ns** |
| **Panel2_2099_CD8+CXCR4+CCR5-** | **HIC_vs_non-HIC** | **0.092** | **0.449** | **ns** |
| **Panel2_2570_CD8+.Tc2_CCR7+** | **HIC_vs_non-HIC** | **0.102** | **0.453** | **ns** |
| **Panel2_2695_NKcells_CXCR5+** | **HIC_vs_non-HIC** | **-0.101** | **0.459** | **ns** |
| **Panel2_2580_CD56+CD3+_CXCR3+** | **HIC_vs_non-HIC** | **-0.101** | **0.460** | **ns** |
| **Panel2_2705_mTreg_CXCR5+** | **HIC_vs_non-HIC** | **-0.098** | **0.471** | **ns** |
| **Panel2_2663_CD4+.Temra_CXCR4+** | **HIC_vs_non-HIC** | **-0.083** | **0.472** | **ns** |
| **Panel2_2727_CD4+.Th1_CXCR5+** | **HIC_vs_non-HIC** | **-0.097** | **0.475** | **ns** |
| **Panel2_2641_CD4+CD8+_CXCR4+** | **HIC_vs_non-HIC** | **-0.086** | **0.476** | **ns** |
| **Panel1_1233_TCRvd2_PDL1+** | **HIC_vs_non-HIC** | **0.095** | **0.481** | **ns** |
| **Panel3_3116_Naive.B.cells_CD307d+** | **HIC_vs_non-HIC** | **-0.087** | **0.482** | **ns** |
| **Panel2_2579_NKcells_CXCR3+** | **HIC_vs_non-HIC** | **0.095** | **0.487** | **ns** |
| **Panel2_2393_CD8+.Temra_CCR4+** | **HIC_vs_non-HIC** | **0.088** | **0.491** | **ns** |
| **Panel2_2148_CD4+.Th2_PD1+** | **HIC_vs_non-HIC** | **-0.083** | **0.494** | **ns** |
| **Panel1_1346_DC_CCR5+** | **HIC_vs_non-HIC** | **-0.088** | **0.494** | **ns** |
| **Panel2_2669_CD4+.Th1_CXCR4+** | **HIC_vs_non-HIC** | **-0.079** | **0.494** | **ns** |
| **Panel2_2164_CD8+.Tc2_PD1+** | **HIC_vs_non-HIC** | **0.090** | **0.495** | **ns** |
| **Panel2_2334_CD8+.Tnaive_HLA-DR+** | **HIC_vs_non-HIC** | **0.094** | **0.495** | **ns** |
| **Panel1_1311_NCM_CD14+CD16+_CCR5+** | **HIC_vs_non-HIC** | **0.086** | **0.499** | **ns** |
| **Panel2_2019_CD4+CD8+** | **HIC_vs_non-HIC** | **0.093** | **0.500** | **ns** |
| **Panel2_2437_CD4+.Th1_CCR5+** | **HIC_vs_non-HIC** | **0.090** | **0.515** | **ns** |
| **Panel2_2096_CD4+CXCR4+CCR5+** | **HIC_vs_non-HIC** | **-0.078** | **0.517** | **ns** |
| **Panel2_2090_CD8+HLA-DR-CD38+** | **HIC_vs_non-HIC** | **0.078** | **0.528** | **ns** |
| **Panel1_1045_TCRyd** | **HIC_vs_non-HIC** | **-0.088** | **0.536** | **ns** |
| **Panel2_2350_CD4-CD8+_CCR4+** | **HIC_vs_non-HIC** | **0.079** | **0.541** | **ns** |
| **Panel1_1266_NK_CD56++CD16+_CXCR4+** | **HIC_vs_non-HIC** | **-0.082** | **0.543** | **ns** |
| **Panel2_2314_CD4+.Tnaive_HLA-DR+** | **HIC_vs_non-HIC** | **0.081** | **0.547** | **ns** |
| **Panel1_1295_mDC_CD16-CD1c+_CXCR4+** | **HIC_vs_non-HIC** | **0.071** | **0.548** | **ns** |
| **Panel1_1033_NK_CD56++CD16+** | **HIC_vs_non-HIC** | **0.084** | **0.551** | **ns** |
| **Panel2_2682_CD8+.Tnaive_CXCR4+** | **HIC_vs_non-HIC** | **-0.070** | **0.555** | **ns** |
| **Panel3_3069_AM.(CD27+CD21-)** | **HIC_vs_non-HIC** | **0.083** | **0.555** | **ns** |
| **Panel1_1348_mDC_CCR5+** | **HIC_vs_non-HIC** | **-0.074** | **0.567** | **ns** |
| **Panel3_3039_Immature.B.cells** | **HIC_vs_non-HIC** | **0.078** | **0.573** | **ns** |
| **Panel2_2666_Tfh_2_CXCR4+** | **HIC_vs_non-HIC** | **-0.057** | **0.598** | **ns** |
| **Panel1_1269_NK_CD56-CD16+_CXCR4+** | **HIC_vs_non-HIC** | **0.063** | **0.601** | **ns** |
| **Panel1_1125_TCRvd2_HLA-DR+** | **HIC_vs_non-HIC** | **0.070** | **0.608** | **ns** |
| **Panel1_1028_Basophils** | **HIC_vs_non-HIC** | **-0.072** | **0.615** | **ns** |
| **Panel2_2276_CD8+.Tnaive_CD38+** | **HIC_vs_non-HIC** | **0.062** | **0.621** | **ns** |
| **Panel2_2051_CD4+.Th1** | **HIC_vs_non-HIC** | **0.068** | **0.622** | **ns** |
| **Panel2_2672_CD4+.Th1/17_CXCR4+** | **HIC_vs_non-HIC** | **-0.053** | **0.625** | **ns** |
| **Panel2_2321_CD4+.Th1_HLA-DR+** | **HIC_vs_non-HIC** | **0.068** | **0.626** | **ns** |
| **Panel3_3094_Plasma.Cells_CD81+** | **HIC_vs_non-HIC** | **-0.053** | **0.634** | **ns** |
| **Panel2_2698_CD4-CD8+_CXCR5+** | **HIC_vs_non-HIC** | **-0.065** | **0.635** | **ns** |
| **Panel2_2670_CD4+.Th2_CXCR4+** | **HIC_vs_non-HIC** | **0.052** | **0.637** | **ns** |
| **Panel1_1171_Bc_CD86+** | **HIC_vs_non-HIC** | **-0.053** | **0.638** | **ns** |
| **Panel1_1065_Bc_CD40+** | **HIC_vs_non-HIC** | **-0.066** | **0.639** | **ns** |
| **Panel2_2265_CD4+.Th17_CD38+** | **HIC_vs_non-HIC** | **-0.058** | **0.640** | **ns** |
| **Panel1_1225_Bc_PDL1+** | **HIC_vs_non-HIC** | **0.060** | **0.644** | **ns** |
| **Panel2_2647_mTreg_CXCR4+** | **HIC_vs_non-HIC** | **0.051** | **0.646** | **ns** |
| **Panel3_3095_Switched.Memory.B.cells_CD81+** | **HIC_vs_non-HIC** | **-0.062** | **0.657** | **ns** |
| **Panel2_2742_CD8+.Tem_CXCR5+** | **HIC_vs_non-HIC** | **-0.059** | **0.670** | **ns** |
| **Panel2_2720_CD4+.Tnaive_CXCR5+** | **HIC_vs_non-HIC** | **-0.057** | **0.682** | **ns** |
| **Panel2_2704_Treg_CXCR5+** | **HIC_vs_non-HIC** | **-0.055** | **0.684** | **ns** |
| **Panel1_1255_CM_CD14++CD16-_CXCR4+** | **HIC_vs_non-HIC** | **0.053** | **0.688** | **ns** |
| **Panel2_2070_CD8+.Tc2** | **HIC_vs_non-HIC** | **0.054** | **0.691** | **ns** |
| **Panel2_2668_Tfh_1/17_CXCR4+** | **HIC_vs_non-HIC** | **-0.043** | **0.693** | **ns** |
| **Panel2_2242_nTreg_CD38+** | **HIC_vs_non-HIC** | **0.049** | **0.698** | **ns** |
| **Panel2_2464_CD56+CD3+_CCR6+** | **HIC_vs_non-HIC** | **0.049** | **0.710** | **ns** |
| **Panel2_2290_CD56+CD3+_HLA-DR+** | **HIC_vs_non-HIC** | **0.052** | **0.711** | **ns** |
| **Panel2_2392_CD8+.Tnaive_CCR4+** | **HIC_vs_non-HIC** | **0.049** | **0.716** | **ns** |
| **Panel2_2623_CD8+.Tcm_CXCR3+** | **HIC_vs_non-HIC** | **-0.049** | **0.719** | **ns** |
| **Panel2_2524_CD4-CD8+_CCR7+** | **HIC_vs_non-HIC** | **0.045** | **0.721** | **ns** |
| **Panel1_1248_Eosinophils_CXCR4+** | **HIC_vs_non-HIC** | **-0.045** | **0.723** | **ns** |
| **Panel2_2347_NKcells_CCR4+** | **HIC_vs_non-HIC** | **0.048** | **0.723** | **ns** |
| **Panel2_2553_CD4+.Th1_CCR7+** | **HIC_vs_non-HIC** | **-0.049** | **0.725** | **ns** |
| **Panel2_2665_Tfh_1_CXCR4+** | **HIC_vs_non-HIC** | **-0.038** | **0.727** | **ns** |
| **Panel2_2683_CD8+.Temra_CXCR4+** | **HIC_vs_non-HIC** | **-0.039** | **0.728** | **ns** |
| **Panel1_1310_IM_CD14++CD16+_CCR5+** | **HIC_vs_non-HIC** | **0.042** | **0.733** | **ns** |
| **Panel2_2263_CD4+.Th1_CD38+** | **HIC_vs_non-HIC** | **-0.043** | **0.734** | **ns** |
| **Panel2_2316_CD4+.Tem_HLA-DR+** | **HIC_vs_non-HIC** | **-0.046** | **0.739** | **ns** |
| **Panel1_1174_Tc_CD4-CD8+_CD86+** | **HIC_vs_non-HIC** | **-0.046** | **0.741** | **ns** |
| **Panel1_1256_IM_CD14++CD16+_CXCR4+** | **HIC_vs_non-HIC** | **-0.044** | **0.741** | **ns** |
| **Panel2_2432_CD4+.Tem_CCR5+** | **HIC_vs_non-HIC** | **0.044** | **0.744** | **ns** |
| **Panel2_2721_CD4+.Temra_CXCR5+** | **HIC_vs_non-HIC** | **-0.045** | **0.745** | **ns** |
| **Panel1_1249_Basophils_CXCR4+** | **HIC_vs_non-HIC** | **-0.035** | **0.746** | **ns** |
| **Panel2_2372_CD4+.Tnaive_CCR4+** | **HIC_vs_non-HIC** | **0.044** | **0.752** | **ns** |
| **Panel2_2572_CD8+.Tc1/17_CCR7+** | **HIC_vs_non-HIC** | **0.043** | **0.755** | **ns** |
| **Panel1_1301_Neutrophils_CCR5+** | **HIC_vs_non-HIC** | **0.040** | **0.763** | **ns** |
| **Panel1_1286_TCRvd1_CXCR4+** | **HIC_vs_non-HIC** | **-0.037** | **0.764** | **ns** |
| **Panel2_2260_Tfh_2_CD38+** | **HIC_vs_non-HIC** | **-0.039** | **0.766** | **ns** |
| **Panel2_2289_NKcells_HLA-DR+** | **HIC_vs_non-HIC** | **0.040** | **0.770** | **ns** |
| **Panel2_2149_CD4+.Th17_PD1+** | **HIC_vs_non-HIC** | **-0.035** | **0.778** | **ns** |
| **Panel2_2626_CD8+.Tem_CXCR3+** | **HIC_vs_non-HIC** | **-0.037** | **0.790** | **ns** |
| **Panel2_2454_CD8+.Tc2_CCR5+** | **HIC_vs_non-HIC** | **-0.034** | **0.795** | **ns** |
| **Panel2_2662_CD4+.Tnaive_CXCR4+** | **HIC_vs_non-HIC** | **-0.028** | **0.801** | **ns** |
| **Panel1_1071_Bc_CD86+PDL1+** | **HIC_vs_non-HIC** | **-0.029** | **0.805** | **ns** |
| **Panel1_1201_CM_CD14++CD16-_PDL1+** | **HIC_vs_non-HIC** | **0.029** | **0.810** | **ns** |
| **Panel1_1238_DC_PDL1+** | **HIC_vs_non-HIC** | **0.028** | **0.814** | **ns** |
| **Panel3_3074_SMBC_IgG-IgA+** | **HIC_vs_non-HIC** | **0.031** | **0.819** | **ns** |
| **Panel2_2699_CD4+CD8+_CXCR5+** | **HIC_vs_non-HIC** | **0.031** | **0.819** | **ns** |
| **Panel2_2664_CD4+.Tem_CXCR4+** | **HIC_vs_non-HIC** | **-0.023** | **0.820** | **ns** |
| **Panel2_2235_CD4+CD8+_CD38+** | **HIC_vs_non-HIC** | **-0.027** | **0.823** | **ns** |
| **Panel2_2409_CD4+CD8+_CCR5+** | **HIC_vs_non-HIC** | **0.030** | **0.826** | **ns** |
| **Panel2_2624_CD8+.Tnaive_CXCR3+** | **HIC_vs_non-HIC** | **0.031** | **0.826** | **ns** |
| **Panel2_2488_CD4+.Tnaive_CCR6+** | **HIC_vs_non-HIC** | **-0.027** | **0.834** | **ns** |
| **Panel2_2232_CD56+CD3+_CD38+** | **HIC_vs_non-HIC** | **0.026** | **0.837** | **ns** |
| **Panel1_1287_TCRvd2_CXCR4+** | **HIC_vs_non-HIC** | **-0.024** | **0.842** | **ns** |
| **Panel1_1309_CM_CD14++CD16-_CCR5+** | **HIC_vs_non-HIC** | **0.023** | **0.846** | **ns** |
| **Panel1_1038_mDC** | **HIC_vs_non-HIC** | **0.023** | **0.858** | **ns** |
| **Panel2_2255_CD4+.Tcm_CD38+** | **HIC_vs_non-HIC** | **0.022** | **0.860** | **ns** |
| **Panel3_3028_Unswitched.Memory.B.cells** | **HIC_vs_non-HIC** | **0.025** | **0.861** | **ns** |
| **Panel2_2743_CD8+.Tc1_CXCR5+** | **HIC_vs_non-HIC** | **-0.023** | **0.864** | **ns** |
| **Panel2_2667_Tfh_17_CXCR4+** | **HIC_vs_non-HIC** | **0.019** | **0.866** | **ns** |
| **Panel2_2147_CD4+.Th1_PD1+** | **HIC_vs_non-HIC** | **-0.023** | **0.867** | **ns** |
| **Panel2_2521_NKcells_CCR7+** | **HIC_vs_non-HIC** | **-0.023** | **0.870** | **ns** |
| **Panel2_2340_CD8+.Tc1/17_HLA-DR+** | **HIC_vs_non-HIC** | **-0.020** | **0.882** | **ns** |
| **Panel1_1177_TCRyd_CD86+** | **HIC_vs_non-HIC** | **0.020** | **0.888** | **ns** |
| **Panel1_1041_mDC_CD16-CD1c+** | **HIC_vs_non-HIC** | **0.017** | **0.898** | **ns** |
| **Panel2_2685_CD8+.Tc1_CXCR4+** | **HIC_vs_non-HIC** | **-0.014** | **0.900** | **ns** |
| **Panel1_1285_TCRyd_CXCR4+** | **HIC_vs_non-HIC** | **0.015** | **0.903** | **ns** |
| **Panel1_1257_NCM_CD14+CD16+_CXCR4+** | **HIC_vs_non-HIC** | **0.015** | **0.906** | **ns** |
| **Panel1_1069_Bc_CD86-PDL1+** | **HIC_vs_non-HIC** | **0.015** | **0.909** | **ns** |
| **Panel2_2159_CD8+.Tcm_PD1+** | **HIC_vs_non-HIC** | **-0.015** | **0.910** | **ns** |
| **Panel2_2069_CD8+.Tc1** | **HIC_vs_non-HIC** | **-0.015** | **0.913** | **ns** |
| **Panel1_1070_Bc_CD86+PDL1-** | **HIC_vs_non-HIC** | **0.013** | **0.916** | **ns** |
| **Panel2_2671_CD4+.Th17_CXCR4+** | **HIC_vs_non-HIC** | **-0.011** | **0.922** | **ns** |
| **Panel2_2706_nTreg_CXCR5+** | **HIC_vs_non-HIC** | **0.013** | **0.926** | **ns** |
| **Panel1_1247_Neutrophils_CXCR4+** | **HIC_vs_non-HIC** | **-0.012** | **0.930** | **ns** |
| **Panel1_1302_Eosinophils_CCR5+** | **HIC_vs_non-HIC** | **0.010** | **0.938** | **ns** |
| **Panel2_2509_CD8+.Temra_CCR6+** | **HIC_vs_non-HIC** | **0.010** | **0.939** | **ns** |
| **Panel1_1124_TCRvd1_HLA-DR+** | **HIC_vs_non-HIC** | **-0.010** | **0.942** | **ns** |
| **Panel1_1179_TCRvd2_CD86+** | **HIC_vs_non-HIC** | **-0.010** | **0.944** | **ns** |
| **Panel2_2625_CD8+.Temra_CXCR3+** | **HIC_vs_non-HIC** | **-0.010** | **0.944** | **ns** |
| **Panel2_2684_CD8+.Tem_CXCR4+** | **HIC_vs_non-HIC** | **0.006** | **0.954** | **ns** |
| **Panel2_2231_NKcells_CD38+** | **HIC_vs_non-HIC** | **-0.007** | **0.960** | **ns** |
| **Panel1_1292_DC_CXCR4+** | **HIC_vs_non-HIC** | **-0.005** | **0.965** | **ns** |
| **Panel3_3082_SMBC_IgE-IgA+** | **HIC_vs_non-HIC** | **-0.005** | **0.968** | **ns** |
| **Panel2_2351_CD4+CD8+_CCR4+** | **HIC_vs_non-HIC** | **-0.005** | **0.969** | **ns** |
| **Panel1_1034_NK_CD56+CD16-** | **HIC_vs_non-HIC** | **-0.005** | **0.969** | **ns** |
| **Panel2_2569_CD8+.Tc1_CCR7+** | **HIC_vs_non-HIC** | **0.005** | **0.970** | **ns** |
| **Panel2_2588_Treg_CXCR3+** | **HIC_vs_non-HIC** | **-0.005** | **0.972** | **ns** |
| **Panel2_2605_CD4+.Temra_CXCR3+** | **HIC_vs_non-HIC** | **0.005** | **0.972** | **ns** |
| **Panel1_1231_TCRyd_PDL1+** | **HIC_vs_non-HIC** | **0.004** | **0.972** | **ns** |
| **Panel2_2640_CD4-CD8+_CXCR4+** | **HIC_vs_non-HIC** | **-0.004** | **0.975** | **ns** |
| **Panel3_3072_TLM.(CD27-CD21-)** | **HIC_vs_non-HIC** | **-0.004** | **0.979** | **ns** |
| **Panel2_2391_CD8+.Tcm_CCR4+** | **HIC_vs_non-HIC** | **0.003** | **0.980** | **ns** |
| **Panel2_2741_CD8+.Temra_CXCR5+** | **HIC_vs_non-HIC** | **-0.003** | **0.982** | **ns** |
| **Panel2_2661_CD4+.Tcm_CXCR4+** | **HIC_vs_non-HIC** | **0.002** | **0.985** | **ns** |
| **Panel3_3010_CD19+** | **HIC_vs_non-HIC** | **0.001** | **0.992** | **ns** |
| **Panel2_2280_CD8+.Tc2_CD38+** | **HIC_vs_non-HIC** | **-0.001** | **0.995** | **ns** |
| **Panel2_2508_CD8+.Tnaive_CCR6+** | **HIC_vs_non-HIC** | **0.001** | **0.996** | **ns** |

**Supplementary TABLE 7. Summary statistics from linear regression model comparing EC vs non-HIC**

| Cell subset | Comparison | Estimate | P-value | Stars |
| --- | --- | --- | --- | --- |
| **Panel1_1049_TCRvd2** | **EC_vs_non-HIC** | **0.897** | **<0.001** | ****** |
| **Panel2_2017_CD4-CD8+** | **EC_vs_non-HIC** | **-0.787** | **<0.001** | ***** |
| **Panel2_2018_CD4+CD8-** | **EC_vs_non-HIC** | **0.758** | **<0.001** | ***** |
| **Panel2_2055_CD4+.Th1/17** | **EC_vs_non-HIC** | **0.727** | **<0.001** | ***** |
| **Panel2_2349_CD4+CD8-_CCR4+** | **EC_vs_non-HIC** | **-0.680** | **0.002** | ***** |
| **Panel2_2374_CD4+.Tem_CCR4+** | **EC_vs_non-HIC** | **-0.622** | **0.005** | ***** |
| **Panel2_2507_CD8+.Tcm_CCR6+** | **EC_vs_non-HIC** | **-0.601** | **0.005** | ***** |
| **Panel2_2264_CD4+.Th2_CD38+** | **EC_vs_non-HIC** | **-0.555** | **0.005** | ***** |
| **Panel2_2371_CD4+.Tcm_CCR4+** | **EC_vs_non-HIC** | **-0.567** | **0.010** | ***** |
| **Panel2_2088_CD4+HLA-DR+CD38+** | **EC_vs_non-HIC** | **-0.525** | **0.013** | ***** |
| **Panel2_2337_CD8+.Tc1_HLA-DR+** | **EC_vs_non-HIC** | **0.548** | **0.013** | ***** |
| **Panel2_2440_CD4+.Th1/17_CCR5+** | **EC_vs_non-HIC** | **0.535** | **0.016** | ***** |
| **Panel2_2487_CD4+.Tcm_CCR6+** | **EC_vs_non-HIC** | **-0.524** | **0.017** | ***** |
| **Panel2_2078_CD4+PD1+** | **EC_vs_non-HIC** | **-0.500** | **0.017** | ***** |
| **Panel2_2142_CD4+.Tem_PD1+** | **EC_vs_non-HIC** | **-0.500** | **0.018** | ***** |
| **Panel2_2291_CD4+CD8-_HLA-DR+** | **EC_vs_non-HIC** | **-0.504** | **0.019** | ***** |
| **Panel2_2603_CD4+.Tcm_CXCR3+** | **EC_vs_non-HIC** | **-0.505** | **0.020** | ***** |
| **Panel2_2554_CD4+.Th2_CCR7+** | **EC_vs_non-HIC** | **0.507** | **0.022** | ***** |
| **Panel1_1047_TCRvd1** | **EC_vs_non-HIC** | **-0.498** | **0.026** | ***** |
| **Panel3_3116_Naive.B.cells_CD307d+** | **EC_vs_non-HIC** | **-0.426** | **0.027** | ***** |
| **Panel2_2467_CD4+CD8+_CCR6+** | **EC_vs_non-HIC** | **0.461** | **0.030** | ***** |
| **Panel3_3111_IM.(CD27-CD21+)_CD307d+** | **EC_vs_non-HIC** | **-0.410** | **0.030** | ***** |
| **Panel1_1125_TCRvd2_HLA-DR+** | **EC_vs_non-HIC** | **-0.461** | **0.034** | ***** |
| **Panel3_3114_Unswitched.Memory.B.cells_CD307d+** | **EC_vs_non-HIC** | **-0.403** | **0.035** | ***** |
| **Panel2_2530_Treg_CCR7+** | **EC_vs_non-HIC** | **0.447** | **0.036** | ***** |
| **Panel2_2336_CD8+.Tem_HLA-DR+** | **EC_vs_non-HIC** | **0.462** | **0.036** | ***** |
| **Panel2_2087_CD4+HLA-DR+CD38-** | **EC_vs_non-HIC** | **-0.415** | **0.037** | ***** |
| **Panel2_2322_CD4+.Th2_HLA-DR+** | **EC_vs_non-HIC** | **-0.463** | **0.037** | ***** |
| **Panel1_1266_NK_CD56++CD16+_CXCR4+** | **EC_vs_non-HIC** | **0.447** | **0.039** | ***** |
| **Panel2_2056_CD4+.Th17** | **EC_vs_non-HIC** | **-0.429** | **0.042** | ***** |
| **Panel2_2289_NKcells_HLA-DR+** | **EC_vs_non-HIC** | **-0.434** | **0.044** | ***** |
| **Panel3_3075_SMBC_IgG+IgA-** | **EC_vs_non-HIC** | **-0.431** | **0.044** | ***** |
| **Panel1_1012_Eosinophils** | **EC_vs_non-HIC** | **-0.447** | **0.046** | ***** |
| **Panel1_1013_Neutrophils** | **EC_vs_non-HIC** | **0.439** | **0.046** | ***** |
| **Panel2_2324_CD4+.Th1/17_HLA-DR+** | **EC_vs_non-HIC** | **-0.425** | **0.050** | ***** |
| **Panel1_1179_TCRvd2_CD86+** | **EC_vs_non-HIC** | **-0.421** | **0.050** | **ns** |
| **Panel3_3109_Switched.Memory.B.cells_CD307d+** | **EC_vs_non-HIC** | **-0.373** | **0.052** | **ns** |
| **Panel1_1174_Tc_CD4-CD8+_CD86+** | **EC_vs_non-HIC** | **-0.426** | **0.053** | **ns** |
| **Panel2_2623_CD8+.Tcm_CXCR3+** | **EC_vs_non-HIC** | **-0.416** | **0.054** | **ns** |
| **Panel3_3078_SMBC_IgE-IgG+** | **EC_vs_non-HIC** | **-0.413** | **0.057** | **ns** |
| **Panel2_2581_CD4+CD8-_CXCR3+** | **EC_vs_non-HIC** | **-0.397** | **0.059** | **ns** |
| **Panel2_2279_CD8+.Tc1_CD38+** | **EC_vs_non-HIC** | **0.380** | **0.062** | **ns** |
| **Panel1_1172_Tc_CD86+** | **EC_vs_non-HIC** | **-0.404** | **0.063** | **ns** |
| **Panel3_3017_Plasma.Cells** | **EC_vs_non-HIC** | **-0.411** | **0.064** | **ns** |
| **Panel2_2060_CD8+.Tem** | **EC_vs_non-HIC** | **-0.385** | **0.065** | **ns** |
| **Panel2_2046_CD4+.Tnaive** | **EC_vs_non-HIC** | **0.383** | **0.066** | **ns** |
| **Panel1_1173_Tc_CD4+CD8-_CD86+** | **EC_vs_non-HIC** | **-0.390** | **0.069** | **ns** |
| **Panel2_2523_CD4+CD8-_CCR7+** | **EC_vs_non-HIC** | **0.388** | **0.071** | **ns** |
| **Panel2_2140_CD4+.Tnaive_PD1+** | **EC_vs_non-HIC** | **-0.384** | **0.072** | **ns** |
| **Panel2_2531_mTreg_CCR7+** | **EC_vs_non-HIC** | **0.394** | **0.072** | **ns** |
| **Panel2_2030_Tfh** | **EC_vs_non-HIC** | **-0.389** | **0.074** | **ns** |
| **Panel2_2316_CD4+.Tem_HLA-DR+** | **EC_vs_non-HIC** | **-0.390** | **0.074** | **ns** |
| **Panel2_2532_nTreg_CCR7+** | **EC_vs_non-HIC** | **0.376** | **0.076** | **ns** |
| **Panel2_2323_CD4+.Th17_HLA-DR+** | **EC_vs_non-HIC** | **-0.383** | **0.079** | **ns** |
| **Panel2_2096_CD4+CXCR4+CCR5+** | **EC_vs_non-HIC** | **0.332** | **0.082** | **ns** |
| **Panel1_1227_Tc_CD4+CD8-_PDL1+** | **EC_vs_non-HIC** | **-0.328** | **0.083** | **ns** |
| **Panel2_2570_CD8+.Tc2_CCR7+** | **EC_vs_non-HIC** | **0.367** | **0.086** | **ns** |
| **Panel2_2166_CD8+.Tc1/17_PD1+** | **EC_vs_non-HIC** | **0.365** | **0.090** | **ns** |
| **Panel2_2139_CD4+.Tcm_PD1+** | **EC_vs_non-HIC** | **-0.355** | **0.091** | **ns** |
| **Panel2_2290_CD56+CD3+_HLA-DR+** | **EC_vs_non-HIC** | **-0.370** | **0.092** | **ns** |
| **Panel2_2298_Treg_HLA-DR+** | **EC_vs_non-HIC** | **-0.345** | **0.093** | **ns** |
| **Panel2_2039_Tfh_1/17** | **EC_vs_non-HIC** | **0.351** | **0.096** | **ns** |
| **Panel1_1226_Tc_PDL1+** | **EC_vs_non-HIC** | **-0.302** | **0.096** | **ns** |
| **Panel1_1233_TCRvd2_PDL1+** | **EC_vs_non-HIC** | **-0.355** | **0.096** | **ns** |
| **Panel2_2358_nTreg_CCR4+** | **EC_vs_non-HIC** | **-0.332** | **0.097** | **ns** |
| **Panel1_1018_IM_CD14++CD16+** | **EC_vs_non-HIC** | **0.363** | **0.099** | **ns** |
| **Panel2_2556_CD4+.Th1/17_CCR7+** | **EC_vs_non-HIC** | **-0.359** | **0.099** | **ns** |
| **Panel2_2115_NKcells_PD1+** | **EC_vs_non-HIC** | **-0.356** | **0.102** | **ns** |
| **Panel2_2141_CD4+.Temra_PD1+** | **EC_vs_non-HIC** | **-0.347** | **0.103** | **ns** |
| **Panel2_2092_CD8+HLA-DR+CD38+** | **EC_vs_non-HIC** | **0.354** | **0.105** | **ns** |
| **Panel1_1269_NK_CD56-CD16+_CXCR4+** | **EC_vs_non-HIC** | **0.306** | **0.109** | **ns** |
| **Panel2_2299_mTreg_HLA-DR+** | **EC_vs_non-HIC** | **-0.340** | **0.109** | **ns** |
| **Panel1_1193_Neutrophils_PDL1+** | **EC_vs_non-HIC** | **-0.290** | **0.114** | **ns** |
| **Panel2_2356_Treg_CCR4+** | **EC_vs_non-HIC** | **-0.311** | **0.115** | **ns** |
| **Panel2_2258_CD4+.Tem_CD38+** | **EC_vs_non-HIC** | **-0.306** | **0.116** | **ns** |
| **Panel2_2095_CD4+CXCR4+CCR5-** | **EC_vs_non-HIC** | **0.297** | **0.118** | **ns** |
| **Panel2_2432_CD4+.Tem_CCR5+** | **EC_vs_non-HIC** | **0.328** | **0.121** | **ns** |
| **Panel1_1340_TCRvd1_CCR5+** | **EC_vs_non-HIC** | **-0.334** | **0.123** | **ns** |
| **Panel1_1339_TCRyd_CCR5+** | **EC_vs_non-HIC** | **0.340** | **0.124** | **ns** |
| **Panel2_2639_CD4+CD8-_CXCR4+** | **EC_vs_non-HIC** | **0.280** | **0.127** | **ns** |
| **Panel2_2061_CD8+.Temra** | **EC_vs_non-HIC** | **0.330** | **0.130** | **ns** |
| **Panel3_3112_RM.(CD27+CD21+)_CD307d+** | **EC_vs_non-HIC** | **-0.285** | **0.136** | **ns** |
| **Panel3_3097_Immature.B.cells_CD81+** | **EC_vs_non-HIC** | **0.272** | **0.139** | **ns** |
| **Panel1_1228_Tc_CD4-CD8+_PDL1+** | **EC_vs_non-HIC** | **-0.249** | **0.144** | **ns** |
| **Panel3_3039_Immature.B.cells** | **EC_vs_non-HIC** | **0.318** | **0.144** | **ns** |
| **Panel2_2347_NKcells_CCR4+** | **EC_vs_non-HIC** | **-0.307** | **0.147** | **ns** |
| **Panel2_2266_CD4+.Th1/17_CD38+** | **EC_vs_non-HIC** | **-0.279** | **0.149** | **ns** |
| **Panel2_2024_mTreg** | **EC_vs_non-HIC** | **-0.288** | **0.150** | **ns** |
| **Panel2_2025_nTreg** | **EC_vs_non-HIC** | **0.288** | **0.150** | **ns** |
| **Panel1_1016_CM_CD14++CD16-** | **EC_vs_non-HIC** | **-0.316** | **0.151** | **ns** |
| **Panel2_2350_CD4-CD8+_CCR4+** | **EC_vs_non-HIC** | **-0.292** | **0.152** | **ns** |
| **Panel2_2124_Treg_PD1+** | **EC_vs_non-HIC** | **-0.279** | **0.153** | **ns** |
| **Panel2_2670_CD4+.Th2_CXCR4+** | **EC_vs_non-HIC** | **0.243** | **0.158** | **ns** |
| **Panel2_2746_CD8+.Tc1/17_CXCR5+** | **EC_vs_non-HIC** | **-0.301** | **0.160** | **ns** |
| **Panel2_2582_CD4-CD8+_CXCR3+** | **EC_vs_non-HIC** | **-0.288** | **0.182** | **ns** |
| **Panel2_2035_Tfh_1** | **EC_vs_non-HIC** | **0.284** | **0.184** | **ns** |
| **Panel2_2685_CD8+.Tc1_CXCR4+** | **EC_vs_non-HIC** | **0.233** | **0.184** | **ns** |
| **Panel3_3098_Naive.B.cells_CD81+** | **EC_vs_non-HIC** | **0.286** | **0.189** | **ns** |
| **Panel2_2313_CD4+.Tcm_HLA-DR+** | **EC_vs_non-HIC** | **-0.285** | **0.190** | **ns** |
| **Panel2_2292_CD4-CD8+_HLA-DR+** | **EC_vs_non-HIC** | **0.283** | **0.191** | **ns** |
| **Panel2_2321_CD4+.Th1_HLA-DR+** | **EC_vs_non-HIC** | **-0.287** | **0.191** | **ns** |
| **Panel2_2488_CD4+.Tnaive_CCR6+** | **EC_vs_non-HIC** | **-0.264** | **0.192** | **ns** |
| **Panel3_3074_SMBC_IgG-IgA+** | **EC_vs_non-HIC** | **0.279** | **0.193** | **ns** |
| **Panel2_2265_CD4+.Th17_CD38+** | **EC_vs_non-HIC** | **-0.253** | **0.193** | **ns** |
| **Panel2_2164_CD8+.Tc2_PD1+** | **EC_vs_non-HIC** | **0.270** | **0.193** | **ns** |
| **Panel2_2062_CD8+.Tnaive** | **EC_vs_non-HIC** | **0.243** | **0.194** | **ns** |
| **Panel2_2098_CD8+CXCR4-CCR5+** | **EC_vs_non-HIC** | **-0.259** | **0.194** | **ns** |
| **Panel1_1149_NCM_CD14+CD16+_CD86+** | **EC_vs_non-HIC** | **0.284** | **0.196** | **ns** |
| **Panel2_2099_CD8+CXCR4+CCR5-** | **EC_vs_non-HIC** | **0.245** | **0.200** | **ns** |
| **Panel2_2073_CD8+.Tc1/17** | **EC_vs_non-HIC** | **-0.277** | **0.203** | **ns** |
| **Panel2_2280_CD8+.Tc2_CD38+** | **EC_vs_non-HIC** | **-0.269** | **0.205** | **ns** |
| **Panel1_1124_TCRvd1_HLA-DR+** | **EC_vs_non-HIC** | **-0.281** | **0.210** | **ns** |
| **Panel2_2555_CD4+.Th17_CCR7+** | **EC_vs_non-HIC** | **0.276** | **0.212** | **ns** |
| **Panel3_3040_Naive.B.cells** | **EC_vs_non-HIC** | **0.261** | **0.226** | **ns** |
| **Panel2_2696_CD56+CD3+_CXCR5+** | **EC_vs_non-HIC** | **-0.262** | **0.226** | **ns** |
| **Panel2_2684_CD8+.Tem_CXCR4+** | **EC_vs_non-HIC** | **0.205** | **0.227** | **ns** |
| **Panel2_2278_CD8+.Tem_CD38+** | **EC_vs_non-HIC** | **0.251** | **0.229** | **ns** |
| **Panel2_2640_CD4-CD8+_CXCR4+** | **EC_vs_non-HIC** | **0.222** | **0.230** | **ns** |
| **Panel2_2510_CD8+.Tem_CCR6+** | **EC_vs_non-HIC** | **-0.254** | **0.231** | **ns** |
| **Panel2_2393_CD8+.Temra_CCR4+** | **EC_vs_non-HIC** | **-0.238** | **0.236** | **ns** |
| **Panel2_2669_CD4+.Th1_CXCR4+** | **EC_vs_non-HIC** | **0.212** | **0.246** | **ns** |
| **Panel2_2044_CD4+.Tem** | **EC_vs_non-HIC** | **-0.249** | **0.247** | **ns** |
| **Panel2_2604_CD4+.Tnaive_CXCR3+** | **EC_vs_non-HIC** | **-0.231** | **0.247** | **ns** |
| **Panel2_2340_CD8+.Tc1/17_HLA-DR+** | **EC_vs_non-HIC** | **-0.250** | **0.248** | **ns** |
| **Panel2_2431_CD4+.Temra_CCR5+** | **EC_vs_non-HIC** | **0.243** | **0.248** | **ns** |
| **Panel2_2739_CD8+.Tcm_CXCR5+** | **EC_vs_non-HIC** | **-0.248** | **0.251** | **ns** |
| **Panel2_2059_CD8+.Tcm** | **EC_vs_non-HIC** | **-0.240** | **0.254** | **ns** |
| **Panel3_3096_Unswitched.Memory.B.cells_CD81+** | **EC_vs_non-HIC** | **0.245** | **0.258** | **ns** |
| **Panel1_1186_mDC_CD86+** | **EC_vs_non-HIC** | **0.220** | **0.270** | **ns** |
| **Panel2_2454_CD8+.Tc2_CCR5+** | **EC_vs_non-HIC** | **0.228** | **0.271** | **ns** |
| **Panel2_2626_CD8+.Tem_CXCR3+** | **EC_vs_non-HIC** | **-0.237** | **0.271** | **ns** |
| **Panel1_1045_TCRyd** | **EC_vs_non-HIC** | **-0.249** | **0.272** | **ns** |
| **Panel3_3095_Switched.Memory.B.cells_CD81+** | **EC_vs_non-HIC** | **-0.239** | **0.276** | **ns** |
| **Panel2_2646_Treg_CXCR4+** | **EC_vs_non-HIC** | **0.192** | **0.281** | **ns** |
| **Panel2_2160_CD8+.Tnaive_PD1+** | **EC_vs_non-HIC** | **-0.226** | **0.283** | **ns** |
| **Panel1_1069_Bc_CD86-PDL1+** | **EC_vs_non-HIC** | **-0.218** | **0.288** | **ns** |
| **Panel2_2147_CD4+.Th1_PD1+** | **EC_vs_non-HIC** | **-0.231** | **0.289** | **ns** |
| **Panel1_1268_NK_CD56+CD16+_CXCR4+** | **EC_vs_non-HIC** | **0.215** | **0.292** | **ns** |
| **Panel2_2661_CD4+.Tcm_CXCR4+** | **EC_vs_non-HIC** | **0.170** | **0.297** | **ns** |
| **Panel1_1311_NCM_CD14+CD16+_CCR5+** | **EC_vs_non-HIC** | **0.211** | **0.298** | **ns** |
| **Panel1_1038_mDC** | **EC_vs_non-HIC** | **0.211** | **0.305** | **ns** |
| **Panel2_2638_CD56+CD3+_CXCR4+** | **EC_vs_non-HIC** | **0.196** | **0.305** | **ns** |
| **Panel2_2231_NKcells_CD38+** | **EC_vs_non-HIC** | **0.225** | **0.307** | **ns** |
| **Panel2_2357_mTreg_CCR4+** | **EC_vs_non-HIC** | **-0.219** | **0.307** | **ns** |
| **Panel2_2260_Tfh_2_CD38+** | **EC_vs_non-HIC** | **-0.209** | **0.308** | **ns** |
| **Panel2_2551_Tfh_17_CCR7+** | **EC_vs_non-HIC** | **0.210** | **0.310** | **ns** |
| **Panel2_2036_Tfh_2** | **EC_vs_non-HIC** | **-0.212** | **0.316** | **ns** |
| **Panel2_2648_nTreg_CXCR4+** | **EC_vs_non-HIC** | **0.192** | **0.316** | **ns** |
| **Panel2_2021_Treg** | **EC_vs_non-HIC** | **-0.215** | **0.318** | **ns** |
| **Panel2_2082_CD8+PD1+** | **EC_vs_non-HIC** | **-0.209** | **0.320** | **ns** |
| **Panel3_3082_SMBC_IgE-IgA+** | **EC_vs_non-HIC** | **0.204** | **0.329** | **ns** |
| **Panel2_2119_CD4+CD8+_PD1+** | **EC_vs_non-HIC** | **-0.211** | **0.329** | **ns** |
| **Panel2_2150_CD4+.Th1/17_PD1+** | **EC_vs_non-HIC** | **-0.210** | **0.331** | **ns** |
| **Panel1_1027_DC** | **EC_vs_non-HIC** | **-0.214** | **0.334** | **ns** |
| **Panel2_2125_mTreg_PD1+** | **EC_vs_non-HIC** | **-0.186** | **0.336** | **ns** |
| **Panel2_2234_CD4-CD8+_CD38+** | **EC_vs_non-HIC** | **0.192** | **0.342** | **ns** |
| **Panel1_1349_mDC_CD16-CD1c+_CCR5+** | **EC_vs_non-HIC** | **-0.179** | **0.342** | **ns** |
| **Panel2_2664_CD4+.Tem_CXCR4+** | **EC_vs_non-HIC** | **0.153** | **0.344** | **ns** |
| **Panel2_2549_Tfh_1_CCR7+** | **EC_vs_non-HIC** | **0.197** | **0.345** | **ns** |
| **Panel2_2335_CD8+.Temra_HLA-DR+** | **EC_vs_non-HIC** | **0.207** | **0.347** | **ns** |
| **Panel2_2091_CD8+HLA-DR+CD38-** | **EC_vs_non-HIC** | **0.195** | **0.349** | **ns** |
| **Panel2_2449_CD8+.Tcm_CCR5+** | **EC_vs_non-HIC** | **-0.195** | **0.350** | **ns** |
| **Panel2_2438_CD4+.Th2_CCR5+** | **EC_vs_non-HIC** | **-0.179** | **0.354** | **ns** |
| **Panel2_2524_CD4-CD8+_CCR7+** | **EC_vs_non-HIC** | **0.181** | **0.355** | **ns** |
| **Panel2_2668_Tfh_1/17_CXCR4+** | **EC_vs_non-HIC** | **0.158** | **0.356** | **ns** |
| **Panel2_2683_CD8+.Temra_CXCR4+** | **EC_vs_non-HIC** | **0.161** | **0.358** | **ns** |
| **Panel1_1225_Bc_PDL1+** | **EC_vs_non-HIC** | **-0.187** | **0.363** | **ns** |
| **Panel2_2637_NKcells_CXCR4+** | **EC_vs_non-HIC** | **0.175** | **0.366** | **ns** |
| **Panel2_2489_CD4+.Temra_CCR6+** | **EC_vs_non-HIC** | **0.198** | **0.366** | **ns** |
| **Panel1_1294_mDC_CXCR4+** | **EC_vs_non-HIC** | **0.180** | **0.372** | **ns** |
| **Panel2_2740_CD8+.Tnaive_CXCR5+** | **EC_vs_non-HIC** | **-0.191** | **0.376** | **ns** |
| **Panel2_2256_CD4+.Tnaive_CD38+** | **EC_vs_non-HIC** | **-0.171** | **0.376** | **ns** |
| **Panel2_2437_CD4+.Th1_CCR5+** | **EC_vs_non-HIC** | **0.192** | **0.377** | **ns** |
| **Panel1_1310_IM_CD14++CD16+_CCR5+** | **EC_vs_non-HIC** | **0.172** | **0.384** | **ns** |
| **Panel2_2275_CD8+.Tcm_CD38+** | **EC_vs_non-HIC** | **0.168** | **0.394** | **ns** |
| **Panel2_2509_CD8+.Temra_CCR6+** | **EC_vs_non-HIC** | **-0.169** | **0.399** | **ns** |
| **Panel2_2583_CD4+CD8+_CXCR3+** | **EC_vs_non-HIC** | **0.185** | **0.402** | **ns** |
| **Panel2_2695_NKcells_CXCR5+** | **EC_vs_non-HIC** | **-0.178** | **0.404** | **ns** |
| **Panel2_2373_CD4+.Temra_CCR4+** | **EC_vs_non-HIC** | **-0.179** | **0.411** | **ns** |
| **Panel2_2394_CD8+.Tem_CCR4+** | **EC_vs_non-HIC** | **-0.170** | **0.416** | **ns** |
| **Panel2_2391_CD8+.Tcm_CCR4+** | **EC_vs_non-HIC** | **-0.178** | **0.417** | **ns** |
| **Panel2_2126_nTreg_PD1+** | **EC_vs_non-HIC** | **-0.162** | **0.422** | **ns** |
| **Panel2_2052_CD4+.Th2** | **EC_vs_non-HIC** | **0.171** | **0.426** | **ns** |
| **Panel1_1292_DC_CXCR4+** | **EC_vs_non-HIC** | **0.151** | **0.427** | **ns** |
| **Panel2_2241_mTreg_CD38+** | **EC_vs_non-HIC** | **-0.154** | **0.429** | **ns** |
| **Panel2_2040_Tfh_17** | **EC_vs_non-HIC** | **-0.162** | **0.435** | **ns** |
| **Panel2_2013_CD56+CD3+** | **EC_vs_non-HIC** | **0.171** | **0.437** | **ns** |
| **Panel1_1286_TCRvd1_CXCR4+** | **EC_vs_non-HIC** | **0.151** | **0.441** | **ns** |
| **Panel2_2672_CD4+.Th1/17_CXCR4+** | **EC_vs_non-HIC** | **0.128** | **0.450** | **ns** |
| **Panel2_2663_CD4+.Temra_CXCR4+** | **EC_vs_non-HIC** | **0.136** | **0.452** | **ns** |
| **Panel1_1032_NK_CD56++CD16-** | **EC_vs_non-HIC** | **0.162** | **0.452** | **ns** |
| **Panel2_2094_CD4+CXCR4-CCR5+** | **EC_vs_non-HIC** | **-0.155** | **0.453** | **ns** |
| **Panel2_2686_CD8+.Tc2_CXCR4+** | **EC_vs_non-HIC** | **0.133** | **0.453** | **ns** |
| **Panel2_2647_mTreg_CXCR4+** | **EC_vs_non-HIC** | **0.130** | **0.453** | **ns** |
| **Panel1_1309_CM_CD14++CD16-_CCR5+** | **EC_vs_non-HIC** | **0.142** | **0.455** | **ns** |
| **Panel1_1031_NK_CD56-CD16+** | **EC_vs_non-HIC** | **-0.169** | **0.455** | **ns** |
| **Panel2_2405_NKcells_CCR5+** | **EC_vs_non-HIC** | **-0.153** | **0.467** | **ns** |
| **Panel2_2409_CD4+CD8+_CCR5+** | **EC_vs_non-HIC** | **0.158** | **0.468** | **ns** |
| **Panel1_1346_DC_CCR5+** | **EC_vs_non-HIC** | **-0.150** | **0.468** | **ns** |
| **Panel2_2641_CD4+CD8+_CXCR4+** | **EC_vs_non-HIC** | **0.136** | **0.475** | **ns** |
| **Panel2_2277_CD8+.Temra_CD38+** | **EC_vs_non-HIC** | **0.148** | **0.479** | **ns** |
| **Panel1_1285_TCRyd_CXCR4+** | **EC_vs_non-HIC** | **0.134** | **0.487** | **ns** |
| **Panel2_2580_CD56+CD3+_CXCR3+** | **EC_vs_non-HIC** | **-0.148** | **0.488** | **ns** |
| **Panel2_2553_CD4+.Th1_CCR7+** | **EC_vs_non-HIC** | **0.152** | **0.488** | **ns** |
| **Panel2_2508_CD8+.Tnaive_CCR6+** | **EC_vs_non-HIC** | **-0.134** | **0.493** | **ns** |
| **Panel2_2521_NKcells_CCR7+** | **EC_vs_non-HIC** | **0.149** | **0.494** | **ns** |
| **Panel2_2606_CD4+.Tem_CXCR3+** | **EC_vs_non-HIC** | **0.147** | **0.495** | **ns** |
| **Panel2_2235_CD4+CD8+_CD38+** | **EC_vs_non-HIC** | **-0.130** | **0.496** | **ns** |
| **Panel1_1210_NK-T.like_PDL1+** | **EC_vs_non-HIC** | **-0.118** | **0.497** | **ns** |
| **Panel1_1322_NK_CD56+CD16+_CCR5+** | **EC_vs_non-HIC** | **-0.150** | **0.499** | **ns** |
| **Panel2_2552_Tfh_1/17_CCR7+** | **EC_vs_non-HIC** | **0.139** | **0.506** | **ns** |
| **Panel1_1257_NCM_CD14+CD16+_CXCR4+** | **EC_vs_non-HIC** | **0.134** | **0.507** | **ns** |
| **Panel1_1070_Bc_CD86+PDL1-** | **EC_vs_non-HIC** | **0.126** | **0.516** | **ns** |
| **Panel2_2720_CD4+.Tnaive_CXCR5+** | **EC_vs_non-HIC** | **0.140** | **0.521** | **ns** |
| **Panel2_2665_Tfh_1_CXCR4+** | **EC_vs_non-HIC** | **0.109** | **0.525** | **ns** |
| **Panel1_1065_Bc_CD40+** | **EC_vs_non-HIC** | **-0.140** | **0.530** | **ns** |
| **Panel1_1015_Monocytes** | **EC_vs_non-HIC** | **0.140** | **0.532** | **ns** |
| **Panel2_2474_nTreg_CCR6+** | **EC_vs_non-HIC** | **-0.125** | **0.533** | **ns** |
| **Panel2_2392_CD8+.Tnaive_CCR4+** | **EC_vs_non-HIC** | **-0.133** | **0.534** | **ns** |
| **Panel2_2450_CD8+.Tnaive_CCR5+** | **EC_vs_non-HIC** | **-0.125** | **0.535** | **ns** |
| **Panel2_2624_CD8+.Tnaive_CXCR3+** | **EC_vs_non-HIC** | **-0.137** | **0.537** | **ns** |
| **Panel2_2240_Treg_CD38+** | **EC_vs_non-HIC** | **-0.120** | **0.538** | **ns** |
| **Panel2_2722_CD4+.Tem_CXCR5+** | **EC_vs_non-HIC** | **0.131** | **0.540** | **ns** |
| **Panel2_2159_CD8+.Tcm_PD1+** | **EC_vs_non-HIC** | **-0.127** | **0.546** | **ns** |
| **Panel2_2681_CD8+.Tcm_CXCR4+** | **EC_vs_non-HIC** | **0.103** | **0.548** | **ns** |
| **Panel2_2721_CD4+.Temra_CXCR5+** | **EC_vs_non-HIC** | **-0.128** | **0.552** | **ns** |
| **Panel1_1348_mDC_CCR5+** | **EC_vs_non-HIC** | **-0.122** | **0.555** | **ns** |
| **Panel2_2472_Treg_CCR6+** | **EC_vs_non-HIC** | **-0.118** | **0.557** | **ns** |
| **Panel2_2338_CD8+.Tc2_HLA-DR+** | **EC_vs_non-HIC** | **0.127** | **0.558** | **ns** |
| **Panel1_1033_NK_CD56++CD16+** | **EC_vs_non-HIC** | **0.129** | **0.564** | **ns** |
| **Panel1_1041_mDC_CD16-CD1c+** | **EC_vs_non-HIC** | **-0.124** | **0.571** | **ns** |
| **Panel2_2464_CD56+CD3+_CCR6+** | **EC_vs_non-HIC** | **0.116** | **0.575** | **ns** |
| **Panel2_2315_CD4+.Temra_HLA-DR+** | **EC_vs_non-HIC** | **-0.119** | **0.577** | **ns** |
| **Panel2_2662_CD4+.Tnaive_CXCR4+** | **EC_vs_non-HIC** | **0.099** | **0.578** | **ns** |
| **Panel2_2333_CD8+.Tcm_HLA-DR+** | **EC_vs_non-HIC** | **0.121** | **0.581** | **ns** |
| **Panel2_2741_CD8+.Temra_CXCR5+** | **EC_vs_non-HIC** | **0.119** | **0.582** | **ns** |
| **Panel2_2100_CD8+CXCR4+CCR5+** | **EC_vs_non-HIC** | **0.099** | **0.582** | **ns** |
| **Panel2_2667_Tfh_17_CXCR4+** | **EC_vs_non-HIC** | **0.096** | **0.582** | **ns** |
| **Panel2_2625_CD8+.Temra_CXCR3+** | **EC_vs_non-HIC** | **-0.118** | **0.585** | **ns** |
| **Panel3_3076_SMBC_IgG+IgA+** | **EC_vs_non-HIC** | **-0.106** | **0.586** | **ns** |
| **Panel2_2255_CD4+.Tcm_CD38+** | **EC_vs_non-HIC** | **-0.105** | **0.586** | **ns** |
| **Panel2_2090_CD8+HLA-DR-CD38+** | **EC_vs_non-HIC** | **0.104** | **0.591** | **ns** |
| **Panel1_1201_CM_CD14++CD16-_PDL1+** | **EC_vs_non-HIC** | **-0.102** | **0.594** | **ns** |
| **Panel2_2086_CD4+HLA-DR-CD38+** | **EC_vs_non-HIC** | **0.100** | **0.596** | **ns** |
| **Panel1_1184_DC_CD86+** | **EC_vs_non-HIC** | **0.110** | **0.597** | **ns** |
| **Panel2_2550_Tfh_2_CCR7+** | **EC_vs_non-HIC** | **0.105** | **0.606** | **ns** |
| **Panel1_1231_TCRyd_PDL1+** | **EC_vs_non-HIC** | **-0.092** | **0.621** | **ns** |
| **Panel2_2014_NKcells** | **EC_vs_non-HIC** | **-0.107** | **0.627** | **ns** |
| **Panel2_2045_CD4+.Temra** | **EC_vs_non-HIC** | **-0.105** | **0.629** | **ns** |
| **Panel2_2242_nTreg_CD38+** | **EC_vs_non-HIC** | **-0.097** | **0.629** | **ns** |
| **Panel1_1147_CM_CD14++CD16-_CD86+** | **EC_vs_non-HIC** | **-0.093** | **0.629** | **ns** |
| **Panel1_1295_mDC_CD16-CD1c+_CXCR4+** | **EC_vs_non-HIC** | **0.090** | **0.631** | **ns** |
| **Panel2_2704_Treg_CXCR5+** | **EC_vs_non-HIC** | **0.103** | **0.632** | **ns** |
| **Panel2_2743_CD8+.Tc1_CXCR5+** | **EC_vs_non-HIC** | **0.103** | **0.634** | **ns** |
| **Panel2_2463_NKcells_CCR6+** | **EC_vs_non-HIC** | **-0.086** | **0.649** | **ns** |
| **Panel2_2051_CD4+.Th1** | **EC_vs_non-HIC** | **-0.099** | **0.649** | **ns** |
| **Panel2_2162_CD8+.Tem_PD1+** | **EC_vs_non-HIC** | **0.098** | **0.654** | **ns** |
| **Panel1_1279_Bc_CXCR4+** | **EC_vs_non-HIC** | **0.090** | **0.655** | **ns** |
| **Panel2_2429_CD4+.Tcm_CCR5+** | **EC_vs_non-HIC** | **0.093** | **0.657** | **ns** |
| **Panel2_2728_CD4+.Th2_CXCR5+** | **EC_vs_non-HIC** | **0.093** | **0.663** | **ns** |
| **Panel2_2671_CD4+.Th17_CXCR4+** | **EC_vs_non-HIC** | **0.074** | **0.667** | **ns** |
| **Panel1_1249_Basophils_CXCR4+** | **EC_vs_non-HIC** | **0.075** | **0.667** | **ns** |
| **Panel1_1148_IM_CD14++CD16+_CD86+** | **EC_vs_non-HIC** | **-0.081** | **0.669** | **ns** |
| **Panel3_3069_AM.(CD27+CD21-)** | **EC_vs_non-HIC** | **0.094** | **0.669** | **ns** |
| **Panel2_2259_Tfh_1_CD38+** | **EC_vs_non-HIC** | **0.081** | **0.680** | **ns** |
| **Panel2_2314_CD4+.Tnaive_HLA-DR+** | **EC_vs_non-HIC** | **0.086** | **0.684** | **ns** |
| **Panel2_2149_CD4+.Th17_PD1+** | **EC_vs_non-HIC** | **-0.077** | **0.691** | **ns** |
| **Panel1_1187_mDC_CD16-CD1c+_CD86+** | **EC_vs_non-HIC** | **0.073** | **0.694** | **ns** |
| **Panel2_2706_nTreg_CXCR5+** | **EC_vs_non-HIC** | **0.083** | **0.702** | **ns** |
| **Panel2_2572_CD8+.Tc1/17_CCR7+** | **EC_vs_non-HIC** | **-0.082** | **0.703** | **ns** |
| **Panel1_1302_Eosinophils_CCR5+** | **EC_vs_non-HIC** | **0.080** | **0.703** | **ns** |
| **Panel3_3029_Switched.Memory.B.cells** | **EC_vs_non-HIC** | **-0.081** | **0.713** | **ns** |
| **Panel1_1028_Basophils** | **EC_vs_non-HIC** | **-0.083** | **0.715** | **ns** |
| **Panel2_2430_CD4+.Tnaive_CCR5+** | **EC_vs_non-HIC** | **-0.078** | **0.717** | **ns** |
| **Panel2_2705_mTreg_CXCR5+** | **EC_vs_non-HIC** | **0.077** | **0.722** | **ns** |
| **Panel2_2729_CD4+.Th17_CXCR5+** | **EC_vs_non-HIC** | **0.076** | **0.724** | **ns** |
| **Panel2_2300_nTreg_HLA-DR+** | **EC_vs_non-HIC** | **-0.072** | **0.725** | **ns** |
| **Panel1_1247_Neutrophils_CXCR4+** | **EC_vs_non-HIC** | **-0.073** | **0.726** | **ns** |
| **Panel2_2415_mTreg_CCR5+** | **EC_vs_non-HIC** | **0.068** | **0.728** | **ns** |
| **Panel2_2372_CD4+.Tnaive_CCR4+** | **EC_vs_non-HIC** | **-0.075** | **0.729** | **ns** |
| **Panel3_3028_Unswitched.Memory.B.cells** | **EC_vs_non-HIC** | **-0.074** | **0.737** | **ns** |
| **Panel3_3072_TLM.(CD27-CD21-)** | **EC_vs_non-HIC** | **0.073** | **0.737** | **ns** |
| **Panel2_2727_CD4+.Th1_CXCR5+** | **EC_vs_non-HIC** | **0.071** | **0.739** | **ns** |
| **Panel1_1035_NK_CD56+CD16+** | **EC_vs_non-HIC** | **0.073** | **0.743** | **ns** |
| **Panel2_2070_CD8+.Tc2** | **EC_vs_non-HIC** | **0.068** | **0.750** | **ns** |
| **Panel2_2257_CD4+.Temra_CD38+** | **EC_vs_non-HIC** | **-0.062** | **0.756** | **ns** |
| **Panel2_2043_CD4+.Tcm** | **EC_vs_non-HIC** | **-0.066** | **0.757** | **ns** |
| **Panel2_2148_CD4+.Th2_PD1+** | **EC_vs_non-HIC** | **-0.057** | **0.765** | **ns** |
| **Panel2_2579_NKcells_CXCR3+** | **EC_vs_non-HIC** | **-0.064** | **0.767** | **ns** |
| **Panel1_1171_Bc_CD86+** | **EC_vs_non-HIC** | **0.052** | **0.770** | **ns** |
| **Panel1_1019_NCM_CD14+CD16+** | **EC_vs_non-HIC** | **-0.063** | **0.772** | **ns** |
| **Panel2_2699_CD4+CD8+_CXCR5+** | **EC_vs_non-HIC** | **-0.063** | **0.772** | **ns** |
| **Panel2_2744_CD8+.Tc2_CXCR5+** | **EC_vs_non-HIC** | **-0.063** | **0.772** | **ns** |
| **Panel2_2730_CD4+.Th1/17_CXCR5+** | **EC_vs_non-HIC** | **0.061** | **0.775** | **ns** |
| **Panel2_2408_CD4-CD8+_CCR5+** | **EC_vs_non-HIC** | **-0.057** | **0.779** | **ns** |
| **Panel2_2569_CD8+.Tc1_CCR7+** | **EC_vs_non-HIC** | **0.060** | **0.780** | **ns** |
| **Panel2_2666_Tfh_2_CXCR4+** | **EC_vs_non-HIC** | **0.047** | **0.782** | **ns** |
| **Panel2_2742_CD8+.Tem_CXCR5+** | **EC_vs_non-HIC** | **0.058** | **0.791** | **ns** |
| **Panel2_2456_CD8+.Tc1/17_CCR5+** | **EC_vs_non-HIC** | **0.058** | **0.792** | **ns** |
| **Panel3_3068_NBC_CD21+** | **EC_vs_non-HIC** | **0.058** | **0.795** | **ns** |
| **Panel1_1255_CM_CD14++CD16-_CXCR4+** | **EC_vs_non-HIC** | **0.053** | **0.802** | **ns** |
| **Panel1_1301_Neutrophils_CCR5+** | **EC_vs_non-HIC** | **-0.051** | **0.809** | **ns** |
| **Panel3_3070_IM.(CD27-CD21+)** | **EC_vs_non-HIC** | **-0.052** | **0.811** | **ns** |
| **Panel2_2116_CD56+CD3+_PD1+** | **EC_vs_non-HIC** | **0.053** | **0.812** | **ns** |
| **Panel2_2261_Tfh_17_CD38+** | **EC_vs_non-HIC** | **-0.047** | **0.812** | **ns** |
| **Panel3_3071_RM.(CD27+CD21+)** | **EC_vs_non-HIC** | **0.051** | **0.812** | **ns** |
| **Panel2_2719_CD4+.Tcm_CXCR5+** | **EC_vs_non-HIC** | **-0.051** | **0.815** | **ns** |
| **Panel2_2490_CD4+.Tem_CCR6+** | **EC_vs_non-HIC** | **0.050** | **0.816** | **ns** |
| **Panel2_2407_CD4+CD8-_CCR5+** | **EC_vs_non-HIC** | **0.048** | **0.819** | **ns** |
| **Panel1_1178_TCRvd1_CD86+** | **EC_vs_non-HIC** | **-0.046** | **0.834** | **ns** |
| **Panel2_2351_CD4+CD8+_CCR4+** | **EC_vs_non-HIC** | **-0.045** | **0.837** | **ns** |
| **Panel2_2688_CD8+.Tc1/17_CXCR4+** | **EC_vs_non-HIC** | **0.034** | **0.837** | **ns** |
| **Panel2_2069_CD8+.Tc1** | **EC_vs_non-HIC** | **0.043** | **0.840** | **ns** |
| **Panel2_2414_Treg_CCR5+** | **EC_vs_non-HIC** | **-0.040** | **0.841** | **ns** |
| **Panel1_1123_TCRyd_HLA-DR+** | **EC_vs_non-HIC** | **-0.045** | **0.843** | **ns** |
| **Panel2_2697_CD4+CD8-_CXCR5+** | **EC_vs_non-HIC** | **0.042** | **0.846** | **ns** |
| **Panel3_3094_Plasma.Cells_CD81+** | **EC_vs_non-HIC** | **-0.031** | **0.859** | **ns** |
| **Panel2_2451_CD8+.Temra_CCR5+** | **EC_vs_non-HIC** | **-0.036** | **0.861** | **ns** |
| **Panel2_2262_Tfh_1/17_CD38+** | **EC_vs_non-HIC** | **-0.034** | **0.864** | **ns** |
| **Panel2_2605_CD4+.Temra_CXCR3+** | **EC_vs_non-HIC** | **0.036** | **0.864** | **ns** |
| **Panel2_2439_CD4+.Th17_CCR5+** | **EC_vs_non-HIC** | **0.033** | **0.866** | **ns** |
| **Panel2_2348_CD56+CD3+_CCR4+** | **EC_vs_non-HIC** | **-0.033** | **0.867** | **ns** |
| **Panel2_2015_Tcells** | **EC_vs_non-HIC** | **0.034** | **0.872** | **ns** |
| **Panel2_2074_CD8+.Tc17** | **EC_vs_non-HIC** | **-0.033** | **0.877** | **ns** |
| **Panel2_2522_CD56+CD3+_CCR7+** | **EC_vs_non-HIC** | **-0.032** | **0.882** | **ns** |
| **Panel1_1034_NK_CD56+CD16-** | **EC_vs_non-HIC** | **0.032** | **0.885** | **ns** |
| **Panel3_3010_CD19+** | **EC_vs_non-HIC** | **-0.031** | **0.887** | **ns** |
| **Panel2_2590_nTreg_CXCR3+** | **EC_vs_non-HIC** | **-0.027** | **0.887** | **ns** |
| **Panel2_2232_CD56+CD3+_CD38+** | **EC_vs_non-HIC** | **0.026** | **0.897** | **ns** |
| **Panel2_2276_CD8+.Tnaive_CD38+** | **EC_vs_non-HIC** | **-0.025** | **0.897** | **ns** |
| **Panel2_2161_CD8+.Temra_PD1+** | **EC_vs_non-HIC** | **0.027** | **0.901** | **ns** |
| **Panel2_2416_nTreg_CCR5+** | **EC_vs_non-HIC** | **0.024** | **0.904** | **ns** |
| **Panel2_2233_CD4+CD8-_CD38+** | **EC_vs_non-HIC** | **0.022** | **0.907** | **ns** |
| **Panel1_1202_IM_CD14++CD16+_PDL1+** | **EC_vs_non-HIC** | **0.024** | **0.907** | **ns** |
| **Panel1_1238_DC_PDL1+** | **EC_vs_non-HIC** | **0.022** | **0.908** | **ns** |
| **Panel2_2453_CD8+.Tc1_CCR5+** | **EC_vs_non-HIC** | **-0.024** | **0.909** | **ns** |
| **Panel1_1287_TCRvd2_CXCR4+** | **EC_vs_non-HIC** | **-0.021** | **0.911** | **ns** |
| **Panel2_2473_mTreg_CCR6+** | **EC_vs_non-HIC** | **-0.022** | **0.916** | **ns** |
| **Panel2_2589_mTreg_CXCR3+** | **EC_vs_non-HIC** | **-0.022** | **0.918** | **ns** |
| **Panel2_2698_CD4-CD8+_CXCR5+** | **EC_vs_non-HIC** | **0.019** | **0.928** | **ns** |
| **Panel1_1256_IM_CD14++CD16+_CXCR4+** | **EC_vs_non-HIC** | **-0.016** | **0.939** | **ns** |
| **Panel2_2525_CD4+CD8+_CCR7+** | **EC_vs_non-HIC** | **0.015** | **0.944** | **ns** |
| **Panel1_1177_TCRyd_CD86+** | **EC_vs_non-HIC** | **-0.015** | **0.944** | **ns** |
| **Panel2_2263_CD4+.Th1_CD38+** | **EC_vs_non-HIC** | **0.013** | **0.946** | **ns** |
| **Panel1_1071_Bc_CD86+PDL1+** | **EC_vs_non-HIC** | **0.012** | **0.948** | **ns** |
| **Panel2_2019_CD4+CD8+** | **EC_vs_non-HIC** | **0.014** | **0.950** | **ns** |
| **Panel1_1248_Eosinophils_CXCR4+** | **EC_vs_non-HIC** | **0.012** | **0.951** | **ns** |
| **Panel1_1341_TCRvd2_CCR5+** | **EC_vs_non-HIC** | **-0.012** | **0.956** | **ns** |
| **Panel2_2293_CD4+CD8+_HLA-DR+** | **EC_vs_non-HIC** | **-0.009** | **0.968** | **ns** |
| **Panel2_2682_CD8+.Tnaive_CXCR4+** | **EC_vs_non-HIC** | **0.006** | **0.975** | **ns** |
| **Panel2_2452_CD8+.Tem_CCR5+** | **EC_vs_non-HIC** | **0.005** | **0.981** | **ns** |
| **Panel2_2588_Treg_CXCR3+** | **EC_vs_non-HIC** | **-0.004** | **0.985** | **ns** |
| **Panel2_2163_CD8+.Tc1_PD1+** | **EC_vs_non-HIC** | **0.004** | **0.985** | **ns** |
| **Panel2_2334_CD8+.Tnaive_HLA-DR+** | **EC_vs_non-HIC** | **-0.003** | **0.987** | **ns** |
| **Panel2_2406_CD56+CD3+_CCR5+** | **EC_vs_non-HIC** | **0.002** | **0.992** | **ns** |

**Supplementary TABLE 8. Comparison of the expression levels (MFI) of PD1, HLA-DR, CD38, CXCR4 and CCR5 on T cells between HIC and non-HIC.**

| cell_subset | estimate | group1 | group2 | p-value | method | p_adj | p.adj.signif |
| --- | --- | --- | --- | --- | --- | --- | --- |
| Panel2_MFI_CD38_2275_CD8+.Tcm_CD38+ | 1847.62 | HIC | non_HIC | 0.00 | Wilcoxon | 0.02 | * |
| Panel2_MFI_HLA-DR_2322_CD4+.Th2_HLA-DR+ | -7282.22 | HIC | non_HIC | 0.00 | Wilcoxon | 0.02 | * |
| Panel2_MFI_CCR5_2408_CD4-CD8+_CCR5+ | -2645.61 | HIC | non_HIC | 0.00 | Wilcoxon | 0.02 | * |
| Panel2_MFI_CCR5_2451_CD8+.Temra_CCR5+ | -2796.77 | HIC | non_HIC | 0.00 | Wilcoxon | 0.02 | * |
| Panel2_MFI_CCR5_2453_CD8+.Tc1_CCR5+ | -2248.33 | HIC | non_HIC | 0.00 | Wilcoxon | 0.02 | * |
| Panel2_MFI_HLA-DR_2314_CD4+.Tnaive_HLA-DR+ | -4226.82 | HIC | non_HIC | 0.00 | Wilcoxon | 0.02 | * |
| Panel2_MFI_CCR5_2452_CD8+.Tem_CCR5+ | -2310.28 | HIC | non_HIC | 0.00 | Wilcoxon | 0.02 | * |
| Panel2_MFI_HLA-DR_2299_mTreg_HLA-DR+ | -6682.18 | HIC | non_HIC | 0.00 | Wilcoxon | 0.03 | * |
| Panel2_MFI_CCR5_2098_CD8+CXCR4-CCR5+ | -2211.82 | HIC | non_HIC | 0.00 | Wilcoxon | 0.03 | * |
| Panel2_MFI_CD38_2280_CD8+.Tc2_CD38+ | 773.71 | HIC | non_HIC | 0.00 | Wilcoxon | 0.03 | * |
| Panel2_MFI_HLA-DR_2335_CD8+.Temra_HLA-DR+ | 2529.57 | HIC | non_HIC | 0.00 | Wilcoxon | 0.03 | * |
| Panel2_MFI_HLA-DR_2336_CD8+.Tem_HLA-DR+ | 3542.83 | HIC | non_HIC | 0.00 | Wilcoxon | 0.03 | * |
| Panel2_MFI_HLA-DR_2292_CD4-CD8+_HLA-DR+ | 2913.74 | HIC | non_HIC | 0.00 | Wilcoxon | 0.03 | * |
| Panel2_MFI_HLA-DR_2323_CD4+.Th17_HLA-DR+ | -5206.60 | HIC | non_HIC | 0.00 | Wilcoxon | 0.03 | * |
| Panel2_MFI_HLA-DR_2313_CD4+.Tcm_HLA-DR+ | -4361.82 | HIC | non_HIC | 0.00 | Wilcoxon | 0.04 | * |
| Panel2_MFI_CCR5_2456_CD8+.Tc1/17_CCR5+ | -4556.08 | HIC | non_HIC | 0.00 | Wilcoxon | 0.04 | * |
| Panel2_MFI_CCR5_2100_CD8+CXCR4+CCR5+ | -2172.03 | HIC | non_HIC | 0.00 | Wilcoxon | 0.05 | ns |
| Panel2_MFI_CCR5_2407_CD4+CD8-_CCR5+ | -1542.68 | HIC | non_HIC | 0.00 | Wilcoxon | 0.05 | ns |
| Panel2_MFI_HLA-DR_2291_CD4+CD8-_HLA-DR+ | -4796.76 | HIC | non_HIC | 0.00 | Wilcoxon | 0.05 | ns |
| Panel2_MFI_CCR5_2415_mTreg_CCR5+ | -1496.75 | HIC | non_HIC | 0.00 | Wilcoxon | 0.05 | ns |
| Panel2_MFI_CD38_2278_CD8+.Tem_CD38+ | 567.09 | HIC | non_HIC | 0.00 | Wilcoxon | 0.05 | ns |
| Panel2_MFI_HLA-DR_2337_CD8+.Tc1_HLA-DR+ | 3128.86 | HIC | non_HIC | 0.00 | Wilcoxon | 0.05 | ns |
| Panel2_MFI_PD1_2164_CD8+.Tc2_PD1+ | 3388.10 | HIC | non_HIC | 0.00 | Wilcoxon | 0.05 | ns |
| Panel2_MFI_CCR5_2430_CD4+.Tnaive_CCR5+ | -4585.16 | HIC | non_HIC | 0.00 | Wilcoxon | 0.05 | ns |
| Panel2_MFI_HLA-DR_2340_CD8+.Tc1/17_HLA-DR+ | 4283.01 | HIC | non_HIC | 0.01 | Wilcoxon | 0.06 | ns |
| Panel2_MFI_PD1_2166_CD8+.Tc1/17_PD1+ | 1495.42 | HIC | non_HIC | 0.01 | Wilcoxon | 0.06 | ns |
| Panel2_MFI_CCR5_2096_CD4+CXCR4+CCR5+ | -1673.49 | HIC | non_HIC | 0.01 | Wilcoxon | 0.07 | ns |
| Panel2_MFI_PD1_2162_CD8+.Tem_PD1+ | 1840.50 | HIC | non_HIC | 0.01 | Wilcoxon | 0.07 | ns |
| Panel2_MFI_CD38_2279_CD8+.Tc1_CD38+ | 605.50 | HIC | non_HIC | 0.01 | Wilcoxon | 0.08 | ns |
| Panel2_MFI_CCR5_2094_CD4+CXCR4-CCR5+ | -1361.18 | HIC | non_HIC | 0.01 | Wilcoxon | 0.09 | ns |
| Panel2_MFI_HLA-DR_2092_CD8+HLA-DR+CD38+ | 5657.36 | HIC | non_HIC | 0.01 | Wilcoxon | 0.09 | ns |
| Panel2_MFI_HLA-DR_2298_Treg_HLA-DR+ | -5477.95 | HIC | non_HIC | 0.01 | Wilcoxon | 0.09 | ns |
| Panel2_MFI_CCR5_2406_CD56+CD3+_CCR5+ | -2777.55 | HIC | non_HIC | 0.02 | Wilcoxon | 0.10 | ns |
| Panel2_MFI_PD1_2163_CD8+.Tc1_PD1+ | 1525.41 | HIC | non_HIC | 0.02 | Wilcoxon | 0.10 | ns |
| Panel2_MFI_CCR5_2437_CD4+.Th1_CCR5+ | -1175.11 | HIC | non_HIC | 0.02 | Wilcoxon | 0.10 | ns |
| Panel2_MFI_CXCR4_2094_CD4+CXCR4-CCR5+ | 1187.59 | HIC | non_HIC | 0.02 | Wilcoxon | 0.10 | ns |
| Panel2_MFI_HLA-DR_2333_CD8+.Tcm_HLA-DR+ | 4818.04 | HIC | non_HIC | 0.02 | Wilcoxon | 0.10 | ns |
| Panel2_MFI_PD1_2140_CD4+.Tnaive_PD1+ | -1012.12 | HIC | non_HIC | 0.02 | Wilcoxon | 0.10 | ns |
| Panel2_MFI_HLA-DR_2091_CD8+HLA-DR+CD38- | 1631.77 | HIC | non_HIC | 0.02 | Wilcoxon | 0.11 | ns |
| Panel2_MFI_CCR5_2432_CD4+.Tem_CCR5+ | -1050.08 | HIC | non_HIC | 0.03 | Wilcoxon | 0.14 | ns |
| Panel2_MFI_CD38_2234_CD4-CD8+_CD38+ | 269.07 | HIC | non_HIC | 0.03 | Wilcoxon | 0.14 | ns |
| Panel2_MFI_CCR5_2099_CD8+CXCR4+CCR5- | -815.00 | HIC | non_HIC | 0.04 | Wilcoxon | 0.14 | ns |
| Panel2_MFI_CD38_2240_Treg_CD38+ | -701.95 | HIC | non_HIC | 0.04 | Wilcoxon | 0.15 | ns |
| Panel2_MFI_CCR5_2439_CD4+.Th17_CCR5+ | -874.82 | HIC | non_HIC | 0.05 | Wilcoxon | 0.17 | ns |
| Panel2_MFI_CCR5_2454_CD8+.Tc2_CCR5+ | -1518.86 | HIC | non_HIC | 0.05 | Wilcoxon | 0.17 | ns |
| Panel2_MFI_CD38_2242_nTreg_CD38+ | -520.26 | HIC | non_HIC | 0.05 | Wilcoxon | 0.17 | ns |
| Panel2_MFI_CCR5_2416_nTreg_CCR5+ | -5356.28 | HIC | non_HIC | 0.05 | Wilcoxon | 0.18 | ns |
| Panel2_MFI_CCR5_2431_CD4+.Temra_CCR5+ | -4580.07 | HIC | non_HIC | 0.06 | Wilcoxon | 0.19 | ns |
| Panel2_MFI_CCR5_2449_CD8+.Tcm_CCR5+ | -1318.48 | HIC | non_HIC | 0.06 | Wilcoxon | 0.19 | ns |
| Panel2_MFI_CD38_2235_CD4+CD8+_CD38+ | 592.91 | HIC | non_HIC | 0.06 | Wilcoxon | 0.20 | ns |
| Panel2_MFI_CCR5_2414_Treg_CCR5+ | -1269.94 | HIC | non_HIC | 0.07 | Wilcoxon | 0.21 | ns |
| Panel2_MFI_CCR5_2429_CD4+.Tcm_CCR5+ | -796.49 | HIC | non_HIC | 0.09 | Wilcoxon | 0.25 | ns |
| Panel2_MFI_CD38_2276_CD8+.Tnaive_CD38+ | 187.98 | HIC | non_HIC | 0.09 | Wilcoxon | 0.25 | ns |
| Panel2_MFI_HLA-DR_2088_CD4+HLA-DR+CD38+ | -6621.45 | HIC | non_HIC | 0.09 | Wilcoxon | 0.25 | ns |
| Panel2_MFI_PD1_2161_CD8+.Temra_PD1+ | 749.21 | HIC | non_HIC | 0.09 | Wilcoxon | 0.27 | ns |
| Panel2_MFI_HLA-DR_2338_CD8+.Tc2_HLA-DR+ | 2679.81 | HIC | non_HIC | 0.12 | Wilcoxon | 0.33 | ns |
| Panel2_MFI_PD1_2160_CD8+.Tnaive_PD1+ | -514.10 | HIC | non_HIC | 0.12 | Wilcoxon | 0.33 | ns |
| Panel2_MFI_CXCR4_2639_CD4+CD8-_CXCR4+ | 2616.03 | HIC | non_HIC | 0.13 | Wilcoxon | 0.34 | ns |
| Panel2_MFI_CD38_2090_CD8+HLA-DR-CD38+ | 169.78 | HIC | non_HIC | 0.13 | Wilcoxon | 0.34 | ns |
| Panel2_MFI_CD38_2263_CD4+.Th1_CD38+ | 377.89 | HIC | non_HIC | 0.14 | Wilcoxon | 0.34 | ns |
| Panel2_MFI_CXCR4_2640_CD4-CD8+_CXCR4+ | 2355.02 | HIC | non_HIC | 0.14 | Wilcoxon | 0.34 | ns |
| Panel2_MFI_CXCR4_2683_CD8+.Temra_CXCR4+ | 1582.71 | HIC | non_HIC | 0.13 | Wilcoxon | 0.34 | ns |
| Panel2_MFI_CXCR4_2684_CD8+.Tem_CXCR4+ | 1652.49 | HIC | non_HIC | 0.14 | Wilcoxon | 0.34 | ns |
| Panel2_MFI_PD1_2115_NKcells_PD1+ | 878.00 | HIC | non_HIC | 0.14 | Wilcoxon | 0.34 | ns |
| Panel2_MFI_PD1_2159_CD8+.Tcm_PD1+ | 953.48 | HIC | non_HIC | 0.15 | Wilcoxon | 0.34 | ns |
| Panel2_MFI_CD38_2266_CD4+.Th1/17_CD38+ | 183.30 | HIC | non_HIC | 0.15 | Wilcoxon | 0.35 | ns |
| Panel2_MFI_CCR5_2409_CD4+CD8+_CCR5+ | -1237.76 | HIC | non_HIC | 0.16 | Wilcoxon | 0.36 | ns |
| Panel2_MFI_CCR5_2440_CD4+.Th1/17_CCR5+ | -722.89 | HIC | non_HIC | 0.16 | Wilcoxon | 0.36 | ns |
| Panel2_MFI_CD38_2264_CD4+.Th2_CD38+ | 306.52 | HIC | non_HIC | 0.16 | Wilcoxon | 0.36 | ns |
| Panel2_MFI_CD38_2277_CD8+.Temra_CD38+ | 256.16 | HIC | non_HIC | 0.17 | Wilcoxon | 0.36 | ns |
| Panel2_MFI_CXCR4_2095_CD4+CXCR4+CCR5- | 2437.47 | HIC | non_HIC | 0.17 | Wilcoxon | 0.36 | ns |
| Panel2_MFI_HLA-DR_2289_NKcells_HLA-DR+ | 2268.90 | HIC | non_HIC | 0.16 | Wilcoxon | 0.36 | ns |
| Panel2_MFI_HLA-DR_2324_CD4+.Th1/17_HLA-DR+ | -2106.94 | HIC | non_HIC | 0.18 | Wilcoxon | 0.39 | ns |
| Panel2_MFI_HLA-DR_2293_CD4+CD8+_HLA-DR+ | 1636.83 | HIC | non_HIC | 0.19 | Wilcoxon | 0.39 | ns |
| Panel2_MFI_CD38_2241_mTreg_CD38+ | -546.75 | HIC | non_HIC | 0.21 | Wilcoxon | 0.42 | ns |
| Panel2_MFI_CCR5_2095_CD4+CXCR4+CCR5- | -511.34 | HIC | non_HIC | 0.21 | Wilcoxon | 0.42 | ns |
| Panel2_MFI_CD38_2258_CD4+.Tem_CD38+ | 187.36 | HIC | non_HIC | 0.22 | Wilcoxon | 0.44 | ns |
| Panel2_MFI_CXCR4_2646_Treg_CXCR4+ | 1423.35 | HIC | non_HIC | 0.23 | Wilcoxon | 0.44 | ns |
| Panel2_MFI_CXCR4_2661_CD4+.Tcm_CXCR4+ | 2007.42 | HIC | non_HIC | 0.24 | Wilcoxon | 0.44 | ns |
| Panel2_MFI_CXCR4_2664_CD4+.Tem_CXCR4+ | 795.07 | HIC | non_HIC | 0.23 | Wilcoxon | 0.44 | ns |
| Panel2_MFI_CXCR4_2670_CD4+.Th2_CXCR4+ | 1526.67 | HIC | non_HIC | 0.23 | Wilcoxon | 0.44 | ns |
| Panel2_MFI_CXCR4_2685_CD8+.Tc1_CXCR4+ | 1435.43 | HIC | non_HIC | 0.25 | Wilcoxon | 0.46 | ns |
| Panel2_MFI_HLA-DR_2316_CD4+.Tem_HLA-DR+ | -1487.90 | HIC | non_HIC | 0.25 | Wilcoxon | 0.46 | ns |
| Panel2_MFI_CD38_2232_CD56+CD3+_CD38+ | -445.49 | HIC | non_HIC | 0.26 | Wilcoxon | 0.47 | ns |
| Panel2_MFI_CXCR4_2637_NKcells_CXCR4+ | 662.91 | HIC | non_HIC | 0.27 | Wilcoxon | 0.48 | ns |
| Panel2_MFI_HLA-DR_2290_CD56+CD3+_HLA-DR+ | -1485.53 | HIC | non_HIC | 0.27 | Wilcoxon | 0.48 | ns |
| Panel2_MFI_CXCR4_2100_CD8+CXCR4+CCR5+ | 1349.25 | HIC | non_HIC | 0.28 | Wilcoxon | 0.48 | ns |
| Panel2_MFI_CXCR4_2647_mTreg_CXCR4+ | 1122.27 | HIC | non_HIC | 0.28 | Wilcoxon | 0.48 | ns |
| Panel2_MFI_PD1_2082_CD8+PD1+ | 561.47 | HIC | non_HIC | 0.29 | Wilcoxon | 0.49 | ns |
| Panel2_MFI_CD38_2233_CD4+CD8-_CD38+ | 196.74 | HIC | non_HIC | 0.33 | Wilcoxon | 0.54 | ns |
| Panel2_MFI_HLA-DR_2086_CD4+HLA-DR-CD38+ | 278.18 | HIC | non_HIC | 0.32 | Wilcoxon | 0.54 | ns |
| Panel2_MFI_HLA-DR_2090_CD8+HLA-DR-CD38+ | 365.76 | HIC | non_HIC | 0.32 | Wilcoxon | 0.54 | ns |
| Panel2_MFI_CXCR4_2662_CD4+.Tnaive_CXCR4+ | 2589.70 | HIC | non_HIC | 0.34 | Wilcoxon | 0.56 | ns |
| Panel2_MFI_CXCR4_2671_CD4+.Th17_CXCR4+ | 989.38 | HIC | non_HIC | 0.36 | Wilcoxon | 0.57 | ns |
| Panel2_MFI_CXCR4_2682_CD8+.Tnaive_CXCR4+ | 3054.54 | HIC | non_HIC | 0.37 | Wilcoxon | 0.57 | ns |
| Panel2_MFI_CXCR4_2686_CD8+.Tc2_CXCR4+ | 1005.61 | HIC | non_HIC | 0.36 | Wilcoxon | 0.57 | ns |
| Panel2_MFI_PD1_2119_CD4+CD8+_PD1+ | -738.66 | HIC | non_HIC | 0.36 | Wilcoxon | 0.57 | ns |
| Panel2_MFI_CCR5_2405_NKcells_CCR5+ | -420.06 | HIC | non_HIC | 0.38 | Wilcoxon | 0.59 | ns |
| Panel2_MFI_CXCR4_2099_CD8+CXCR4+CCR5- | 1584.04 | HIC | non_HIC | 0.39 | Wilcoxon | 0.59 | ns |
| Panel2_MFI_CXCR4_2667_Tfh_17_CXCR4+ | 1058.51 | HIC | non_HIC | 0.39 | Wilcoxon | 0.59 | ns |
| Panel2_MFI_PD1_2078_CD4+PD1+ | -283.68 | HIC | non_HIC | 0.41 | Wilcoxon | 0.61 | ns |
| Panel2_MFI_CXCR4_2666_Tfh_2_CXCR4+ | 1109.71 | HIC | non_HIC | 0.42 | Wilcoxon | 0.62 | ns |
| Panel2_MFI_CD38_2086_CD4+HLA-DR-CD38+ | 167.36 | HIC | non_HIC | 0.43 | Wilcoxon | 0.62 | ns |
| Panel2_MFI_CXCR4_2669_CD4+.Th1_CXCR4+ | 931.47 | HIC | non_HIC | 0.43 | Wilcoxon | 0.62 | ns |
| Panel2_MFI_PD1_2126_nTreg_PD1+ | -740.64 | HIC | non_HIC | 0.43 | Wilcoxon | 0.62 | ns |
| Panel2_MFI_CD38_2255_CD4+.Tcm_CD38+ | 159.10 | HIC | non_HIC | 0.44 | Wilcoxon | 0.62 | ns |
| Panel2_MFI_CXCR4_2681_CD8+.Tcm_CXCR4+ | 1421.81 | HIC | non_HIC | 0.44 | Wilcoxon | 0.63 | ns |
| Panel2_MFI_CD38_2265_CD4+.Th17_CD38+ | 99.27 | HIC | non_HIC | 0.45 | Wilcoxon | 0.63 | ns |
| Panel2_MFI_CD38_2231_NKcells_CD38+ | 922.65 | HIC | non_HIC | 0.47 | Wilcoxon | 0.64 | ns |
| Panel2_MFI_CXCR4_2648_nTreg_CXCR4+ | 1530.39 | HIC | non_HIC | 0.47 | Wilcoxon | 0.64 | ns |
| Panel2_MFI_HLA-DR_2300_nTreg_HLA-DR+ | 8876.85 | HIC | non_HIC | 0.47 | Wilcoxon | 0.64 | ns |
| Panel2_MFI_HLA-DR_2321_CD4+.Th1_HLA-DR+ | -970.55 | HIC | non_HIC | 0.46 | Wilcoxon | 0.64 | ns |
| Panel2_MFI_PD1_2141_CD4+.Temra_PD1+ | -738.25 | HIC | non_HIC | 0.48 | Wilcoxon | 0.64 | ns |
| Panel2_MFI_HLA-DR_2315_CD4+.Temra_HLA-DR+ | -2378.70 | HIC | non_HIC | 0.49 | Wilcoxon | 0.65 | ns |
| Panel2_MFI_CCR5_2438_CD4+.Th2_CCR5+ | 502.08 | HIC | non_HIC | 0.50 | Wilcoxon | 0.65 | ns |
| Panel2_MFI_CD38_2091_CD8+HLA-DR+CD38- | -58.33 | HIC | non_HIC | 0.52 | Wilcoxon | 0.65 | ns |
| Panel2_MFI_CD38_2256_CD4+.Tnaive_CD38+ | -187.95 | HIC | non_HIC | 0.52 | Wilcoxon | 0.65 | ns |
| Panel2_MFI_CD38_2259_Tfh_1_CD38+ | 129.72 | HIC | non_HIC | 0.52 | Wilcoxon | 0.65 | ns |
| Panel2_MFI_CD38_2260_Tfh_2_CD38+ | 171.57 | HIC | non_HIC | 0.52 | Wilcoxon | 0.65 | ns |
| Panel2_MFI_CXCR4_2665_Tfh_1_CXCR4+ | 1283.10 | HIC | non_HIC | 0.50 | Wilcoxon | 0.65 | ns |
| Panel2_MFI_PD1_2144_Tfh_2_PD1+ | 422.84 | HIC | non_HIC | 0.52 | Wilcoxon | 0.65 | ns |
| Panel2_MFI_CXCR4_2688_CD8+.Tc1/17_CXCR4+ | 825.35 | HIC | non_HIC | 0.54 | Wilcoxon | 0.67 | ns |
| Panel2_MFI_CD38_2087_CD4+HLA-DR+CD38- | 61.66 | HIC | non_HIC | 0.55 | Wilcoxon | 0.67 | ns |
| Panel2_MFI_PD1_2030_Tfh | 283.54 | HIC | non_HIC | 0.55 | Wilcoxon | 0.67 | ns |
| Panel2_MFI_HLA-DR_2087_CD4+HLA-DR+CD38- | -655.82 | HIC | non_HIC | 0.57 | Wilcoxon | 0.68 | ns |
| Panel2_MFI_HLA-DR_2334_CD8+.Tnaive_HLA-DR+ | -1032.25 | HIC | non_HIC | 0.57 | Wilcoxon | 0.68 | ns |
| Panel2_MFI_PD1_2150_CD4+.Th1/17_PD1+ | -212.05 | HIC | non_HIC | 0.57 | Wilcoxon | 0.68 | ns |
| Panel2_MFI_CD38_2092_CD8+HLA-DR+CD38+ | 170.46 | HIC | non_HIC | 0.58 | Wilcoxon | 0.69 | ns |
| Panel2_MFI_CXCR4_2672_CD4+.Th1/17_CXCR4+ | 547.32 | HIC | non_HIC | 0.62 | Wilcoxon | 0.72 | ns |
| Panel2_MFI_CXCR4_2096_CD4+CXCR4+CCR5+ | 393.22 | HIC | non_HIC | 0.63 | Wilcoxon | 0.73 | ns |
| Panel2_MFI_PD1_2125_mTreg_PD1+ | -209.41 | HIC | non_HIC | 0.64 | Wilcoxon | 0.74 | ns |
| Panel2_MFI_PD1_2146_Tfh_1/17_PD1+ | 213.01 | HIC | non_HIC | 0.67 | Wilcoxon | 0.77 | ns |
| Panel2_MFI_CXCR4_2668_Tfh_1/17_CXCR4+ | 710.95 | HIC | non_HIC | 0.68 | Wilcoxon | 0.78 | ns |
| Panel2_MFI_PD1_2139_CD4+.Tcm_PD1+ | -122.16 | HIC | non_HIC | 0.69 | Wilcoxon | 0.78 | ns |
| Panel2_MFI_PD1_2145_Tfh_17_PD1+ | -128.83 | HIC | non_HIC | 0.70 | Wilcoxon | 0.79 | ns |
| Panel2_MFI_CXCR4_2663_CD4+.Temra_CXCR4+ | -367.06 | HIC | non_HIC | 0.72 | Wilcoxon | 0.80 | ns |
| Panel2_MFI_CCR5_2450_CD8+.Tnaive_CCR5+ | -299.89 | HIC | non_HIC | 0.75 | Wilcoxon | 0.82 | ns |
| Panel2_MFI_CD38_2257_CD4+.Temra_CD38+ | 126.14 | HIC | non_HIC | 0.74 | Wilcoxon | 0.82 | ns |
| Panel2_MFI_PD1_2142_CD4+.Tem_PD1+ | -155.47 | HIC | non_HIC | 0.75 | Wilcoxon | 0.82 | ns |
| Panel2_MFI_CD38_2088_CD4+HLA-DR+CD38+ | -82.43 | HIC | non_HIC | 0.82 | Wilcoxon | 0.88 | ns |
| Panel2_MFI_CD38_2261_Tfh_17_CD38+ | -44.75 | HIC | non_HIC | 0.82 | Wilcoxon | 0.88 | ns |
| Panel2_MFI_PD1_2124_Treg_PD1+ | -102.76 | HIC | non_HIC | 0.83 | Wilcoxon | 0.88 | ns |
| Panel2_MFI_PD1_2116_CD56+CD3+_PD1+ | -114.97 | HIC | non_HIC | 0.84 | Wilcoxon | 0.88 | ns |
| Panel2_MFI_PD1_2148_CD4+.Th2_PD1+ | -88.11 | HIC | non_HIC | 0.84 | Wilcoxon | 0.88 | ns |
| Panel2_MFI_CD38_2262_Tfh_1/17_CD38+ | 30.32 | HIC | non_HIC | 0.88 | Wilcoxon | 0.91 | ns |
| Panel2_MFI_PD1_2143_Tfh_1_PD1+ | 85.99 | HIC | non_HIC | 0.89 | Wilcoxon | 0.92 | ns |
| Panel2_MFI_PD1_2147_CD4+.Th1_PD1+ | -66.60 | HIC | non_HIC | 0.91 | Wilcoxon | 0.94 | ns |
| Panel2_MFI_CXCR4_2098_CD8+CXCR4-CCR5+ | 44.21 | HIC | non_HIC | 0.93 | Wilcoxon | 0.94 | ns |
| Panel2_MFI_CXCR4_2641_CD4+CD8+_CXCR4+ | 104.41 | HIC | non_HIC | 0.93 | Wilcoxon | 0.94 | ns |
| Panel2_MFI_CXCR4_2638_CD56+CD3+_CXCR4+ | -47.83 | HIC | non_HIC | 0.95 | Wilcoxon | 0.95 | ns |
| Panel2_MFI_PD1_2149_CD4+.Th17_PD1+ | -24.95 | HIC | non_HIC | 0.95 | Wilcoxon | 0.95 | ns |

Significance differences in the expression levels of activation and exhaustion markers on T cells subsets between HIC and non-HIC were tested by Unpaired two­tailed *Wilcoxon Test*. The Benjamini–Hochberg method was used for multiple correction (FDR < 0.05) and adjusted *P* values <0.05 were considered significant.

**Supplementary TABLE 9. Comparison of expression levels (MFI) of PD1, HLA-DR, CD38, CXCR4 and CCR5 on T cells between EC (Elite controllers) and non-HIC (non-HIV controllers) from the discovery cohort.**

| cell_subset | estimate | group1 | group2 | p-value | method | p_adj | p.adj.signif |
| --- | --- | --- | --- | --- | --- | --- | --- |
| Panel2_MFI_CD38_2275_CD8+.Tcm_CD38+ | 273 8.99 | EC | non_HIC | 0.00 | Wilcoxon | 0.05 | ns |
| Panel2_MFI_CD38_2234_CD4-CD8+_CD38+ | 50 3.26 | EC | non_HIC | 0.01 | Wilcoxon | 0.17 | ns |
| Panel2_MFI_CD38_2278_CD8+.Tem_CD38+ | 100 4.45 | EC | non_HIC | 0.00 | Wilcoxon | 0.17 | ns |
| Panel2_MFI_CD38_2279_CD8+.Tc1_CD38+ | 125 6.53 | EC | non_HIC | 0.00 | Wilcoxon | 0.17 | ns |
| Panel2_MFI_CXCR4_2094_CD4+CXCR4-CCR5+ | 208 1.15 | EC | non_HIC | 0.01 | Wilcoxon | 0.17 | ns |
| Panel2_MFI_CXCR4_2095_CD4+CXCR4+CCR5- | 744 5.19 | EC | non_HIC | 0.01 | Wilcoxon | 0.17 | ns |
| Panel2_MFI_CXCR4_2100_CD8+CXCR4+CCR5+ | 633 8.14 | EC | non_HIC | 0.01 | Wilcoxon | 0.17 | ns |
| Panel2_MFI_CXCR4_2639_CD4+CD8-_CXCR4+ | 754 2.11 | EC | non_HIC | 0.01 | Wilcoxon | 0.17 | ns |
| Panel2_MFI_CXCR4_2661_CD4+.Tcm_CXCR4+ | 774 8.49 | EC | non_HIC | 0.01 | Wilcoxon | 0.17 | ns |
| Panel2_MFI_CXCR4_2664_CD4+.Tem_CXCR4+ | 294 8.75 | EC | non_HIC | 0.01 | Wilcoxon | 0.17 | ns |
| Panel2_MFI_CXCR4_2669_CD4+.Th1_CXCR4+ | 556 9.81 | EC | non_HIC | 0.01 | Wilcoxon | 0.17 | ns |
| Panel2_MFI_CXCR4_2684_CD8+.Tem_CXCR4+ | 603 6.01 | EC | non_HIC | 0.00 | Wilcoxon | 0.17 | ns |
| Panel2_MFI_CXCR4_2685_CD8+.Tc1_CXCR4+ | 619 6.70 | EC | non_HIC | 0.01 | Wilcoxon | 0.17 | ns |
| Panel2_MFI_CXCR4_2686_CD8+.Tc2_CXCR4+ | 509 8.77 | EC | non_HIC | 0.01 | Wilcoxon | 0.17 | ns |
| Panel2_MFI_HLA-DR_2289_NKcells_HLA-DR+ | 711 1.16 | EC | non_HIC | 0.01 | Wilcoxon | 0.17 | ns |
| Panel2_MFI_HLA-DR_2299_mTreg_HLA-DR+ | -747 0.80 | EC | non_HIC | 0.01 | Wilcoxon | 0.17 | ns |
| Panel2_MFI_CXCR4_2670_CD4+.Th2_CXCR4+ | 561 8.42 | EC | non_HIC | 0.01 | Wilcoxon | 0.17 | ns |
| Panel2_MFI_HLA-DR_2333_CD8+.Tcm_HLA-DR+ | 734 7.13 | EC | non_HIC | 0.02 | Wilcoxon | 0.17 | ns |
| Panel2_MFI_CXCR4_2648_nTreg_CXCR4+ | 851 4.41 | EC | non_HIC | 0.02 | Wilcoxon | 0.18 | ns |
| Panel2_MFI_HLA-DR_2335_CD8+.Temra_HLA-DR+ | 281 3.46 | EC | non_HIC | 0.02 | Wilcoxon | 0.18 | ns |
| Panel2_MFI_CD38_2280_CD8+.Tc2_CD38+ | 77 4.65 | EC | non_HIC | 0.03 | Wilcoxon | 0.19 | ns |
| Panel2_MFI_CXCR4_2098_CD8+CXCR4-CCR5+ | 161 9.59 | EC | non_HIC | 0.03 | Wilcoxon | 0.19 | ns |
| Panel2_MFI_CXCR4_2640_CD4-CD8+_CXCR4+ | 604 0.81 | EC | non_HIC | 0.03 | Wilcoxon | 0.19 | ns |
| Panel2_MFI_CXCR4_2646_Treg_CXCR4+ | 468 8.76 | EC | non_HIC | 0.02 | Wilcoxon | 0.19 | ns |
| Panel2_MFI_CXCR4_2647_mTreg_CXCR4+ | 334 6.79 | EC | non_HIC | 0.03 | Wilcoxon | 0.19 | ns |
| Panel2_MFI_CXCR4_2662_CD4+.Tnaive_CXCR4+ | 972 1.20 | EC | non_HIC | 0.03 | Wilcoxon | 0.19 | ns |
| Panel2_MFI_CXCR4_2665_Tfh_1_CXCR4+ | 771 4.57 | EC | non_HIC | 0.03 | Wilcoxon | 0.19 | ns |
| Panel2_MFI_CXCR4_2683_CD8+.Temra_CXCR4+ | 426 6.88 | EC | non_HIC | 0.02 | Wilcoxon | 0.19 | ns |
| Panel2_MFI_CXCR4_2688_CD8+.Tc1/17_CXCR4+ | 580 1.69 | EC | non_HIC | 0.03 | Wilcoxon | 0.19 | ns |
| Panel2_MFI_HLA-DR_2322_CD4+.Th2_HLA-DR+ | -644 6.61 | EC | non_HIC | 0.03 | Wilcoxon | 0.19 | ns |
| Panel2_MFI_PD1_2140_CD4+.Tnaive_PD1+ | -130 6.59 | EC | non_HIC | 0.03 | Wilcoxon | 0.19 | ns |
| Panel2_MFI_CD38_2263_CD4+.Th1_CD38+ | 82 5.50 | EC | non_HIC | 0.04 | Wilcoxon | 0.19 | ns |
| Panel2_MFI_CD38_2087_CD4+HLA-DR+CD38- | -32 5.45 | EC | non_HIC | 0.05 | Wilcoxon | 0.21 | ns |
| Panel2_MFI_CD38_2091_CD8+HLA-DR+CD38- | -29 8.87 | EC | non_HIC | 0.06 | Wilcoxon | 0.21 | ns |
| Panel2_MFI_CD38_2257_CD4+.Temra_CD38+ | 124 6.06 | EC | non_HIC | 0.04 | Wilcoxon | 0.21 | ns |
| Panel2_MFI_CD38_2276_CD8+.Tnaive_CD38+ | 31 4.55 | EC | non_HIC | 0.05 | Wilcoxon | 0.21 | ns |
| Panel2_MFI_CXCR4_2663_CD4+.Temra_CXCR4+ | 323 6.03 | EC | non_HIC | 0.06 | Wilcoxon | 0.21 | ns |
| Panel2_MFI_CXCR4_2667_Tfh_17_CXCR4+ | 427 3.81 | EC | non_HIC | 0.06 | Wilcoxon | 0.21 | ns |
| Panel2_MFI_CXCR4_2671_CD4+.Th17_CXCR4+ | 370 8.22 | EC | non_HIC | 0.05 | Wilcoxon | 0.21 | ns |
| Panel2_MFI_CXCR4_2681_CD8+.Tcm_CXCR4+ | 671 4.76 | EC | non_HIC | 0.05 | Wilcoxon | 0.21 | ns |
| Panel2_MFI_CXCR4_2682_CD8+.Tnaive_CXCR4+ | 1143 6.50 | EC | non_HIC | 0.05 | Wilcoxon | 0.21 | ns |
| Panel2_MFI_HLA-DR_2340_CD8+.Tc1/17_HLA-DR+ | 574 1.76 | EC | non_HIC | 0.05 | Wilcoxon | 0.21 | ns |
| Panel2_MFI_PD1_2160_CD8+.Tnaive_PD1+ | -91 5.35 | EC | non_HIC | 0.06 | Wilcoxon | 0.21 | ns |
| Panel2_MFI_CXCR4_2096_CD4+CXCR4+CCR5+ | 282 2.78 | EC | non_HIC | 0.06 | Wilcoxon | 0.21 | ns |
| Panel2_MFI_CXCR4_2666_Tfh_2_CXCR4+ | 433 3.39 | EC | non_HIC | 0.06 | Wilcoxon | 0.21 | ns |
| Panel2_MFI_HLA-DR_2315_CD4+.Temra_HLA-DR+ | 1040 8.93 | EC | non_HIC | 0.06 | Wilcoxon | 0.22 | ns |
| Panel2_MFI_CXCR4_2637_NKcells_CXCR4+ | 188 3.54 | EC | non_HIC | 0.07 | Wilcoxon | 0.22 | ns |
| Panel2_MFI_CXCR4_2668_Tfh_1/17_CXCR4+ | 575 7.49 | EC | non_HIC | 0.07 | Wilcoxon | 0.22 | ns |
| Panel2_MFI_CXCR4_2672_CD4+.Th1/17_CXCR4+ | 356 3.14 | EC | non_HIC | 0.07 | Wilcoxon | 0.22 | ns |
| Panel2_MFI_HLA-DR_2313_CD4+.Tcm_HLA-DR+ | -412 7.76 | EC | non_HIC | 0.07 | Wilcoxon | 0.23 | ns |
| Panel2_MFI_HLA-DR_2292_CD4-CD8+_HLA-DR+ | 275 3.07 | EC | non_HIC | 0.08 | Wilcoxon | 0.23 | ns |
| Panel2_MFI_CD38_2231_NKcells_CD38+ | 357 6.58 | EC | non_HIC | 0.09 | Wilcoxon | 0.25 | ns |
| Panel2_MFI_HLA-DR_2091_CD8+HLA-DR+CD38- | 209 9.76 | EC | non_HIC | 0.09 | Wilcoxon | 0.25 | ns |
| Panel2_MFI_CXCR4_2099_CD8+CXCR4+CCR5- | 505 8.51 | EC | non_HIC | 0.09 | Wilcoxon | 0.27 | ns |
| Panel2_MFI_HLA-DR_2323_CD4+.Th17_HLA-DR+ | -409 9.49 | EC | non_HIC | 0.11 | Wilcoxon | 0.30 | ns |
| Panel2_MFI_HLA-DR_2337_CD8+.Tc1_HLA-DR+ | 328 8.71 | EC | non_HIC | 0.11 | Wilcoxon | 0.30 | ns |
| Panel2_MFI_CXCR4_2641_CD4+CD8+_CXCR4+ | 316 9.23 | EC | non_HIC | 0.11 | Wilcoxon | 0.30 | ns |
| Panel2_MFI_HLA-DR_2336_CD8+.Tem_HLA-DR+ | 301 6.93 | EC | non_HIC | 0.13 | Wilcoxon | 0.33 | ns |
| Panel2_MFI_PD1_2126_nTreg_PD1+ | 215 1.36 | EC | non_HIC | 0.13 | Wilcoxon | 0.35 | ns |
| Panel2_MFI_HLA-DR_2298_Treg_HLA-DR+ | -466 5.92 | EC | non_HIC | 0.15 | Wilcoxon | 0.39 | ns |
| Panel2_MFI_HLA-DR_2300_nTreg_HLA-DR+ | 2723 6.05 | EC | non_HIC | 0.16 | Wilcoxon | 0.39 | ns |
| Panel2_MFI_CD38_2090_CD8+HLA-DR-CD38+ | 23 8.52 | EC | non_HIC | 0.18 | Wilcoxon | 0.45 | ns |
| Panel2_MFI_PD1_2164_CD8+.Tc2_PD1+ | 257 2.39 | EC | non_HIC | 0.20 | Wilcoxon | 0.48 | ns |
| Panel2_MFI_CXCR4_2638_CD56+CD3+_CXCR4+ | 240 0.84 | EC | non_HIC | 0.21 | Wilcoxon | 0.50 | ns |
| Panel2_MFI_CCR5_2408_CD4-CD8+_CCR5+ | -141 9.16 | EC | non_HIC | 0.23 | Wilcoxon | 0.54 | ns |
| Panel2_MFI_CD38_2092_CD8+HLA-DR+CD38+ | 54 7.10 | EC | non_HIC | 0.25 | Wilcoxon | 0.54 | ns |
| Panel2_MFI_HLA-DR_2092_CD8+HLA-DR+CD38+ | 434 1.40 | EC | non_HIC | 0.24 | Wilcoxon | 0.54 | ns |
| Panel2_MFI_PD1_2146_Tfh_1/17_PD1+ | 119 7.58 | EC | non_HIC | 0.25 | Wilcoxon | 0.54 | ns |
| Panel2_MFI_PD1_2149_CD4+.Th17_PD1+ | -62 6.30 | EC | non_HIC | 0.24 | Wilcoxon | 0.54 | ns |
| Panel2_MFI_PD1_2143_Tfh_1_PD1+ | 134 7.44 | EC | non_HIC | 0.26 | Wilcoxon | 0.56 | ns |
| Panel2_MFI_CCR5_2451_CD8+.Temra_CCR5+ | -146 0.33 | EC | non_HIC | 0.27 | Wilcoxon | 0.57 | ns |
| Panel2_MFI_CD38_2277_CD8+.Temra_CD38+ | 30 6.27 | EC | non_HIC | 0.27 | Wilcoxon | 0.57 | ns |
| Panel2_MFI_HLA-DR_2087_CD4+HLA-DR+CD38- | 224 8.59 | EC | non_HIC | 0.27 | Wilcoxon | 0.57 | ns |
| Panel2_MFI_PD1_2145_Tfh_17_PD1+ | -55 8.85 | EC | non_HIC | 0.29 | Wilcoxon | 0.59 | ns |
| Panel2_MFI_CCR5_2439_CD4+.Th17_CCR5+ | -75 1.05 | EC | non_HIC | 0.30 | Wilcoxon | 0.60 | ns |
| Panel2_MFI_PD1_2030_Tfh | 85 7.60 | EC | non_HIC | 0.30 | Wilcoxon | 0.60 | ns |
| Panel2_MFI_PD1_2166_CD8+.Tc1/17_PD1+ | 87 2.86 | EC | non_HIC | 0.30 | Wilcoxon | 0.60 | ns |
| Panel2_MFI_CCR5_2096_CD4+CXCR4+CCR5+ | -107 0.38 | EC | non_HIC | 0.31 | Wilcoxon | 0.60 | ns |
| Panel2_MFI_CCR5_2452_CD8+.Tem_CCR5+ | -111 1.49 | EC | non_HIC | 0.32 | Wilcoxon | 0.60 | ns |
| Panel2_MFI_HLA-DR_2291_CD4+CD8-_HLA-DR+ | -269 6.82 | EC | non_HIC | 0.31 | Wilcoxon | 0.60 | ns |
| Panel2_MFI_CCR5_2100_CD8+CXCR4+CCR5+ | -120 0.35 | EC | non_HIC | 0.33 | Wilcoxon | 0.61 | ns |
| Panel2_MFI_HLA-DR_2314_CD4+.Tnaive_HLA-DR+ | -209 0.23 | EC | non_HIC | 0.33 | Wilcoxon | 0.61 | ns |
| Panel2_MFI_PD1_2078_CD4+PD1+ | -50 5.22 | EC | non_HIC | 0.33 | Wilcoxon | 0.61 | ns |
| Panel2_MFI_CCR5_2098_CD8+CXCR4-CCR5+ | -101 3.91 | EC | non_HIC | 0.35 | Wilcoxon | 0.61 | ns |
| Panel2_MFI_CCR5_2453_CD8+.Tc1_CCR5+ | -96 4.33 | EC | non_HIC | 0.35 | Wilcoxon | 0.61 | ns |
| Panel2_MFI_CD38_2088_CD4+HLA-DR+CD38+ | 47 9.21 | EC | non_HIC | 0.34 | Wilcoxon | 0.61 | ns |
| Panel2_MFI_PD1_2116_CD56+CD3+_PD1+ | 87 8.90 | EC | non_HIC | 0.36 | Wilcoxon | 0.61 | ns |
| Panel2_MFI_PD1_2142_CD4+.Tem_PD1+ | -67 8.56 | EC | non_HIC | 0.36 | Wilcoxon | 0.61 | ns |
| Panel2_MFI_CCR5_2415_mTreg_CCR5+ | -73 9.60 | EC | non_HIC | 0.37 | Wilcoxon | 0.63 | ns |
| Panel2_MFI_CD38_2258_CD4+.Tem_CD38+ | 21 0.06 | EC | non_HIC | 0.37 | Wilcoxon | 0.63 | ns |
| Panel2_MFI_CCR5_2437_CD4+.Th1_CCR5+ | -67 2.89 | EC | non_HIC | 0.39 | Wilcoxon | 0.63 | ns |
| Panel2_MFI_CD38_2233_CD4+CD8-_CD38+ | 29 6.53 | EC | non_HIC | 0.39 | Wilcoxon | 0.63 | ns |
| Panel2_MFI_CD38_2255_CD4+.Tcm_CD38+ | 28 1.08 | EC | non_HIC | 0.39 | Wilcoxon | 0.63 | ns |
| Panel2_MFI_CD38_2265_CD4+.Th17_CD38+ | 21 7.62 | EC | non_HIC | 0.38 | Wilcoxon | 0.63 | ns |
| Panel2_MFI_PD1_2125_mTreg_PD1+ | -62 4.49 | EC | non_HIC | 0.39 | Wilcoxon | 0.63 | ns |
| Panel2_MFI_CD38_2086_CD4+HLA-DR-CD38+ | 28 5.96 | EC | non_HIC | 0.42 | Wilcoxon | 0.67 | ns |
| Panel2_MFI_CCR5_2406_CD56+CD3+_CCR5+ | -148 5.54 | EC | non_HIC | 0.46 | Wilcoxon | 0.70 | ns |
| Panel2_MFI_CD38_2232_CD56+CD3+_CD38+ | 52 2.83 | EC | non_HIC | 0.45 | Wilcoxon | 0.70 | ns |
| Panel2_MFI_CD38_2235_CD4+CD8+_CD38+ | 39 3.29 | EC | non_HIC | 0.46 | Wilcoxon | 0.70 | ns |
| Panel2_MFI_CCR5_2450_CD8+.Tnaive_CCR5+ | 123 6.61 | EC | non_HIC | 0.48 | Wilcoxon | 0.71 | ns |
| Panel2_MFI_PD1_2115_NKcells_PD1+ | 59 1.44 | EC | non_HIC | 0.49 | Wilcoxon | 0.71 | ns |
| Panel2_MFI_PD1_2119_CD4+CD8+_PD1+ | -88 8.51 | EC | non_HIC | 0.47 | Wilcoxon | 0.71 | ns |
| Panel2_MFI_PD1_2139_CD4+.Tcm_PD1+ | -31 4.74 | EC | non_HIC | 0.49 | Wilcoxon | 0.71 | ns |
| Panel2_MFI_CCR5_2456_CD8+.Tc1/17_CCR5+ | -167 2.68 | EC | non_HIC | 0.51 | Wilcoxon | 0.74 | ns |
| Panel2_MFI_CCR5_2440_CD4+.Th1/17_CCR5+ | 54 0.75 | EC | non_HIC | 0.52 | Wilcoxon | 0.75 | ns |
| Panel2_MFI_PD1_2147_CD4+.Th1_PD1+ | -66 6.86 | EC | non_HIC | 0.52 | Wilcoxon | 0.75 | ns |
| Panel2_MFI_CCR5_2407_CD4+CD8-_CCR5+ | -52 5.39 | EC | non_HIC | 0.53 | Wilcoxon | 0.75 | ns |
| Panel2_MFI_PD1_2141_CD4+.Temra_PD1+ | 89 1.92 | EC | non_HIC | 0.53 | Wilcoxon | 0.75 | ns |
| Panel2_MFI_PD1_2162_CD8+.Tem_PD1+ | 76 3.51 | EC | non_HIC | 0.54 | Wilcoxon | 0.75 | ns |
| Panel2_MFI_CD38_2264_CD4+.Th2_CD38+ | 19 2.11 | EC | non_HIC | 0.57 | Wilcoxon | 0.77 | ns |
| Panel2_MFI_HLA-DR_2334_CD8+.Tnaive_HLA-DR+ | 189 3.90 | EC | non_HIC | 0.56 | Wilcoxon | 0.77 | ns |
| Panel2_MFI_PD1_2159_CD8+.Tcm_PD1+ | -52 7.88 | EC | non_HIC | 0.57 | Wilcoxon | 0.77 | ns |
| Panel2_MFI_HLA-DR_2090_CD8+HLA-DR-CD38+ | 31 4.45 | EC | non_HIC | 0.59 | Wilcoxon | 0.78 | ns |
| Panel2_MFI_HLA-DR_2293_CD4+CD8+_HLA-DR+ | 111 5.56 | EC | non_HIC | 0.59 | Wilcoxon | 0.78 | ns |
| Panel2_MFI_CD38_2256_CD4+.Tnaive_CD38+ | -24 9.20 | EC | non_HIC | 0.60 | Wilcoxon | 0.79 | ns |
| Panel2_MFI_CCR5_2405_NKcells_CCR5+ | -34 5.73 | EC | non_HIC | 0.63 | Wilcoxon | 0.81 | ns |
| Panel2_MFI_CCR5_2429_CD4+.Tcm_CCR5+ | -34 6.65 | EC | non_HIC | 0.63 | Wilcoxon | 0.81 | ns |
| Panel2_MFI_PD1_2150_CD4+.Th1/17_PD1+ | -32 0.54 | EC | non_HIC | 0.63 | Wilcoxon | 0.81 | ns |
| Panel2_MFI_CCR5_2430_CD4+.Tnaive_CCR5+ | -128 7.12 | EC | non_HIC | 0.64 | Wilcoxon | 0.81 | ns |
| Panel2_MFI_CCR5_2454_CD8+.Tc2_CCR5+ | -65 5.85 | EC | non_HIC | 0.67 | Wilcoxon | 0.84 | ns |
| Panel2_MFI_HLA-DR_2324_CD4+.Th1/17_HLA-DR+ | -96 9.56 | EC | non_HIC | 0.67 | Wilcoxon | 0.84 | ns |
| Panel2_MFI_CD38_2261_Tfh_17_CD38+ | 12 2.65 | EC | non_HIC | 0.69 | Wilcoxon | 0.85 | ns |
| Panel2_MFI_CD38_2266_CD4+.Th1/17_CD38+ | 8 5.31 | EC | non_HIC | 0.69 | Wilcoxon | 0.85 | ns |
| Panel2_MFI_CCR5_2094_CD4+CXCR4-CCR5+ | -31 7.08 | EC | non_HIC | 0.69 | Wilcoxon | 0.85 | ns |
| Panel2_MFI_HLA-DR_2088_CD4+HLA-DR+CD38+ | -240 7.46 | EC | non_HIC | 0.70 | Wilcoxon | 0.85 | ns |
| Panel2_MFI_HLA-DR_2338_CD8+.Tc2_HLA-DR+ | 73 6.52 | EC | non_HIC | 0.73 | Wilcoxon | 0.87 | ns |
| Panel2_MFI_PD1_2144_Tfh_2_PD1+ | 34 4.45 | EC | non_HIC | 0.73 | Wilcoxon | 0.87 | ns |
| Panel2_MFI_PD1_2148_CD4+.Th2_PD1+ | -21 8.00 | EC | non_HIC | 0.72 | Wilcoxon | 0.87 | ns |
| Panel2_MFI_CCR5_2099_CD8+CXCR4+CCR5- | -16 7.47 | EC | non_HIC | 0.77 | Wilcoxon | 0.90 | ns |
| Panel2_MFI_HLA-DR_2086_CD4+HLA-DR-CD38+ | -11 2.27 | EC | non_HIC | 0.77 | Wilcoxon | 0.90 | ns |
| Panel2_MFI_CD38_2260_Tfh_2_CD38+ | 11 7.64 | EC | non_HIC | 0.78 | Wilcoxon | 0.90 | ns |
| Panel2_MFI_CCR5_2438_CD4+.Th2_CCR5+ | 33 4.18 | EC | non_HIC | 0.80 | Wilcoxon | 0.91 | ns |
| Panel2_MFI_CCR5_2414_Treg_CCR5+ | 22 1.25 | EC | non_HIC | 0.81 | Wilcoxon | 0.91 | ns |
| Panel2_MFI_CCR5_2432_CD4+.Tem_CCR5+ | 18 6.48 | EC | non_HIC | 0.82 | Wilcoxon | 0.91 | ns |
| Panel2_MFI_PD1_2124_Treg_PD1+ | -19 4.60 | EC | non_HIC | 0.81 | Wilcoxon | 0.91 | ns |
| Panel2_MFI_PD1_2161_CD8+.Temra_PD1+ | 16 5.37 | EC | non_HIC | 0.82 | Wilcoxon | 0.91 | ns |
| Panel2_MFI_CCR5_2409_CD4+CD8+_CCR5+ | 24 0.52 | EC | non_HIC | 0.86 | Wilcoxon | 0.93 | ns |
| Panel2_MFI_CCR5_2416_nTreg_CCR5+ | -63 9.13 | EC | non_HIC | 0.88 | Wilcoxon | 0.93 | ns |
| Panel2_MFI_CCR5_2431_CD4+.Temra_CCR5+ | 74 2.93 | EC | non_HIC | 0.86 | Wilcoxon | 0.93 | ns |
| Panel2_MFI_CCR5_2449_CD8+.Tcm_CCR5+ | 14 5.10 | EC | non_HIC | 0.88 | Wilcoxon | 0.93 | ns |
| Panel2_MFI_CD38_2240_Treg_CD38+ | -7 8.74 | EC | non_HIC | 0.87 | Wilcoxon | 0.93 | ns |
| Panel2_MFI_CD38_2259_Tfh_1_CD38+ | 4 4.15 | EC | non_HIC | 0.88 | Wilcoxon | 0.93 | ns |
| Panel2_MFI_HLA-DR_2316_CD4+.Tem_HLA-DR+ | -29 1.88 | EC | non_HIC | 0.88 | Wilcoxon | 0.93 | ns |
| Panel2_MFI_CD38_2242_nTreg_CD38+ | 4 7.51 | EC | non_HIC | 0.92 | Wilcoxon | 0.95 | ns |
| Panel2_MFI_PD1_2163_CD8+.Tc1_PD1+ | 10 9.79 | EC | non_HIC | 0.91 | Wilcoxon | 0.95 | ns |
| Panel2_MFI_CCR5_2095_CD4+CXCR4+CCR5- | 6 1.95 | EC | non_HIC | 0.92 | Wilcoxon | 0.95 | ns |
| Panel2_MFI_CD38_2262_Tfh_1/17_CD38+ | -2 7.94 | EC | non_HIC | 0.93 | Wilcoxon | 0.96 | ns |
| Panel2_MFI_HLA-DR_2290_CD56+CD3+_HLA-DR+ | -15 8.09 | EC | non_HIC | 0.94 | Wilcoxon | 0.96 | ns |
| Panel2_MFI_CD38_2241_mTreg_CD38+ | -3 1.80 | EC | non_HIC | 0.95 | Wilcoxon | 0.97 | ns |
| Panel2_MFI_PD1_2082_CD8+PD1+ | 3 2.65 | EC | non_HIC | 0.96 | Wilcoxon | 0.97 | ns |
| Panel2_MFI_HLA-DR_2321_CD4+.Th1_HLA-DR+ | 2 0.22 | EC | non_HIC | 0.99 | Wilcoxon | 0.99 | ns |

Significance differences in the expression levels of activation and exhaustion markers on T cells subsets between EC and non-HIC were tested by Unpaired two­tailed *Wilcoxon Test*. The Benjamini–Hochberg method was used for multiple correction (FDR < 0.05) and adjusted *P* values <0.05 were considered significant.

Supplementary Figure 1

Supplementary Figure 2

Supplementary Figure 3

**Supplementary Fig 1-3.** Manual annotation of immune cell populations identified in the 2000 HIV study across the three evaluated flow cytometry panels.

Supplementary Figure 4

**Supplementary Figure 4.** Histograms of inverse rank–transformed immune cell percentages included in linear regression analyses. Shown are significant hits from comparison between elite controllers (EC) and non–controllers (non-HIC) analysis.

Supplementary Figure 5


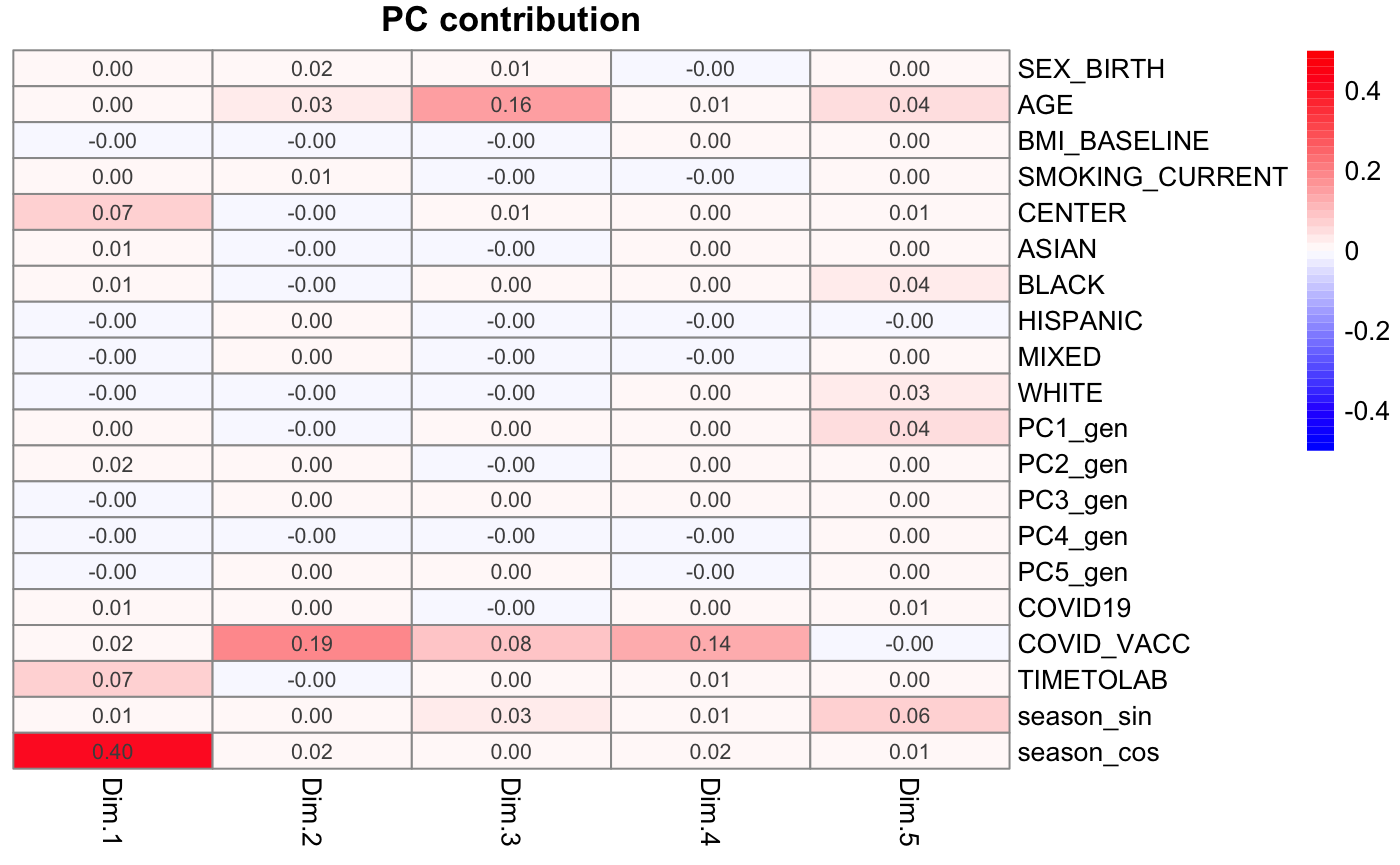


**Supplementary 5.** Heat map showing the association between the first five principal components derived from flow cytometry data (percentage) in the discovery cohort and pontential confounders. The color and values in the heat map represents the r^2^

Supplementary Figure 6

**Supplementary Figure 6.** Association analyses between the relative percentages of immune cells and HIC persistent control phenotype (HIC), and EC phenotype (EC). Comparisons between HIC (n=54) vs. non-HIC (n=1044) and EC (n=21) vs. non-HIC groups were tested using a linear regression model adjusted for age, sex-birth, time to lab, seasonality, and COVID-19 vaccination status. Heatmaps represent the estimates from the linear regression model, with significant associations indicated (*p < 0.05, **p < 0.0001).

Supplementary Figure 7

A

B

**Supplementary Figure 7. Unsupervised clustering analysis of CD45+ whole blood cells for deep characterization of TCRγδ cells in Elite Controllers (A)** Heat map displaying the expression intensity of markers used to define the 20 cell metaclusters via FlowSOM. Metaclusters 8 and metacluster 11 were identified as TCRgdv1 and TCRgdvd2, respectively. Scale color represents the Log_10_ MFI transformation per marker. (**B)** Box plots showing the differences in the abundance of the 20 metaclusters between between EC (n=21) vs non-HIC (n=21). Comparisons were performed using the Wilcoxon test (p-value < 0.05)

Supplementary Figure 8

**Supplementary Figure 8.** Boxplots showing the comparison of the frequencies of immune cells shown in Figure 3B-C between HIC (excluding ART-naïve EC, n=33) and EC, n=21. The center line represents the median, and the box extends from the first to the third quartile (interquartile range).
